# Systematic review about complementary medical hyperthermia in oncology

**DOI:** 10.1007/s10238-022-00846-9

**Published:** 2022-06-29

**Authors:** Christina Maria Liebl, Sabine Kutschan, Jennifer Dörfler, Lukas Käsmann, Jutta Hübner

**Affiliations:** 1 Klinik für Innere Medizin II, Hämatologie und Internistische Onkologie, Universitätsklinikum Jena, Am Klinikum 1, 07747 Jena, Germany; 2 Klinik und Poliklinik für Strahlentherapie, LMU Klinikum, Munich, Germany

**Keywords:** Whole-body hyperthermia, Extracorporeal circulation, Electro hyperthermia, Neoplasm

## Abstract

Hyperthermia is a generic term for different techniques using heat in cancer therapies. Temperatures of about 42° Celsius in combination with chemo- or radiotherapy may improve the effectiveness of those treatments. Clinical benefit is shown in “standard hyperthermia” with tumour temperatures assessed during treatment. This systematic review thoroughly assesses the state of evidence concerning the benefits and side effects of electro hyperthermia or whole-body hyperthermia (“alternative hyperthermia”) in oncology. From 26 April 2021 to 09 May 2021, a systematic search was conducted searching five electronic databases (Embase, Cochrane, PsycINFO, CINAHL and Medline) to find studies concerning the use, effectiveness and potential harm of alternative medical hyperthermia therapy on cancer patients. From all 47,388 search results, 53 publications concerning 53 studies with 2006 patients were included in this systematic review. The patients were diagnosed with different types of cancer. The hyperthermic methods included whole-body hyperthermia (WBH) with different methods and electro hyperthermia (EH). The majority of the included studies were single-arm studies, counting in total 32 studies. Six studies were randomized controlled trials (RCT). In addition, one systematic review (SR) was found. The most critical endpoints were tumour response, survival data, pain relief, myelosuppression and toxicities. Outcome was heterogeneous, and considering the methodological limitations, clinical evidence for the benefit of alternative hyperthermia in cancer patients is lacking. Neither for whole-body hyperthermia nor for electro hyperthermia there is any evidence with respect to improvement of survival or quality of life in cancer patients.

**Supplementary Information:**

The online version contains supplementary material available at 10.1007/s10238-022-00846-9.

## Introduction

For over 100 years, physicians have attempted to treat cancer patients by heating tumour tissue. While certain techniques allow to directly kill cancer cells by heat, for whole-body and electro hyperthermia, the rationale is that an increase in the temperature of the tumour (40–43 °C) [1] induces changes in perfusion and re-oxygenation, produces heat-shock proteins, stimulates immunological activity and thus improves tumour response to radio- and chemotherapy [2, 3]. Previous researches in conventional medicine concluded that heat and radiation may have synergistic effects [4], because cells in late S phase are resistant to radiation and studies found that they were additionally most sensitive to heat in that phase [5]. In addition, tumour tissue is characterized by nutrient deprivation, limited oxygenation and highly acid conditions [6]. These properties are connected with low effects on tumour response to radiation [7]. Temperatures between 40 and 42 °C may increase perfusion and thereby oxygenation increases [8] with the possibility to overcome hypoxia-associated radio-resistance [9]. On the one hand, the higher sensitivity to chemotherapy is a result of elevated tumour blood flow, resulting in higher therapeutic drug concentrations in the tumour tissue [10]. A clear temperature-dependent increase in drug uptake has been shown in preclinical trials for example for cisplatin [11], carboplatin [12] or Melphalan [10, 13]. On the other hand, hyperthermia is able to increase the number of DNA strand breaks induced by chemotherapy [10, 12, 14] and may inhibit DNA repair [15]. Moreover, induction of heat-shock proteins (HSP) and immunomodulation has been described [16].

Nowadays, different methods of hyperthermia may be distinguished [1]. Well-established usage is hyperthermic intraperitoneal chemotherapy (HIPEC) [17], the combination with chemotherapy in patients with sarcoma [18, 19] or the combination with radiotherapy for patients with cervical cancer who refuse or are not eligible for chemotherapy [20, 21]. Moreover, interstitial hyperthermia with brachytherapy is used for local applications [22]. Other methods like the activation of magnetic nanoparticles by an alternating magnetic field are currently being explored as a technique for targeted therapeutic heating of tumours [23]. Thermal ablation, which uses temperatures of above 100 °C to destroy tumour tissue directly, has to be differentiated from these types of hyperthermia treatment [24]. For hyperthermia treatments, the European Society of Hyperthermic Oncology (ESHO) and the Interdisziplinäre Arbeitsgruppe Hyperthermie (IAH) have defined quality standards [25–30]. A decisive criterion is that the temperature is measured directly in the tumour in order to verify heating of the target volume to the required 40–43 °C. This is an important point to differentiate between conventional and alternative methods, since no intra-tumoural temperature measurement is performed in the alternative hyperthermia methods.

Electro hyperthermia (EH) does not comply with the standards mentioned above and is widely spread. The method of capacitive coupling seems to be particularly popular because tumour cells may allegedly be heated selectively with this method. The therapy takes advantage of the supposedly special absorption rate of the extracellular fluid of the tumour. Tumour tissue is said to have a lower impedance than neighbouring tissue, so that energy is absorbed primarily by the tumour. This is supposed to achieve self-focusing [31]. A well-known representative of capacitive coupling is oncothermia [32]. This method uses radiofrequency waves with a frequency of 13.56 MHz [33].

Whole-body hyperthermia (WBH) has been evaluated in the 1950s to 1980s, but was left due to a negative risk/benefit ratio. So-called moderate whole-body hyperthermia is still used [34]. In alternative medicine, hyperthermia is used as WBH with moderate temperatures (about 39 °C) or high temperatures (more than 40 °C) [31]. Heating is reached by a warmed waterbed or by infrared radiation. Other alternative WBH procedures are the extracorporeal heating of blood or the induction of fever by bacterial toxins [3].

Therefore, we have included in this review only hyperthermia methods that do not belong to conventional medicine and titled these alternative methods. This includes all methods that heat the whole body either from the outside or from inside by using extracorporeal circulation or bacterial toxins. In addition, all hyperthermia methods with electric fields that do not meet the ESHO criteria are considered. Conventional other medical procedures with heat such as HIPEC [35], HIVEC [36] or thermoablation [24] are not part of our review. Moreover, hyperthermia generated by high-frequency radiofrequency waves or with microwaves [37] with an adequate real-time thermal dose monitoring [38, 39] meeting the requirements of the ESHO is also explicitly not part of our review [25–30].

The aim of our systematic review is to assess the evidence of these different alternative methods of external hyperthermia provided beyond the international standards.

## Method

### Inclusion and exclusion criteria

Inclusion and exclusion criteria are listed in Table 1 based on a PICO model. Generally, all study types were included if they reported patient-relevant outcomes after the treatment of adult cancer patients with a complementary medical hyperthermia. Complementary hyperthermia methods included any modalities whether whole-body or electro hyperthermia, because exactly these do not meet the defined quality criteria of the European Society of Hyperthermic Oncology [25–30]. Any kind of comparison was eligible in this review. This includes watch and wait, standard care, sham and placebo. Because of the wide range of application fields, all cancer entities were included. Since little high-quality evidence was expected, systematic reviews and randomized controlled trials were included as well as controlled trials, one-armed studies, case reports and case series. Criteria for rejecting studies were primary prevention, grey literature, other publication types than primary investigation/reports (e.g. comments, letters, abstracts) and study population with more than 20% children (patients under the age of 18) if results of adult patients with cancer were not reported separately or precancerous conditions. Additionally, systematic reviews, randomized controlled trials and cohort studies were excluded if they reported only not patient-centred outcomes at all (only labour parameters). Furthermore, we included single-arm studies, case reports and case series if side effects were reported. Language restrictions were made to English and German. Studies that evaluated a combination of hyperthermia and other treatments versus none of the parts of the combination were not included as it would not be possible to determine the impact of hyperthermia. Nevertheless, we analysed the side effects of hyperthermia treatment in these 43 studies and reported the side effects, which were clearly attributed to hyperthermia treatment.

**Table 1:** Inclusion and exclusion criteria

PICO	Inclusion criteria	Exclusion criteria
Patient	Cancer patients (all entities and stages) Adult patients	Patients with precancerous conditions or carcinoma in situ Study population with more than 20% children under the age of 18 Primary prevention Preclinical studies
Intervention	Every intervention with hyperthermia in complementary medicine (hyperthermia treatments, which do not meet the defined quality criteria of the European Society for Clinical Oncology: whole-body-hyperthermia, hyperthermia with extracorporeal circulation, electro hyperthermia)	
Comparison	All possible control groups (active control, placebo, standard, guideline, usual care, wait list)	
Outcome	Mortality (overall survival) Morbidity (progression/disease-free interval, tumour response) Patient-reported outcomes (i.e. quality of life or other important psychological outcomes) Toxicity and adverse events (CTCAE) Laboratory parameters	
Others	Language: German and English Full publication	Gray literature (conference articles, abstracts, letters, ongoing studies, unpublished literature…)

### Study selection

A systematic review was conducted using five databases (Medline (Ovid), CINAHL (EBSCO), EMBASE (Ovid), Cochrane CENTRAL and PsycINFO (EBSCO)) in April to May 2021. For each of these databases, a complex search strategy was developed consisting of a combination of MeshTerms, keywords and text words in different spellings connected to cancer and the different types of alternative hyperthermia therapy (Table 2). The search string was highly sensitive, since it was not restricted by filters of study or publication type. After importing the search results into EndNote X8, all duplicates were removed and a title–abstract screening was carried out by two independent reviewers (CL and SK). In case of disagreement, consensus was made by discussion. After that, all full texts were retrieved and screened again independently by both reviewers. When title and abstract did not have sufficient information for screening purposes, a full-text copy was retrieved as well. Additionally, bibliography lists of all retrieved articles were searched for relevant studies.

**Table 2:** Search strategies for each database

Database	Search strategy (26 April 2021–9 May 2021)
OVID Medline	1 Hyperthermia, Induced/or Steam bath/or hypertherm$.mp. or oncotherm$.mp. or thermotherap$.mp. or ((hot or heat) adj1 (therap$ or treatment or medical or pack or bath or immers$)).mp. or (fever adj1 therap$).mp. or (capacitive adj1 coupling).mp, 2 Exp neoplasms/or neoplasm$.mp or cancer$.mp. or tumo?r$.mp. or malignan$.mp. or oncolog$.mp. or carcinom$.mp. or leuk?emia.mp. or lymphom$.mp. or sarcom$.mp. or preneoplas$.mp. or exp Precancerous Conditions/or precancer$.mp 3 1 AND 2 4 Limit 3 to English or limit 3 to German 5 (4 and humans/) or (4 not animals/) 6 (((comprehensive* or integrative or systematic*) adj3 (bibliographic* or review* or literature)) or (meta-analy* or metaanaly* or "research synthesis" or ((information or data) adj3 synthesis) or (data adj2 extract*))).ti,ab. or (cinahl or (cochrane adj3 trial*) or embase or medline or psyclit or (psycinfo not "psycinfo database") or pubmed or scopus or "sociological abstracts" or "web of science" or central).ab. or ("cochrane database of systematic reviews" or evidence report technology assessment or evidence report technology assessment summary).jn. or Evidence Report: Technology Assessment*.jn. or network adj1 analy*.ti,ab.) or (((review adj5 (rationale or evidence)).ti,ab. and review.pt.) or meta-analysis as topic/or Meta-Analysis.pt.) 7 Randomized controlled trial.pt. or controlled clinical trial.pt. or randomi?ed.ti,ab.or placebo.ti,ab. or drug therapy.sh. or randomly.ti,ab. or trial?.ti,ab. or group?.ti,ab 8 5 AND (6 OR 7) 9 5 NOT 8
OVID Embase	1 Hyperthermia/or Experimental Hyperthermia/or Thermotherapy/or Pyrotherpy/or hypertherm$.mp. or oncotherm$.mp. or thermotherap$.mp. or ((hot or heat) adj1 (therap$ or treatment or medical or pack or bath or immers$)).mp. or (fever adj1 therap$).mp. or (capacitive adj1 coupling).mp 2 Exp neoplasms/or neoplasm$.mp or cancer$.mp. or tumo?r$.mp. or malignan$.mp. or oncolog$.mp. or carcinom$.mp. or leuk?emia.mp. or lymphom$.mp. or sarcom$.mp. or preneoplas$.mp. or exp Precancerous Conditions/or precancer$.mp 3 1 AND 2 4 Limit 3 to English or limit 3 to German 5 (4 and humans/) or (4 not animals/) 6 ((((comprehensive* or integrative or systematic*) adj3 (bibliographic* or review* or literature)) or (meta-analy* or metaanaly* or "research synthesis" or ((information or data) adj3 synthesis) or (data adj2 extract*))).ti,ab. or (cinahl or (cochrane adj3 trial*) or embase or medline or psyclit or (psycinfo not "psycinfo database") or pubmed or scopus or "sociological abstracts" or "web of science" or central).ab. or ("cochrane database of systematic reviews" or evidence report technology assessment or evidence report technology assessment summary).jn. or Evidence Report: Technology Assessment*.jn. or (network adj1 analy*).ti,ab.) or (exp Meta Analysis/or ((data extraction.ab. or selection criteria.ab.) and review.pt.)) 7 Crossover procedure/or double blind procedure/or randomized controlled trial/or single blind procedure/or (random$ or factorial$ or crossover$ or (cross adj1 over$) or placebo$ or (doubl$ adj1 blind$) or (singl$ adj1 blind$) or assign$ or allocat$ or volunteer$).ti,ab,de 8 5 AND (6 OR 7) 9 5 NOT 8
Cochrane	#1 [mh ^”Hyperthermia, Induced”] or [mh ^”Steam bath”] or hypertherm* or oncotherm* or thermotherap* or ((hot or heat) NEXT (therap* or treatment or medical or pack or bath or immers*)) or (fever NEXT therap*) or “capacitive coupling” #2 [mh neoplasms] or neoplasm* or cancer? or tum*r? or malignan* or oncolog* or carcinom* or leuk*mia or lymphoma? or sarcoma? or precancer* or preneoplas* #3 #1 AND #2
EBSCO PsycINFO	S1 DE Hyperthermia or TX (hypertherm* or oncotherm* or thermotherap* or ((hot or heat) N1 (therap* or treatment or medical or pack or bath or immers*)) or (fever N1 therap*) or “capacitive coupling”) S2 ((DE "Neoplasms" OR DE "Benign Neoplasms" OR DE "Breast Neoplasms" OR DE "Endocrine Neoplasms" OR DE "Leukemias" OR DE "Melanoma" OR DE "Metastasis" OR DE "Nervous System Neoplasms" OR DE "Terminal Cancer") OR (TX neoplasm* OR TX cancer OR TX tumo#r OR TX malignan* OR DE „oncology “ OR TX oncolog* OR TX carcinom* OR TX leuk#emia OR TX lymphoma OR TX sarcoma OR TX precancer* OR TX preneoplas*)) S3 LA (English OR German) S4 S1 AND S2 AND S3 S5 ((comprehensive* OR integrative OR systematic*) N3 (bibliographic* OR review* OR literature)) OR (meta-analy* or metaanaly* or "research synthesis" OR ((information OR data) N3 synthesis) OR (data N2 extract*)) OR ((review N5 (rationale OR evidence)) AND DE "Literature Review") OR (AB(cinahl OR (cochrane N3 trial*) OR embase OR medline OR psyclit OR pubmed OR scopus OR "sociological abstracts" OR "web of science" OR central)) OR DE "Meta Analysis" OR (network N1 analy*) S6 DE "Treatment Effectiveness Evaluation" OR DE "Treatment Outcomes" OR DE "Psychotherapeutic Outcomes" OR DE "Placebo" or DE "Followup Studies" OR placebo* OR random* OR "comparative stud*" OR (clinical N3 trial*) OR (research N3 design) OR (evaluat* N3 stud*) OR (prospectiv* N3 stud*) OR ((singl* OR doubl* OR trebl* OR tripl*) N3 (blind* OR mask*) S7 S4 AND (S5 OR S6) S8 S4 NOT S7
EBSCO CINAHL	S1 MH “Hyperthermia, Induced” or TX (hypertherm* or oncotherm* or thermotherap* or ((hot or heat) N1 (therap* or treatment or medical or pack or bath or immers*)) or (fever N1 therap*) or “capacitive coupling”) S2 (MH "Neoplasms + " OR TX neoplasm* OR TX cancer OR TX tumo#r OR TX malignan* OR TX oncolog* OR TX carcinom* OR TX leuk#emia OR TX lymphoma OR TX sarcoma OR MH "Precancerous Conditions + " OR TX precancer* OR TX preneoplas*) (zusammen 1118) S3 (LA German OR LA English) S4 S1 AND S2 AND S3 S5 (TI (systematic* n3 review*)) or (AB (systematic* n3 review*)) or (TI (systematic* n3 bibliographic*)) or (AB (systematic* n3 bibliographic*)) or (TI (systematic* n3 literature)) or (AB (systematic* n3 literature)) or (TI (comprehensive* n3 literature)) or (AB (comprehensive* n3 literature)) or (TI (comprehensive* n3 bibliographic*)) or (AB (comprehensive* n3 bibliographic*)) or (TI (integrative n3 review)) or (AB (integrative n3 review)) or (JN “Cochrane Database of Systematic Reviews”) or (TI (information n2 synthesis)) or (TI (data n2 synthesis)) or (AB (information n2 synthesis)) or (AB (data n2 synthesis)) or (TI (data n2 extract*)) or (AB (data n2 extract*)) or (TI (medline or pubmed or psyclit or cinahl or (psycinfo not “psycinfo database”) or “web of science” or scopus or embase)) or (AB (medline or pubmed or psyclit or cinahl or (psycinfo not “psycinfo database”) or “web of science” or scopus or embase or central)) or (MH “Systematic Review”) or (MH “Meta Analysis”) or (TI (meta-analy* or metaanaly*)) or (AB (meta-analy* or metaanaly*)) or TI (network analy*) or AB (network analy*) S6 (MH "Clinical Trials + ") or PT Clinical trial or TX clinic* n1 trial* or TX ( (singl* n1 blind*) or (singl* n1 mask*)) or TX ((doubl* n1 blind*) or (doubl* n1 mask*)) or TX ( (tripl* n1 blind*) or (tripl* n1 mask*)) or TX ((trebl* n1 blind*) or (trebl* n1 mask*)) or TX randomi* control* trial* or (MH "Random Assignment") or TX random* allocat* or TX placebo* or MH "Placebos") or MH "Quantitative Studies") or TX allocat* random* S7 S4 AND (S5 OR S6) S8 S4 NOT S7

### Assessment of risk of bias and methodological quality

All characteristics were assessed by two independent reviewers (CL and SK). In case of disagreement a third reviewer was consulted (JH) and consensus was made by discussion.

The risk of bias in the included studies was analysed with the AMSTAR-Checklist Version 2.0 for the SR [40], the SIGN-Checklist for controlled trials Version 2.0 [41], the SIGN-Checklist for cohort studies Version 3.0 [42] and the IHE-Checklist for single-arm studies and case series [43]. In addition, blinding of researchers, blinding of outcome assessment and comparability of groups before treatment, not only in terms of demographic variables but also concerning the outcomes, were examined.

The included studies were rated with the Oxford criteria. Additional criteria concerning methodology were size of population, application of power analysis, dealing with missing data and drop-out (report of drop-out reasons, application of intention-to-treat-analysis), adequacy of statistical tests (e.g. control of premises or multiple testing) and selective outcome reporting (report of all assessed outcomes with specification of statistical data as the *p* value).

### Data extraction

Data extraction was performed by one reviewer (CL) and controlled by two independent reviewers (JD, JH). As a template for data extraction, the evidence tables from the national Guideline of Complementary and Alternative Medicine in Oncological Patients of the German Guideline Program in Oncology [44] were used.

## Results

**Fig. 1:**
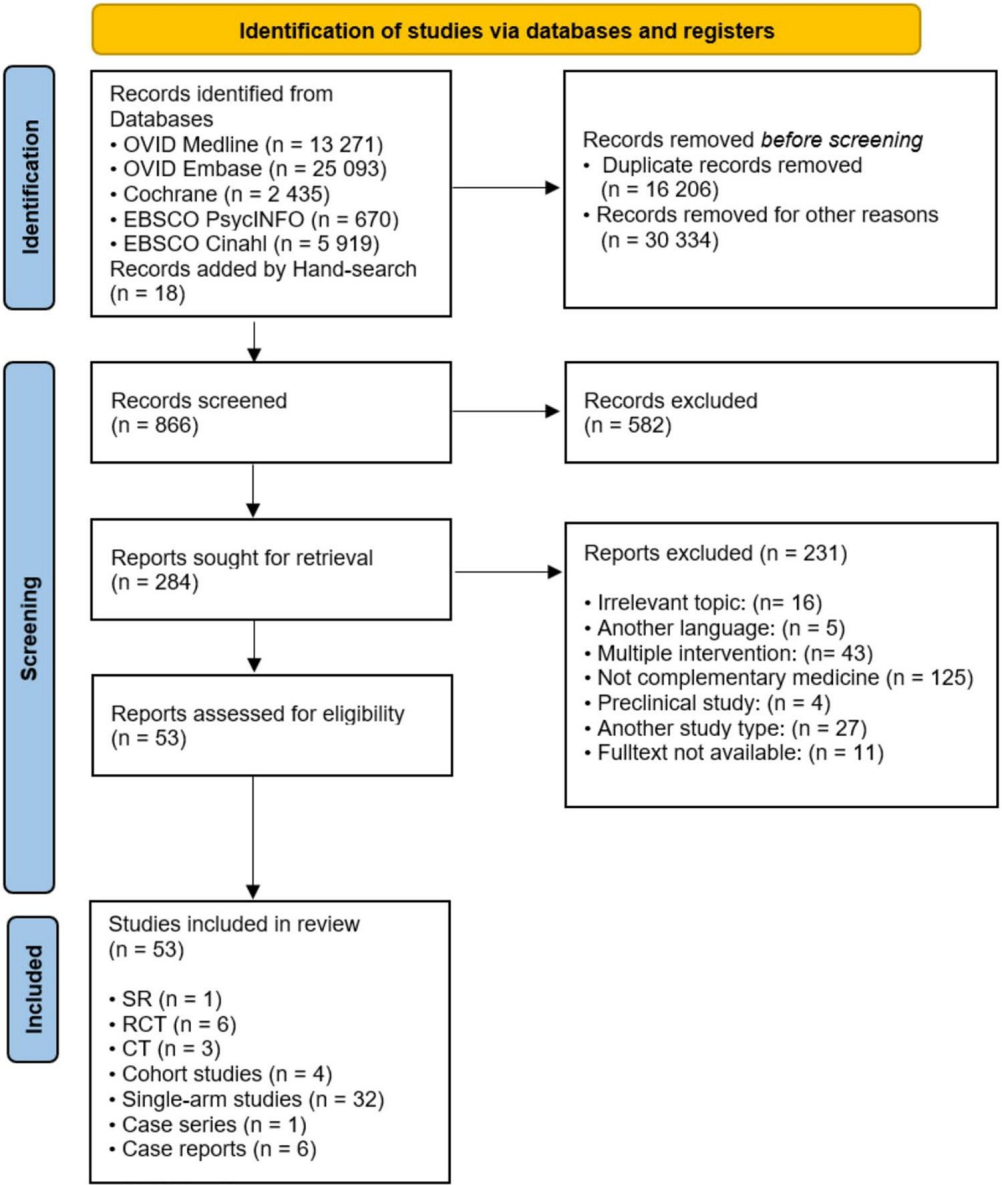
Consort diagram

The systematic research revealed 47,388 results. Eighteen studies were added by hand search. At first, duplicates were removed leaving 31,200 studies. Of these, 30,334 studies were rejected due to several reasons. After title–abstract screening 284 studies remained for full-text copy from which 231 studies were excluded due to following reasons: 16 studies did not use hyperthermia, another 125 studies did not use alternative hyperthermia, and in 43 studies, multiple interventions were administered at the same time so that an assessment of hyperthermia was not possible. Moreover, five studies were not published in English or German. For 11 abstracts, full text was not available, and four studies included only preclinical aspects. An inadequate article type (e.g. poster, letter to the editor, conference articles) was another reason for the exclusion of 27 studies. The flowchart of studies through the review can be seen in Fig. 1. A list of the studies excluded after full-text screening and the reasons for exclusion are presented in supplementary Table 3.

**Table 3:** Efficacy and side effects of hyperthermia in studies of the first level of evidence (SR, RCTs, CTs and cohort studies)

Reference	Intervention	Endpoints	Outcomes	Side effects
Lassche et al. [45]	WBH + optional other treatment modalities	1. RR (CR and PR) 2. Serious toxicity (grade 3 or 4)	1. *Over all trials*: RR: 12–89% *Recurrent or platinum resistant epithelial ovarian cancer*: WBH + CTx, RR: 38–45% (Atmaca 2009 (*n* = 35), Douwes 2004 (*n* = 21), Westermann 2001 (*n* = 12)) *Metastatic colorectal cancer*: WBH + CTx, RR: 20% and 27%. (Hegewisch-Becker 2002 (*n* = 41), Hildebrandt 2004 (*n* = 10)) *Small cell lung carcinoma*: WBH + CTx, both published in 1982, lack description of pre-treatments, RR: 86–89% (Engelhardt (*n* = 15), Neumann (*n* = 18)) *Sarcoma*: WBH + CTx, RR: 12–58%. (Bull 1992 (*n* = 17), Westermann 2003 (*n* = 95), Wiedemann 1996 (*n* = 12)) *Cervical cancer*: WBH + CTx, RR: 34% (Richel 2004 (*n* = 21)) *Melanoma*: WBH + CTx, RR: 20% (Engelhardt 1990 (*n* = 15)) *Pleural mesothelioma*: WBH + CTx, RR: 20% (Bakhshandeh-Bath 2003 (*n* = 20))	2. Serious toxicity in almost all studies (oldest studies did not report any (serious) toxicity or did not grade it). According to CTCAE: Most frequently grade 3 and 4 toxicities in studies using WBH + CTx: myelosuppression (anaemia 5–49%, leucopenia 14–100%, thrombocytopenia 5–65%) *Related to WBH*: (grade 3 and 4) Ventricular cardiac arrhythmias, dermal complications, kidney failure All studies combined: 966 cycles of WBH + CTx in *n* = 350 evaluable patients: *n* = 4 died of treatment related complications, due to infectious complications (Bakhshandeh-Bath 2003, Hegewisch-Becker 2002, Westermann 2003)
Sulyok et al. [46]	*Arm A*: WBH (Heckel HT 3000) + surgery (*n* = 9) *Arm B*: only surgery (*n* = 9) *Type of cancer*: Colorectal Cancer *Duration target temp. per session*: 39.0 °C for 2 h *Period of time*: ni	1. Quality of Recovery: (QoR)-40 questionnaire at 24 h after intervention. (Score: 40–200, higher values: better quality of recovery) 2. Immunological markers	1. No significant difference, global QoR-40 score of (mean (SD)) 167 (15) in arm A and 159 (16) in arm B. No significant differences for the individual dimensions (*p* = 0.81) 2. HSPs: increased in WBH group after treatment. HSP60 (in %) at T4: 143 (arm A) versus 89 (arm B) (*p* = 0.04). HSP90 (in %) at T2: 111 (arm A) versus 64 (arm B) (*p* = 0.04). HSP70: no significant difference (*p* = 0.40). TNF *α*-levels (in %) at T3: significant elevated in arm B. In arm A near BL: 73 (arm A) versus 151 (arm B) (*p* = 0.04). PCT at T3: increased in both groups, increase significantly higher in arm B (*p* = 0.02). No significant differences between the groups for IL-6, IL-10, HLA-DR, or LBP (*p* values for area under the level/time curve. T0: before treatment, T1: after treatment, T2: after surgery, T3: 24 h after surgery, T4: 48 h after surgery, T5: 5 days after surgery)	*Related to WBH*: (*n* = 9) Transient erythema on chest: 60% Two round, thermal lesions appeared after WBH (combustion grade 2, 1.5 cm in diameter on both lower breasts): *n* = 1 According to the authors: no sedation related side-effects in either group, and no subject reported hyperthermia related stress
Robins et al. [47]	WBH (Aquatherm) + CTx WBH alone during week 1→ randomized to receive either Melphalan alone on week 2 and Melphalan + WBH on week 5, or reverse sequence (*n* = 16) *Type of cancer*: Mixed cancer diagnosis *Duration target temp. per session*: 41.8 °C for 1 h Total of 49 WBH treatments *Period of time*: May 1992-May 1995	1. Tumour response 2. Myelosuppression 3. Nausea/vomiting 4. CTx-pharmacokinetics	1. CR: *n* = 1, PR: *n* = 2, SD: *n* = 1, PD: *n* = 10, not measurable: *n* = 1. Reduction of tumour marker: *n* = 1 2. Average (across all CTx-levels): Mean nadir WBC count: Melphalan + WBH 35% lower than Melphalan alone (p = 0.006) At 17.5 mg/m^2^ Melphalan: mean WBC nadir: Melphalan alone: 3.8 ± 0.4 10^3^/µl, Melphalan + WBH: 2.89 ± 0.8 10^3^/µl. Mean nadir platelet count: Melphalan + WBH 20% lower than for Melphalan alone (*p* = 0.04). Mean platelet count nadirs: Melphalan alone: 168 ± 24 10^3^/µl, Melphalan + WBH: 132 ± 21 10^3^/µl 3. Nausea or vomiting: controlled with antiemetics. WBH alone: 19%, Melphalan alone: 44% and WBH + Melphalan: 31% 4. WBH: no significant alteration in clearance or distribution of Melphalan. Terminal half-life slightly prolonged in combination courses	According to NCICTC (*n* = 16) *Related to WBH*: Low-grade fever (< 24 h posttreatment): *n* = 3 Oral herpes simplex (grade 1): *n* = 7 Transient increase in liver function tests (grade 2): *n* = 3 Urinary tract infection (grade 1): *n* = 1 No haematological toxicity associated with WBH alone *Related to WBH + CTx*: Myelosuppression at Melphalan: 17.5 mg (*n* = 6): Melphalan alone: thrombocytopenia (grade 1): *n* = 1, neutropenia (grade 1): *n* = 1. Melphalan + WBH: neutropenia (grade 2): *n* = 1, (grade 3): *n* = 1, (grade 4): *n* = 1, thrombocytopenia (grade 1): *n* = 1 No instances of bacterial infection, bleeding, or neutropenic fevers. All patients: recovery of blood counts after completion of therapy
Hegewisch-Becker et al. [48]	All patients in weekly changing regime: CTx + WBH (Enthermics, RHS 7500) *(= arm A),* CTx without WBH *(= arm B)* (n = 44) *Type of cancer:* Adenocarcinoma of colon or rectum *Duration target temp. per session:* 41.8 °C for 60 min *Overall number of cycles:* 273 (130 with and 143 without WBH) *Period of time:* January 1999- January 2001	1. Tumour-response 2. TTP and OS from beginning CTx-treatment until disease progression or death 3. 1y OS (Kaplan–Meier -estimated probability)	1. CR: n = 2, PR: n = 6, SD: n = 23, PD: n = 9, Death: n = 1, due to sepsis and tumour lysis. RR: (CR and PR): n = 8 (20%), (95% CI: 9–35%) **2.** Median TTP, from begin of treatment: 21 weeks (95% CI: 17–25 weeks). Median OS, from begin of treatment: 50 weeks (95% CI: 39–61 weeks) 3. 46%	*According to WHO (n = 44)* *Related to WBH:* Mucosal herpes infections: *n* = 17, responsive to acyclovir Pressure scores (grade 1 and 2): *n* = 3 Transient cardiac arrhythmias with ECG signs of myocardial ischaemia (grade 3): *n* = 5 Comparison Group A and B: *Haematological toxicity*: In cycles with WBH: grade 0: 91.7%, grade 1: 2.6%, grade 2: 4.4%, grade 3: 1.2% In cycles without WBH: grade 0: 93.7%, grade 1: 1.6%, grade 2: 2.8%, grade 3: 1.9% *Gastrointestinal toxicity*: In cycles with WBH: grade 0: 82.6%, grade 1: 11%, grade 2: 4.9%, grade 3: 1.5% In cycles without WBH: grade 0: 85.5%, grade 1: 9.6%, grade 2: 3.7%, grade 3: 1.2% *Peripheral neurotoxicity*: In cycle with WBH: grade 0: 39.2%, grade 1: 40.8%, grade 2: 18.5%, grade 3: 1.5% In cycle without WBH: grade 0: 49.0%, grade 1: 35.0%, grade 2: 14.7%, grade 3: 1.4% *Fatigue syndrome*: In cycle with WBH: grade 3: 20%, grade 4: 5% In cycle without WBH: grade 3: 6%, grade 4: 3% Related to Oxaliplatin: Most frequent: mild neurosensory toxicities: 68% Severe sensory neuropathy with functional impairment due to loss of sensitivity in fingertips and soles of feet towards end of therapy: *n* = 1 Almost all patients reported neurotoxicity to be less pronounced in cycles combined with WBH
Gerke et al. [49]	*Arm A*: ECC-WBH (Level One) + CTx (n = 9) *Arm B:* rWBH (Aquatherm) + CTx (n = 18) *Arm C:* CTx alone (n = 16) *Type of cancer:* Sarcoma *Duration target temp. per session:* 41.8 °C for 1 h *Number of treatments:* 4–6 courses *Period of time:* January–December 1995	1. Serum creatinine, GFR, marker proteins (albumin, IgG, *α*1-microblobulin): comparison between three groups at T3 2. Serum creatinine, GFR, marker proteins: comparison between three groups at T4 (T0:1 day before ICE, T3: day 3 of ICE, T4: 21 days after T0)	1. GFR: decreased more profoundly in the WBH treated patients than in patients treated with CTx alone (ICE vs. ICE + ECC-WBH: *p* = 0.016, ICE versus ICE + rWBH: *p* = 0.037) between ECC-WBH and rWBH no significant difference (*p* = 0.364) *Creatinine*: no significant difference between WBH + ICE groups and sole ICE group (ICE vs. ICE + ECC-WBH: *p* = 0.111, ICE versus ICE + rWBH: *p* = 0.227), no significant difference between ECC-WBH und rWBH (*p* = 0.364) *Marker-Proteins*: increased significantly more profoundly in WBH-treated patients than in patients treated with CTx alone (*p* < 0.05). Increase not significant different between ECC-WBH and rWBH (*p* > 0.05) 2. *GFR*: no significant difference between WBH + ICE group and sole ICE group (ICE vs. ICE + ECC-WBH: *p* = 0.631, ICE versaus ICE + rWBH: *p* = 0.763), no significant difference between ECC-WBH und rWBH (*p* = 0.688) *Creatinine*: no significant difference between WBH + ICE group and sole ICE group (ICE vs. ICE + ECC-WBH: *p* = 0.873, ICE versus ICE + rWBH: *p* = 0.921), no significant difference between ECC-WBH und rWBH (*p* = 0.841) *Marker-proteins*: no significant difference between ICE and ICE + rWBH. In ECC-WBH group: all 3 marker-proteins significantly pathological elevated in comparison with T0 (*p* < 0.05). Intergroup-comparison: ICE alone versus ICE + ECC-WBH and ICE + ECC-WBH versus ICE + rWBH: significant higher albumin and *α*1-microglobulin-values in ICE + ECC-WBH group. (*p* < 0.05)	Except of analysed nephrotoxicity no further information
Reuter et al. [50]	WBH (Iratherm 1000) + induced therapeutic fever *Group A1:* bacterial extracts: Se + Stp and Ps + Stp without preceding hyperthermia (135 applications, *n* = 44) *Group A2:* bacterial extracts (Se + Stp and Ps + Stp) preceded by 30 min WBH (215 applications, *n* = 62) *Group B:* combinations of approved drugs (Colibiogen, Iscador, Picibanil, Polyvaccinum, Strovac) preceded by WBH (100 applications, *n* = 25) *Type of cancer:* Mixed cancer diagnosis and other diseases *Duration target temp. per session:* 39–40 °C, no further description *Period of time:* ni	1. Effect of preceding WBH	1. Side reactions and difference between Se and Ps drastically reduced	*Related to combination therapy (no further distinction):* Nausea/vomiting: group A1: Ps 15%, Se 24.9%, group A2: Ps 6.1%, Se 8.2%, group B: 26% Headache: group A1: Ps 12%, Se 19.3%, group A2: Ps 5.5%, Se 6.1% group B: 25% Back pain: group A1: Ps 5.4%, Se 7.4%, group A2: Ps 2.4%, Se 2.3%, group B: 12% Circulatory reactions: group A1: Ps 7.7%, Se 10.9%, group A2: Ps 3.1%, Se 3.2%, group B: 0.5% Weakness next day: group A1: Ps 17.7%, Se 21.2%, group A2: Ps 13.1%, Se 16.1%, group B: 0.5%
Minnaar et al. [51]	*Arm A:* EH (EHY2000 + , Oncotherm, 2 treatments per week) + CTx + RTx (*n* = 104) *Arm B*: (CTx + RTx) (*n* = 106) *Duration target temp. per session:* 42.5 °C for a minimum of 55 min *Treatment duration*: ni, planned 10 treatments *Period of time*: January 2014–August 2018	1. 6-month LDFS (local disease-free survival) arm A: *n* = 101, arm B: *n* = 101 2. LDC (local disease control) censored for survival (arm A: *n* = 88, arm B: *n* = 83) 3. Tumour response (arm A: *n* = 85, arm B: *n* = 73)	1. Arm A: *n* = 39 (38.6%), arm B: *n* = 20 (19.8%) (*p* = 0.003) 2. Arm A: *n* = 40 (45%), arm B: *n* = 20 (24%) (*p* = 0.003) 3. CMR (complete metabolic response): arm A: *n* = 49 (57.6%), arm B: *n* = 26 (35.6%) (*p* = 0.005) PMR (partial metabolic response): arm A: *n* = 33 (38.8%), arm B *n* = 44 (60.3%), SMD (stable metabolic disease): arm A: *n* = 1 (1.2%), arm B: *n* = 3 (4.1%), PMD (progressed metabolic response): arm A: *n* = 2 (2.4%), arm B: *n* = 0	*Related to EH (according to CTCAE, n = 104):* 91.4% participants received ≥ 8 of planned 10 EH treatments. Reasons for not receiving ≥ 8 EH: adipose burns (*n* = 2), 1 cm blister (*n* = 1), progressed on treatment (*n* = 1), moist desquamation resulting in RT and EH delay (*n* = 2), bleeding resulting in RTx and EH delay (*n* = 1), did not arrive for RTx or EH (*n* = 1), deceased on treatment (*n* = 1) Adipose tissue burns (grade 1–2): *n* = 10, (9.5%) Surface burns (grade 1): *n* = 2 (2.0%) Pain: *n* = 9 (8.6%)
Minnaar et al. [52]	Same study as Minnaar (2019), other endpoint	1. QoL: C30 and Cx24 (EORTC) T1: 6 weeks post-treatment, T2: 3 months post-treatment	1. At BL: no statistically significant differences in QLQ scores between two arms T1: mean change in cognitive function: arm A significantly higher than arm B (*p* = 0.031) T2: arm A compared to arm B: significant improvement in social functioning (*p* = 0.049), emotional functioning (*p* = 0.017), fatigue (*p* = 0.037) and pain (*p* = 0.007) Mean improvement in social, emotional and physical function at T1 significantly higher in patients with CR	*Others related to EH:* HIV status, increased BMI and average energy not significant predictors of adverse events associated with EH
Minnaar et al. [53]	Same study as Minnaar (2019), other endpoint	1. Abscopal response: all disease (primary tumour, lymph nodes within and outside radiation field) showing a CMR (complete metabolic response) at T1 in 18-FDG PET/CT (fluorodeoxyglucose-positron-emission tomography)	1. Higher in arm A *n* = 13 (24.1%) compared to arm B: *n* = 3 (5.6%) (*p* = 0.013)	
Loboda et al. [54]	*Arm A:* EH (MagTherm system Radmir, Ukraine, 27.12 ± 0.16 MHz) + neoadjuvant CTx (n = 103) *Arm B:* only neoadjuvant CTx (n = 97) *Type of cancer:* Locally advanced breast cancer *Duration target temp. per session:* 37–38.8 °C for 30 min *Treatment duration: ni* *Period of time:* 2008–2017	1. Blood flow of the breast 2. Tumour response 3. Survival	1. Arm A: blood flow increased from 44.58 cm/s to 192.78 cm/s after EH. Mean values for systolic blood flow 3.5 times as high as those prior to EH, mean diastolic blood flow raised after EH 2. CR: arm A: *n* = 9, arm B: *n* = 6 (*p* = 0.68) PR: arm A: *n* = 51, arm B: *n* = 35 (*p* = 0.076) SD: arm A: *n* = 37, arm B: *n* = 49 (*p* = 0.052) PD: arm A: *n* = 6, arm B: *n* = 7 (*p* = 0.91) 3. 10-year OS significantly higher in arm A (*p* < 0.009)	*Comparison arm A to arm B:* Haematologic, gastrointestinal toxicities, liver, and kidney function: no significant difference
Mahdavi et al. [55]	*Arm A:* EH (Celsius 42 +) + CTx + RTx, n = 19 *Arm B:* CTx + RTx, n = 19 *Type of cancer:* Glioblastoma *Duration target temp. per session:* About 41 °C for 1 h, 2 times a week *Number of treatments:* 10–12 courses *Period of time:* ni	1. OS, after 18 months (means ± SD) 2. Karnofsky Performance Status Scale (KPS), T0: at BL, T1: after treatment, T2: after 3 months (mean values) 3. Tumor volumes, measured by MRI (mean ± SD, in cm^3^), T0: at BL, T1: 3 months after treatment, T2: 6 months after treatment	1. Arm A: 15.47 ± 4.6 months, arm B: 14.57 ± 4.5 months, no significant difference between arms (p = 0.55) 2. Arm A: T0: 86.31, T1: 88.95, T2: 85.26, arm B: T0: 84.73, T1: 84.21, T2: 78.94 no significant differences between T0–T3 3. Arm A: T0: 104.14 ± 58.4, arm B: T0: 135.42 ± 92.5, difference not statistically significant (*p* = 0.2) arm A: T1: 68.08 ± 59.64, T2: 68.41 ± 62.14 arm B: T1: 137.63 ± 113.93, T2: 151.42 ± 117.10, difference statistically significant (*p* < 0.05)	*Related to EH: (based on questionnaire):* Mild headache, no necessity for any additional medication
Fiorentini et al. [56]	*Type of cancer* *n* = 111 glioblastoma multiforme (GBM), n = 38 astrocytoma (AST) *Arm A:* *n* = 52 (*n* = 28: GBM, *n* = 24: AST): EH (EHY-2000 + , Oncotherm, 13.56 MHz), in arm A no CTx *Arm B:* *n* = 97 (*n* = 83 GBM, *n* = 14 AST): BSC (best supportive care: dexamethasone, glycerol, mannitol, holistic therapy, psychosocial support) In arm B: *n* = 28 (all GBM) received additionally CTx *Duration target temp. per session:* 40–42.5 °C for 20–60 min *Treatment duration:* 3 times per week for 8 weeks. Median number of EH treatments/patient: 22 (range: 11–62) *Period of time:* April 2003–January 2018	1. Tumor response at 3 months 2. Median OS 3. Quality of life	**1.** Arm A: AST patients: CR: *n* = 2 (9%), PR: *n* = 8 (36%), SD: *n* = 6 (27%) Overall positive response of AST (CR + PR + SD): arm A: 72%, significantly higher than in arm B: 37% (*p* < 0.005) PD: arm A: *n* = 4 (18%) patients, arm B: *n* = 9 (56%) Arm A: GBM patients: CR: *n* = 1 (4%), PR: *n* = 6 (21%), SD: *n* = 8 (29%), Overall positive response of GBM (CR + PR + SD): arm A: 54% significantly higher than in arm B: 19% (*p* < 0.05) PD: arm A: *n* = 13 (46%), arm B: *n* = 62 (75%) 2. Median OS AST patients: arm A: 16 months (range: 3–156), arm B: 16.5 months (range: 3–120 months) (*p* = 0.0065) 5-year OS AST patients: arm A: 83%, arm B: 25% Median OS GBM patients: arm A: 14 months (range: 2–108 months), arm B: 9 months (range: 2–84 months), (*p* = 0.047) 3. Most patients reported better QoL (evaluated by subjective responses as during follow-up visits) in arm A	*Related to EH: (according to CTCAE, n = 50)* Headache: *n* = 1 (2%) Scalp burn *n* = 1 (2%) Seizures *n* = 5 (10%) (all patients experienced this symptom from the beginning of disease. Seizure during EH in *n* = 1)
Kim et al. [57]	*Arm A:* EH (EHY2000, Oncotherm) + conventional cancer treatment (after PSM: *n* = 35, at BL: *n* = 32) *Arm B:* Conventional cancer treatment alone (after PSM: *n* = 175, at BL: *n* = 83) *Type of cancer:* Lung cancer *Duration target temp. per session:* 39–42 °C for 60 min *Numbers of treatment sessions:* 1–47 (mean: 19.3) *Treatment durations:* 1–42 weeks (mean: 10.3 weeks) *Period of time:* 2010–2013	1. Pain intensity (PI): (numeric scale: 0–10) 2. Opioid analgesic dose (M) 3. Effective analgesic score (EAS): PI [1 + (M/10)]: increase in EAS: indication for problem with adequate analgesia 4. EAS-changes over time (T0: BL: days -30–0, T1: days 1–60, T2: days 61–120, T3: days 121–180)	1. No significant differences between arms at any time 2. Significant higher M in arm A at T1 (means ± SD): in arm A: 479.29 mg ± 685.01 mg, in arm B: 243.60 mg ± 403.06 mg (p = 0.022) 3. No significant differences between arms at any time 4. EAS: significant interaction treatment x time: p = 0.038, significant interaction with T1, with higher (so worse) values for arm A compared to arm B. SMD: 101.76 points, standard error: 46.22 points, 95% CI: 10.20–193.32 points (*p* = 0.03)	*Related to EH:* Pain due to EH particularly during early stages of treatment
Kim et al. [58]	*Arm A:* EH (EHY2000, Oncotherm) additional during neoadjuvant RTx and CTx (*n* = 62) *Arm B:* only neoadjuvant RTx and CTx (*n* = 58) 6–8 weeks after neoadjuvant treatment: surgery *Type of cancer:* Locally advanced rectal cancer *Duration target temp. per session:* 60 min, n = 59 more than 8 session, temp: ni *Treatment durations: ni* *Period of time:* May 2012–December 2017	1. Pathologic outcome (pathologic T stage, T-downstaging rate pathologic N stage, N-downstaging rate, downstaging rate, TRG (tumor regression grade)) 2. Survival (*n* = 113 of 120)	1. No significant differences between arms. (except: TRG 3 (near total regression) + TRG 4 (total regression) only for tumours with initial primary tumor volume > 65 ml: arm A: *n* = 6 (31.6%), arm B: *n* = 0 (0%) (*p* = 0.024) 2. Median follow-up period: arm A: 45 months (range: 7–71 months), arm B: 58 months (range: 6–95 months) 2-year OS: arm A: 100%, arm B: 96% no significant difference 2-year DFS: arm A: 96%, arm B: 76% (*p* = 0.054) 2-year LRRFS (locoregional recurrence free survival): arm A: 98%, arm B: 94% (*p* = 0.09) 2-year DMFS (distant metastases free survival): arm A: 94%, arm B: 79% (*p* = 0.083)	*Related to EH (according to Berlin scoring system, n = 62):* Hot spot: *n* = 1 Fat necrosis: *n* = 1 Heat-related (no further information) grade 0: *n* = 45, grade 1: *n* = 15, grade 2: *n* = 0, grade 3: *n* = 2 *Comparison arm A to arm B (according to NCICTC, n = 120):* Incidence of leucopenia, neutropenia, and genitourinary toxicities similar between the two arms. (leucopenia: *p* = 0.219, neutropenia: *p* = 0.802, genitourinary: *p* = 0.362) Gastrointestinal toxicity: arm A: 64.5% (grade 0: *n* = 22, grade 1: *n* = 21, grade 3: *n* = 19), arm B: 87.9% (grade 0: *n* = 7, grade 1: *n* = 25, grade 2: *n* = 26) (p = 0.01)

*1y-OS* 1 year-overall-survival; *AST* astrocytoma; *BL* baseline; *BMI* body mass index; *CI* confidence Interval; *CR* complete response; *CTx* chemotherapy; *EAS* effective analgesic score; *EAS* (PI [1 + (M/10)]: 1: anti-inflammatory drug at a regular dosage; *M* the weekly dose (mg) in oral morphine equivalents; *n* number of patients; *PI* the pain intensity on a 1–10 scale); *ECC-WBH* extracorporeal-circulation-WBH; *ECG* electrocardiographic; *EH* electro hyperthermia; *GBM* glioblastoma multiforme; *GFR* glomerular filtration rate; *HLA-DR* human leucocyte antigen of class DR; *HSP* heat-shock protein; *ICE* CTx of Ifosfamide, carboplatin and etoposide; *IL* interleukin; *LBP* lipopolysaccharide binding protein; *MV* mean value; *NCICTC* NCI common terminology criteria for adverse events; *ni* no information; *OS* overall survival; *PCT* procalcitonin; *PI* pain intensity; *PR* partial response; *Ps* Pseudomonas aeruginosa; *PSM* propensity score matching; *QoR* quality of recovery; *RR* response rate; *rWBH* radiant heat induced WBH; *Se* Serratia marcescens; *SD* stable disease; *SMD* standardized mean deviation; *ST* survival time; *Stp* Streptococcus pyogenes; *TNF-α* tumour-necrosis factor-α; *TRG* tumour regression grade; *TTP* time to progression; *vs* versus; *WBH* whole-body-hyperthermia; *WHO*: World-Health-Organization; *WBC* white blood cells

**Fig. 2:**
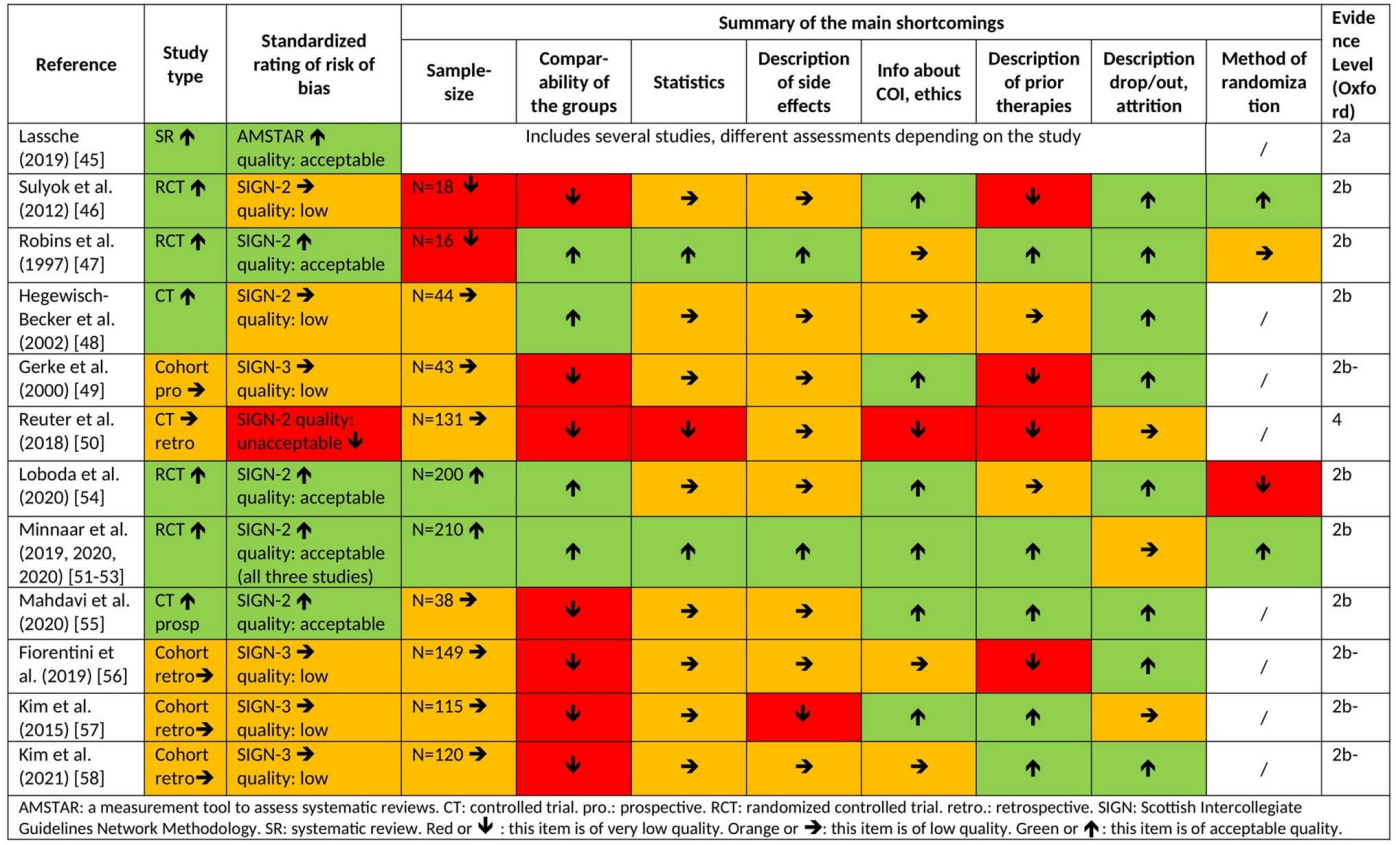
Risk of bias of studies of the first level of evidence (short characteristics)

**Fig. 3:**
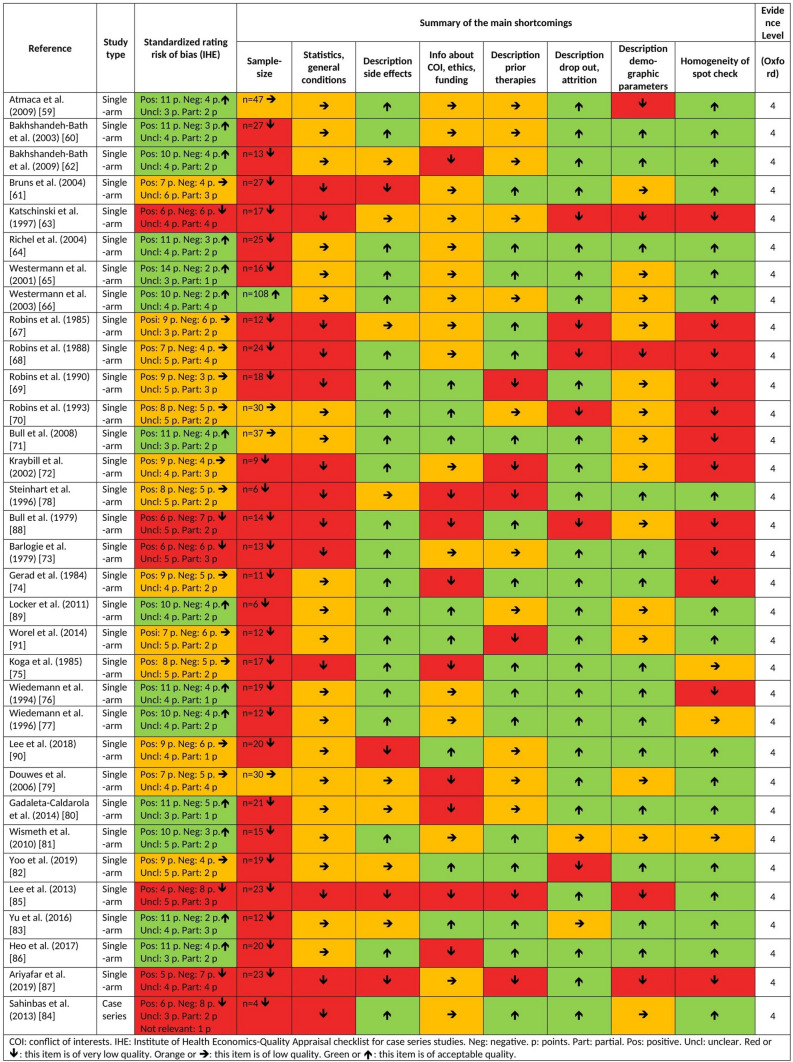
Risk of bias of studies of the second level of evidence (short characteristics)

Finally, 96 publications were analysed in this review: 53 studies on alternative hyperthermia and 43 studies including multiple interventions which were only considered with respect to side effects of hyperthermia. Detailed characterization of the included studies may be seen in Figs. 2 and 3 and in Tables 3, 4, 5, and 6. In the 43 studies with multiple interventions, only the side effects were analysed. The characteristics of these studies can be seen in Table 7, the relevant adverse events in Table 8.

**Table 4:** Efficacy of hyperthermia in studies of the second level of evidence (single-arm studies and case series)

Reference		Intervention, Duration of target temp. per session, Period of time of the study	Number of participants, Cancer type	Outcome
*Outcome: Tumour response*				
Atmaca et al. [59]		WBH (Aquatherm) + CTx, 41.8 °C for 1 h. Cycles repeated every 28 days up to a total of 6 cycles. Median cycles: 2.7 (range: 1–6). Period of time: May 1997-December 2002	*n* = 35. Ovarian carcinoma	CR: 11%, PR: 34%, SD: 26%, PD: 29%, RR (PR and CR): 45.7%
Bakhshandeh-Bath et al. [60] Bruns et al. [61]		WBH (Aquatherm) + CTx, 41.8 °C for 1 h. Responding patients up to 2 additional cycles. Period of time: April 1999-February 2001	*n* = 25. Pleural mesothelioma	Overall RR: 20% (95% CI 8.9–39.1%), CR: *n* = 0, PR: *n* = 5, MR: *n* = 3, SD: *n* = 11, PD: *n* = 6
Bakhshandeh-Bath et al. [62]		WBH (Aquatherm) + CTx, 41.8 °C for 1 h. Patients received second cycle 4 weeks after first cycle. Period of time: March 2000-March 2003	*n* = 13. Pancreatic adenocarcinoma	PR: *n* = 3, SD: *n* = 5, RR (PR and CR): 23%
Katschinski et al. [63]		WBH (Aquatherm) + CTx, 41.8 °C for 60 min. Total of 53 cycles. Period of time: ni	*n* = 17. Mixed cancer diagnosis	Comparison RR early versus late CTx-schedule: no significant difference (62 ± 6%)
Richel et al. [64]		WBH (Aquatherm) + CTx, 41.8 °C for 1 h. Total of 82 courses, median of 3 courses per patient (range: 1–6). Period of time: ni	*n* = 21. Cervical cancer	CR: *n* = 1, PR: *n* = 6, RR (PR and CR): 33% (95%-CI: 13–53%), SD: *n* = 9, PD: *n* = 5
Westermann et al. [65]		WBH (Aquatherm) + CTx, 41.8 °C for 1 h. Total of 44 combination treatments. Period of time: 1995–1999	*n* = 12. Ovarian carcinoma	CR: *n* = 1, PR: *n* = 4, SD: *n* = 4, PD: *n* = 3, RR (CR and PR): 35.7% (90%-CI: 15.3–60.9%)
Westermann et al. [66]		WBH (Aquatherm) + CTx, 41.8 °C for 1 h. Responding patients up to 2 additional cycles. Period of time: May 1995–December 2000	*n* = 95. Soft tissue sarcoma	CR: *n* = 4, PR: *n* = 23, SD: *n* = 31, PD: *n* = 37. RR (PR and CR, overall): 28.4% (95% CI: 19.8–38.5%). RR (no prior therapy): 36%, RR (pretreated patients): 24%. → Difference (RR pretreated vs. no prior therapy): not significant (*p* = 0.238)
Robins et al. [67]		WBH (Enthermics) alone, 39.5–41.8 °C for 35–140 min. Number of treatments at each level: 3–7. Total of 52 treatments. Period of time: ni	*n* = 10. Mixed cancer diagnosis	SD: *n* = 5 (Median: 5 months), MR: *n* = 3, RR (PR and CR): 0%
Robins et al. [68]		WBH (Enthermics) + CTx, total of WBH-treatments: at 41.0 °C for 85 min: *n* (treatments) = 93, at 41.8 °C for 75 min: *n* = 105. Repetition due to escalation temperature scheme. Period of time: ni	*n* = 23. Mixed cancer diagnosis	Group A: no response: *n* = 3. Group B: PR: *n* = 2, improvement (less than PR): *n* = 1. Group C: CR: *n* = 1, improvement: *n* = 2, SD: *n* = 3, PD: *n* = 11. RR (overall PR and CR): 13% (Groups: different concentration of CTx)
Robins et al. [69]		WBH (Enthermics) + RTx, 41.8 °C for 75 min. Total of 97 WBH-treatments. Period of time: November 1983–April 1987	*n* = 8. B-cell neoplasms	CR: *n* = 3 (*n* = 2 remain in a CR), PR: *n* = 4, improvement (a 48% decrease in tumour burden): *n* = 1, RR (PR and CR): 87.5%
Robins et al. [70]		WBH (Enthermics) + CTx, 41.8 ± 0.2 °C for 60 min WBH alone in week 1, WBH + CTx in week 2, CTx alone in week 5. Responding patients: WBH + CTx to maximum of further 5 cycles. Period of time: ni	*n* = 30. Mixed cancer diagnosis	CR: *n* = 1 (neuroendocrine tumour → hormone marker-negative, TTP: 410 days), PR: *n* = 2 (TTP: 96 days, 208 days), SCR: *n* = 2 (TTP: > 9 months, 233 days), MR: *n* = 1 (TTP: 143 days), SD: *n* = 8, improvement after WBH + CTx, but progression after CTx alone: *n* = 2, RR (PR and CR): 10%
Bull et al. [71]		WBH (Heckel HT-2000) + CTx, 40 °C for 6 h. Cycle repeated up to 7 times. (range: 1–8). Period of time: January 2000–June 2004	*n* = 37. Mixed cancer diagnosis	CR: 3%, PR: 41%, SD: 19%, PD: 38%, RR: (CR + PR): 43%
Kraybill et al. [72]		WBH (Heckel, HT-2000) alone, group A: 39–39.5 °C for 3 h, Group B: 39–39.5 °C for 6 h, Group C: 39.5–40 °C for 6 h. Period of time: ni	*n* = 9. Mixed cancer diagnosis	No clinical responses (anti-tumour effects of WBH)
Barlogie et al. [73]		WBH (water blankets, Cincinnati Sub-Zero) alone or WBH + CTx, 42 °C for 4 h. Frequency: Ø 3x. Period of time: June 1977–April 1978	*n* = 11. Mixed cancer diagnosis	CR: *n* = 0, PR: *n* = 0, SD: *n* = 7 (of them: *n* = 4 objective regression, less then PR, all apparent after WBH alone), progression: *n* = 4, RR (PR and CR): 0%
Gerad et al. [74]		WBH (nylon and vinyl mesh water perfused suit (Whittaker General Medical), heating blankets) + CTx, 41.8 °C–43.0 °C for 2 h, Total of 35 treatments, Period of time: ni	n = 11. Soft tissue Sarcoma	RR (CR and PR): 36%, CR: *n* = 2, PR: *n* = 2. RR for soft tissue sarcoma (excluding patients with mesothelioma): 44% (90% CI: 17–71%)
Koga et al. [75]		ECC-WBH (Parks and Smith) + CTx, 41.5 °C for 3–5 h, 4 times at intervals of 7–10 days (some patients received treatment only once). Period of time: ni	*n* = 17. Gastro-intestinal cancer	PR: *n* = 3, SD: *n* = 9, ST: not markedly prolonged (even in patients with PR). Not evaluable: *n* = 4 (died, probably ascribable to ECC-WBH). RR (PR and CR): 18%
Wiedemann et al. [76]		ECC-WBH (Parks and Smith) + CTx, 41.8 °C for 1 h. Patients received 3 thermo-chemotherapy treatments every 3 weeks. Total of 49 treatments. Period of time: ni	*n* = 19. Sarcoma or malignant teratoma	PR: *n* = 7 (progression 5 months after therapy: *n* = 2), SD: *n* = 8, PD: *n* = 4, RR (PR and CR): 37%
Wiedemann et al. [77]		ECC-WBH (Level One) + CTx, 41.8 °C: 1 h. Period of time: ni	*n* = 12. Sarcoma	CR: *n* = 0, PR: *n* = 7, SD: *n* = 3, PD: *n* = 2, RR (CR and PR): 58% (95%-CI: 28–85%.)
Steinhart et al. [78]		ECC-WBH (heated air blanket, Cincinnati Sub-Zero hyper-hypothermia machine) alone, 40 °C or 42 °C for 1 h. Period of time: ni	*n* = 6. Kaposi’s sarcoma	Some improvement of KS lesions (lightening in colour and decrease in size): *n* = 6, KS-lesions regressed to pre-treatment status 2-weeks post-WBH: *n* = 5, size of KS-lesion continued to diminish: *n* = 1, progression of KS: *n* = 2
Douwes et al. [79]		EH (Oncotherm EHY2000) + CTx, 60 min, temp. reached in tumour: 42–44 °C (measured non-invasive by energy absorption). Treatments repeated every 4 weeks until PD Number of treatments: mean: 3 (range: 1–9). Period of time: ni	*n* = 30. Pancreas carcinoma	CR: *n* = 1, PR: *n* = 10, SD: *n* = 12, PD: *n* = 7, DCR (CR, PR, SD): *n* = 23 (77%), RR (PR and CR): 37%
Gadaleta-Caldarola et al. [80]		EH (Oncotherm EHY2000) + Sorafenib, 60 min. 3 times/week for 6 weeks, followed by 2 weeks without treatment. Period of time: February 2009–September 2010	*n* = 21. Hepatocellular carcinoma	CR: *n* = 0, PR: *n* = 1, SD: *n* = 11, PD: *n* = 9, DCR (= CR, PR, SD): 45%, RR (PR and CR): 5%
Wismeth et al. [81]		EH (Oncotherm EHY2000) + CTx, 20–60 min. Median number of EH-sessions: 20 (range: 11–77). Period of time: January 2006–March 2008	*n* = 15, 20 lesions. Glioma WHO grade III or IV	CR: *n* = 2 lesions, PR: *n* = 1 lesion, PD: *n* = 9 lesions, SD: *n* = 5 lesions, not evaluable: *n* = 3 lesions, RR (PR and CR of the lesions): 15%
Yoo et al. [82]		EH (Oncotherm, EHY2000 + , Oncotherm), 2 sessions per week for 3 weeks, CTx before study. Temp: ni. Period of time: October 2008–March 2016	*n* = 19. Recurrent and progressive ovarian cancer	SD: *n* = 1. *N* = 18 died with a median follow-up of 8.0 months (range 2–32 months). Time to death ranged from 2.5 to 32.0 months
Yu et al. [83]		EH (Celsius42 +) + RTx, 60 min, Skin surface temp.: 36–37.5 °C, twice a week, at intervals of at least 72 h, for 5 total sessions. Period of time: November 2013–August 2014	*n* = 10. Colorectal cancer, hepatic metastasis	Metastasis response: PD: *n* = 2, PR: *n* = 3, at 2 months: hepatic PD: *n* = 3, PD-free 3 months after treatment: *n* = 3, RR (PR and CR): 30%
Sahinbas et al. [84]		EH (local electrohyperthermia) + CTx, 1 h, during first and second CTx-cycle three times a week. From third CTx-cycle two times a week. Mean: 2.25 cycles of CTx and hyperthermia. Period of time: ni	*n* = 4. Colorectal cancer, hepatic metastases	PR: *n* = 1, SD: *n* = 2, PD: *n* = 1, RR (PR and CR): 25%
*Outcome: Survival data*				
Atmaca et al. [59]		WBH (Aquatherm) + CTx, 41.8 °C for 1 h. Cycles repeated every 28 days up to a total of 6 cycles. Median: 2.7 (range: 1–6). Period of time: May 1997–December 2002	*n* = 35. Ovarian carcinoma	Median OS: 61.5 weeks (= 14.2 months, from start of treatment) (range: 5–292 weeks). Median TTP: 29 weeks (= 6.7 months, from start of treatment) (range: 14–172). Median response duration: 25 weeks (range: 9–112)
Bakhshandeh-Bath et al. [60] Bruns et al. [61]		WBH (Aquatherm) + CTx, 41.8 °C for 1 h. Responding up to 2 additional cycles. Period of time: April 1999–February 2001	*n* = 27. Pleural mesothelioma	Median ST: 76.6 weeks (= 17.6 months, from start of treatment) (95%-CI: 65–87.8 weeks). Median ST: 83.8 weeks (= 19.3 months, from diagnosis) (95% CI 73.9–93.8 weeks). PFS: 29.6 weeks (= 6.8 months, from start of treatment) (95%-CI: 24.4–34.7 weeks). 1y OS: 68%, 2y OS: 20%
Bahkshandeh-Bath et al. [62]		WBH (Aquatherm) + CTx, 41.8 °C for 1 h. Patients received second cycle 4 weeks after first cycle. Period of time: March 2000–March 2003	*n* = 13. Pancreatic adenocarcinoma	Median PFS (all patients): 4.7 months. Median OS (all patients): 11.4 months. Median OS (patients with PR): 15.8 months. 1y OS (all patients): 38%. No information, if data from start of study or from diagnosis
Richel et al. [64]		WBH (Aquatherm) + CTx, 41.8 °C for 1 h. Total of 82 courses, median of 3 per patient (range: 1–6). Period of time: ni	*n* = 21. Cervical cancer	Median PFS: 5.3 months (range: 0.5–43 + , from start of study). Median OS: 7.8 months (range: 1.3–43 + , from start of study)
Westermann et al. [66]		WBH (Aquatherm) + CTx, 41.8 °C for 1 h. Responding patients up to 2 additional cycles. Period of time: May 1995–December 2000	*n* = 95. Soft tissue sarcoma	Median OS: 327 days (= 10.7 months) (95%-CI: 393–496 days). Median TTF: 123 days (95%-CI: 77–164). Difference in OS, TTF depending on tumour response. OS: responders versus PD: significant (*p* = 0.04). OS: SD versus PD: significant (*p* = 0.07). TTF: responder versus SD: not significant (*p* = 0.31). No information, if data from start of study or from diagnosis
Robins et al. [69]		WBH (Enthermics) + RTx, 41.8 °C for 75 min. Period of time: ni	*n* = 8. B-cell neoplasms	Median ST: 52.5 months. Median TTF: 9.4 months (90%-CI: 7–15.4 months). No information, if data from start of study or from diagnosis
Bull et al. [71]		WBH (Heckel HT-2000) + CTx, 40 °C for 6 h. Cycle repeated up to 7 times. (range: 1–8). Period of time: January 2000–June 2004	*n* = 37. Mixed cancer diagnosis	Mean time to disease progression: 5.5 months. Mean OS: 8.1 months. No information, if data from start of study or from diagnosis
Wismeth et al. [81]		EH (Oncotherm EHY2000) + CTx, 20–60 min. Median number of EHT sessions: 20 (range: 11–77). Period of time: January 2006–March 2008	*n* = 15, 20 lesions. Glioma WHO, grade III or IV	Median TTP: 14 weeks (= 3.2 months) (range: 6–40). Median OS (after start of study, patients diseased at time of study report): 26 weeks (= 5.9 months) (range 14–41). Median OS (after start of study, in total population): 30 weeks (= 6.9 months) (range 14–109 weeks). Median OS (from diagnosis, in patients diseased at time of study report): 59 weeks (range 43–106). Median OS (from diagnosis, in total population): 81 weeks (= 18.6 months) (range: 43–387 weeks)
Douwes et al. [79]		EH (Oncotherm EHY2000) + CTx, 60 min, temp. reached in tumour: 42–44 °C (measured non-invasive by energy absorption). Treatments repeated every 4 weeks until PD Number of treatments: mean: 3 (range: 1–9). Period of time: ni	*n* = 30. Pancreas carcinoma	Median ST: 8 months: (range: 2–53, no information, if data from start of study or from diagnosis.), 1y OS: 31%, 2y OS: 24%
Gadaleta-Caldarola et al. [80]		EH (Oncotherm EHY2000) + Sorafenib, 60 min. 3 times/week for 6 weeks, followed by 2 weeks without treatment. Period of time: February 2009–September 2010	*n* = 21. Hepatocellular carcinoma	PFS (at four months): 70%. Median TTP (initial treatment until PD): 5.2 months (95%-CI: 4.2–6.2). Median OS (initial treatment to mortality): 10.4 months (95%-CI: 10–11)
Lee et al. [85]		EH (Oncotherm EHY2000) + CTx, 38.5–42.5 °C. for 60 min, every second day. Period of time: April 2006–March 2012	*n* = 23. Small cell lung cancer	ST: range: 2–36 months. Died during treatment: *n* = 7. Survival > 3 years: *n* = 3. No information, if data from start of study or from diagnosis
Yoo et al. [82]		EH (Oncotherm, EHY2000 + , Oncotherm), 2 sessions per week for 3 weeks, CTx before study. Temp: ni. Period of time: October 2008–March 2016	*n* = 19. Recurrent and progressive ovarian cancer	Median overall survival: 8.0 months. Time to progression: ranged from 2.5 to 5.0 months. Time to death ranged from 2.5 to 32.0 months, 18 of 19 patients died
Heo et al. [86]		EH (Celsius42 +) + RTx, 40–43 °C for 60 min. 6 times (range: 3–12 times). Period of time: September 2010–July 2015	*n* = 20. Glioma	Median OS: 8.4 months (95%-CI: 6.9–9.9). 6-month survival: 67%, 12-month survival: 30%. Median PFS: 4.1 months (95%-CI 3.4–4.7). Median 6-month-PFS: 13%. Data from re-irradiation
Yu et al. [83]		EH (Celsius42 +) + RTx, 60 min, skin surface temp.: 36–37.5 °C, twice a week, at intervals of at least 72 h, for 5 total sessions. Period of time: November 2013–August 2014	*n* = 4. Colorectal cancer, hepatic metastasis	Local PFS at 3 months: 30%. Data from start of treatment
Sahinbas et al. [84]		EH (local electrohyperthermia) + CTx, 1 h, during first and second CTx-cycle three times a week. From third CTx-cycle two times a week. Mean: 2.25 cycles of CTx and hyperthermia. Period of time: ni	*n* = 4. Colorectal cancer, hepatic metastases	Mean PFS: 5.2 months. Mean OS: 9.8 months. No information, if data from start of study or from diagnosis
*Outcome: Pain*				
Bull et al. [71]	WBH (Heckel HT-2000) + CTx, 40 °C for 6 h. Cycle repeated up to 7 times. (range: 1–8). Period of time: January 2000–June 2004		*n* = 37. Mixed cancer diagnosis	Pain prior to treatment: *n* = 28 → requiring narcotic drug control. Of them all patients with objective tumour response (*n* = 13) reported decrease of pain and pain medication. 8 of the 13 patients able to stop narcotic pain medication
Koga et al. [75]	ECC-WBH (Parks and Smith) + CTx, 41.5 °C for 3–5 h, 4 times at intervals of 7–10 days (some patients received treatment only once). Period of time: ni		*n* = 17. Gastro-intestinal cancer	Reduction of abdominal cancer pain: *n* = 3
Wiedemann et al. [76]	ECC-WBH (Parks and Smith) + CTx, 41.8 °C for 1 h. Patients received 3 thermo-chemotherapy treatments every 3 weeks. Total of 49 treatments. Period of time: ni		*n* = 19. Sarcoma or malignant teratoma	Improvement after first WBH treatment: n = 4
Ariyafar et al. [87]	EH (Celsius42 +) + RTx 60 min, 2 h after RTx (10 fractions over 2 weeks). Temp: ni. Period of time: December 2016–December 2017		*n* = 23. Bony metastases	1. Median pain score: at T0: ranged from 6 to 8. At T1: significant reductions in the worst pain, least pain, average pain and current pain (*p* < 0.001 for all), maintained during T2–T4 Mean score of worst pain in a 24-h period: at BL: 8.39 (range: 6 to 10), significantly decreased at T1: 4.26 (range: 0 to 9), sustained at T2: 3.74, T3: 3.43 and T4: 3.61 (range: 0 to 9 for all). Similar results observed for least pain, average pain and current pain 2. Pain response (CR: pain score 0 at the worst pain in the preceding 24 h. PR: ≥ 2 drop of the worst pain compared to BL during the preceding 24 h. Stable pain: no change in the score or only pain reduction of 1 score compared to BL at the worst pain during the preceding 24 h over three-months): At T4: CR or PR: *n* = 18 (78%, 95%CI: 61%–95%), refractory to the treatments and stable pain: *n* = 4, variable between stable or partial response: *n* = 2 3. Pain relief medications: Number of patients using pain relief medications: at T0: 74% (*n* = 17), at T1: 52% (*n* = 12), at T4: 48% (*n* = 11) (T0: at BL, T1: treatment completed, T2: 1 month-, T3: 2 months-, T4: 3 months- post-treatment (n = 23))
Yu et al. [83]	EH (Celsius42 +) + RTx, 60 min, Skin surface temp.: 36–37.5 °C, twice a week, at intervals of at least 72 h, for 5 total sessions. Period of time: November 2013–August 2014		1. T1: *n* = 10, T3: *n* = 4 2. T0: *n* = 10, T1: *n* = 5, T2: *n* = 4, T3: *n* = 4. Colorectal cancer, hepatic metastasis	1. Pain response according to IBMCG criteria: at 1 month: PR: n = 4 with SD. At 2 months: PR converted to CR: n = 1, PR: n = 2, SD: n = 1. At 3 months: no change in pain. Pain-PFS: at 3 months: 58.3% 2. Median VAS score: at T0: 4.0 (range: 0–10), at T1: 3.5 (range: 0–7), at T2: 3.0 (range: 0–7), at T3: 0 (range: 0–9) (T0: BL, T1: at 1 month, T2: at 2 months, T3: at 3 months)
*Outcome Quality of life:*				
Steinhart et al. [78]	ECC-WBH (heated air blanket, Cincinnati Sub-Zero hyper-hypothermia machine) alone, 40 °C or 42 °C for 1 h. Period of time: ni		*n* = 6. Kaposi’s sarcoma	40 °C group: no change after WBH, 42 °C group: felt better after WBH
Bruns et al. [61]	WBH (Aquatherm) + CTx, 41.8 °C for 1 h. Responding up to 2 additional cycles. Period of time: April 1999–February 2001		*n* = 22. Pleural mesothelioma	Assessment QoL: + 1,41. Part of the modified Brunner-Score
Bull et al. [71]	WBH (Heckel HT-2000) + CTx, 40 °C for 6 h. Cycle repeated up to 7 times (range: 1–8). Period of time: January 2000–June 2004		*n* = 37. Mixed cancer diagnosis	Clear changes in responding patient
Ariyafar et al. [87]	EH (Celsius42 +) + RTx, 60 min, 2 h after RTx (10 fractions over 2 weeks). Temp.: ni. Period of time: December 2016–December 2017		*n* = 23. Bony metastases	QLQ-C30: during T0 to T4: improvement in all functional scale and symptom scales, except for nausea and vomiting (*p* = 0.455), appetite loss (*p* = 0.764), diarrhoea (*p* = 0.092) and financial difficulties (*p* = 0.055) Compared to T0: physical (*p* = 0.002) and role (*p* = 0.001) functioning, fatigue (*p* < 0.001) and pain (*p* < 0.001) symptoms along with global health status (*p* < 0.001) improved significantly at T4. Emotional (*p* = 0.002) and social (*p* = 0.004) functioning scales improved within T2 and T3 For cognitive functioning (*p* = 0.016), dyspnea (*p* = 0.031), insomnia (*p* = 0.012) and constipation (*p* = 0.031): improvement observed at T2 (T0: at BL, T1: treatment complete, T2: 1 month-, T3: 2 months-, T4: 3 months- post-treatment)
Yu et al. [83]	EH (Celsius42 +) + RTx, 60 min, Skin surface temp.: 36–37.5 °C, twice a week, at intervals of at least 72 h, for 5 total sessions. Period of time: November 2013–August 2014		T0: *n* = 10, T1: *n* = 5, T2: *n* = 4, T3: *n* = 4. Colorectal cancer, hepatic metastasis	HRQoL (EORTC QLQ-C30 and FACT-Hep): no significant differences (T0–T3) (T0: BL, T1: at 1 month, T2: at 2 months, T3: at 3 months)
Yoo et al. [82]	EH (Oncotherm, EHY2000 +), two sessions per week for 3 weeks, CTx before study. Temp: ni. Period of time: October 2008–March 2016		*n* = 7. Ovarian cancer	Fact-O QOL survey: At T1: composite scores and subscale scores decreased in all 16 patients, but no significant change in scores At T2: physical well-being scores significant decreased in *n* = 7 (*p* = 0.044). Social, emotional and functional well-being scores not significantly changed (T0: at BL: *n* = 19, T1: after 3 cycles: *n* = 16, T2: after 6 cycles: *n* = 7)
*Outcome: Haemodynamic parameters:*				
Robins et al. [68]	WBH (Enthermics) + CTx, total of WBH-treatments. at 41.0 °C for 85 min: *n* = 93, at 41.8 °C for 75 min: *n* = 105. Repetition due to escalation temperature scheme. Period of time: ni		*n* = 23. Mixed cancer diagnosis	Episodes of hypotension (within first 6 h post-WBH (systolic blood pressure > 60–80): n = 7. Atypical BP response (> 160/110 mmHg): n = 1
Robins et al. [67]	WBH (Enthermics) alone, 39.5–41.8 °C for 35–140 min, Number of treatments at each level: 3–7, Total of 52 treatments. Period of time: ni		*n* = 8. Mixed cancer diagnosis	Increase in cardiac output and heart rate. Stroke volume remained relatively constant
Robins et al. [70]	WBH (Enthermics) + CTx, 41.8 ± 0.2 °C for 60 min WBH alone in week 1, WBH + CTx in week 2, CTx alone in week 5. Responding patients: WBH + CTx to maximum of further 5 cycles. Period of time: ni		*n* = 30. Mixed cancer diagnosis	Asymptomatic hypotension post-WBH (systolic blood pressure: 80–90 mmHg): n = 2
Barlogie et al. [73]	WBH (water blankets, Cincinnati Sub-Zero) alone or WBH + CTx, 42 °C for 4 h. Frequency: Ø 3x. Period of time: June 1977–April 1978		*n* = 12. Mixed cancer diagnosis	HR: increased significantly from average: 91/min to 131/min during heating (*p* = 0.001), rapid return to pre-treatment conditions within 12 h. SBP: no significantly change during WBH. DBP: dropped significantly, average of 73 mmHg prior to a mean of 60 mmHg during WBH (*p* < 0.01), rapid return to pre-treatment values within 12 h
Bull et al. [88]	WBH (highflow, heated-water perfusion suit enclosed in insulated cover, Webb Associates) alone, 39.5–41.8 °C for 1–4 h. N = 4 repeated exposures at 2 to 3-week intervals at 41.8 °C for 4 h for 6–26 procedures. Period of time: ni		*n* = 14. Mixed cancer diagnosis	1. HR (beats/min): T0: 88.0 ± 4.0, T1: 160.0 ± 9.0. MAP (mm Hg): T0: 89.0 ± 7.0, T1: 69.0 ± 4.0 (T0: BL, T1: at 41.8 °C, T2: 24 h after WBH procedure). Pulmonary capillary wedge pressure (mmHg) T0: 9.0 ± 1.0, T1: 5.0 ± 1.0. CaI (litre/min-m^2^): T0: 3.3 ± 0.2, T1: 7.2 ± 0.3. SBP: 70–90 mmHg for 30 min 3 h post-treatment: n = 5 2. Exposure at 41.8°: 2 h versus 1 h: cardiovascular variables: no difference
Gerad et al. [74]	WBH (nylon and vinyl mesh water perfused suit (Whittaker General Medical), heating blankets) + CTx, 41.8–43.0 °C for 2 h, Total of 35 treatments, Period of time: ni		*n* = 11. Soft tissue Sarcoma	Significant mean changes: HR and respiratory rate: rise, DBP: decline. Once temperature max. (Tmax) reached, only minor changes → cooling to 37 °C significant reduction of levels observed at Tmax. All parameters returned to near BL levels by 24 h
Locker et al. [89]	ECC-WBH (Rota-Flow) alone, 41.8 ± 0.2C° for 120 min. Number of cycles for each patient: range: 1–4. Period of time: ni		*n* = 6. Soft tissue Sarcoma	HR, CaI, stroke volume index: significantly increased (*p* < 0.05). BP, pulmonary vascular RI: significantly decreased (*p* < 0.05). Fluid balance: 5822 ± 1766 mL per heating period. Low doses of norepinephrine required to maintain MAP > 60 mmHg, rapidly tapered reaching normothermia
Wiedemann et al. [76]	ECC-WBH (Parks and Smith) + CTx, 41.8 °C for 1 h. Patients received 3 thermo-chemotherapy treatments every 3 weeks. Total of 49 treatments. Period of time: ni		*n* = 19. Sarcoma or malignant teratoma	HR and cardiac output: increased with rising core temperature, HR rose more than the stroke volume. Stable MAP achieved by fluid substitution and catecholamines
Wiedemann et al. [77]	ECC-WBH (Parks and Smith) + CTx, 41.8 °C for 1 h. Period of time: ni		*n* = 12. Sarcoma	HR and cardiac output: increased with rising core temperature, HR rose more than the stroke volume. Stable MAP achieved by fluid substitution and catecholamines. MAP pre-treatment: 116.4 ± 10.7 mmHg, at 41.8 °C: 82.4 ± 8.6 mmHg
Steinhart et al. [78]	ECC-WBH (heated air blanket, Cincinnati Sub-Zero hyper-hypothermia machine) alone, 40 °C or 42 °C for 1 h. Period of time: ni		*n* = 6. Kaposi’s sarcoma	MAP: decreased modestly at 40 °C group and decreased markedly at 42 °C group. CaI: increased modestly at 40 °C, rose 100% or more in 42 °C group. End-diastolic index increased during warming phase in both groups, during hyperthermia increased in response to fluid challenge more predictable than capillary wedge pressure
Lee et al. [90]	EH (Oncotherm EHY2000) alone, 38.5 ± 0.8 °C for 60 min. Period of time: ni		*n* = 20. Cervical carcinoma	1. S/D ratio (mean ± SD) with BC-p-values, comparison with BL: T0: 1.65 ± 0.20, T1: 1.40 ± 0.13, T2: 1.22 ± 0.09, T3: 1.40 ± 0.16. T1: *p* < 0.001, at T2: *p* < 0.001, at T3: *p* < 0.001 2. RI (mean ± SD) with BC-p-values, comparison with BL: T0: 0.40 ± 0.12, T1: 0.29 ± 0.11, T2: 0.19 ± 0.06, T3: 0.30 ± 0.10. T1: *p* < 0.01, T2: *p* < 0.001, T3: *p* < 0.05 (T0: 30 min before EH, T1: 30 min during EH, T2: 60 min during EH, T3: 30 min after EH)
*Outcome: Haematological and serum chemistry profiles*				
Katschinski et al. [63]	WBH (Aquatherm) + CTx, 41.8 °C for 60 min. Total of 53 cycles. Period of time: ni		*n* = 17. B-cell neoplasms	Comparison clinical parameters early versus late CTx-schedule: Late CTx-schedule significant (p < 0.05) clinical advantage. Delay in CTx secondary to thrombocytopenia and neutropenia: late schedule: 22 days versus early schedule: 95 days, (Chi^2^: 0.15 versus 1.3). Incidence of plated transfusions: late schedule: 5 transfusions versus early schedule: 40 transfusions (Chi^2^: 0.3 vs. 1.5). Unanticipated hospitalization secondary to thrombocytopenia: late schedule: 4 hospital days versus early schedule: 56 hospital days
Robins et al. [67]	WBH (Enthermics) alone, 39.5–41.8 °C for 35–140 min. Number of treatments at each level: 3–7. Total of 52 treatments. Period of time: ni		*n* = 12. Mixed cancer diagnosis	Mean values post-therapy (${\mathrm{Mg}}^{2+},$ ${\mathrm{Ca}}^{2+}$ and ${\mathrm{PO}}_{4}^{2-}):$ normal range, within 1 SMD of pretreatment mean value. Liver status (LDH, AP, GOT): no changes: *n* = 7, transient elevation: *n* = 3, tumour lysis syndrome with increased LDH: *n* = 1, hepatic change (LDH levels with increase of 60%) and tumour lysis syndrome: *n* = 1. CPK: significant elevation after WBH: *n* = 1 with no clinical symptoms. WBC count: no trends as WBH dose escalated. WBC did not change post-WBH. Platelet count: no trends as WBH dose escalated. Fibrinogen levels, prothrombin time, partial thromboplastin time, fibrin split products: clinically normal range during and after treatment
Robins et al. [68]	WBH (Enthermics) + CTx, total of WBH-treatments. at 41.0 °C for 85 min: n = 93, at 41.8 °C for 75 min: n = 105. Repetition due to escalation temperature scheme. Period of time: ni		*n* = 23. Mixed cancer diagnosis	Course of haematological and chemistry profiles (blood count, WBC, prothrombin- and partial thromboplastin time, liver function tests, electrolytes, ${\mathrm{Ca}}^{2+}$, ${\mathrm{Mg}}^{2+}$ and CPK) pre-treatment, 24 h and 48 h post-WBH: only slight changes
Robins et al. [69]	WBH (Enthermics) + RTx, 41.8 °C for 75 min, total of 97 WBH-treatments. Period of time: November 1983–April 1987		*n* = 8. B-cell neoplasms	24 h and 48 h post-WBH/RTx: creatinine, liver function, bilirubin, electrolytes, haematocrit, prothrombin- and partial thromboplastin time: no significant changes. No substantial immediate effects on WBC counts, platelet counts, or differential counts after the administration of WBH + RTx (T0: BL, T1: at peak temperature, T2: 24 h after treatment)
Robins et al. [70]	WBH (Enthermics) + CTx, 41.8 ± 0.2 °C for 60 min WBH alone in week 1, WBH + CTx in week 2, CTx alone in week 5. Responding patients: WBH + CTx to maximum of further 5 cycles. Period of time: ni		*n* = 30. Mixed cancer diagnosis	Difference WBC- and platelet nadirs: WBH + CTx versus CTx alone: not significant (WBC: *p* < 0.74, platelet: *p* < 0.75). Percent change in platelet count correlated well with AUC for ultra-filterable platinum (*r* = 0.86, *p* < 0.001). Total clearance of platinum correlated well with the creatinine clearance (*r* = 0.790, *p* < 0.001)
Kraybill et al. [72]	WBH (Heckel, HT-2000) alone, group A: 39–39.5 °C for 3 h, group B: 39–39.5 °C for 6 h, group C: 39.5–40 °C for 6 h. Period of time: ni		*n* = 9. Mixed cancer diagnosis	WBH no impact on red cell mass or platelets. Patients heated for 6 h: increases in total numbers of WBC directly following WBH treatment. Increases in granulocytes and monocytes. Majority of patients: transient decreases in T-lymphocytes and L-selectin positive lymphocytes
Barlogie et al. [73]	WBH (water blankets, Cincinnati Sub-Zero) alone or WBH + CTx, 42 °C for 4 h. Frequency: Ø 3x. Period of time: June 1977–April 1978		*n* = 12. Mixed cancer diagnosis	WBH-associated significant changes: (mean ± SD): platelets (× 10^3^/litre): T0: 243 ± 65, T1: 224 ± 91, T2: 147 ± 75 → most patients recovered from thrombocytopenia within 1 week. Prolongation in prothrombin time (average of 4 s) and partial thromboplastin (average of 6 s) during initial 24 h after WBH. CPK (units/litre): T0: 50 ± 60, T1: 80 ± 92, T2: 399 ± 621 (excluding n = 1 with severe rhabdomyolysis, CPK: 40,000 units/litre). Subsequent courses associated with progressively smaller CPK elevations (p = 0.001). Glucose (mg/dl): T0: 109 ± 27, T1: 223 ± 98, T2: 160 ± 98 → normoglycaemia within 48 h after WBH. Significant alterations in electrolytes: hypocalcaemia: minimum average of 8.5 mEq/litre at T1, hypomagnesemia: 1.3 mEq/litre at T2, hypophosphatemia of 1.8 mEq/litre at T1 and hypokalemia with mean potassium concentration of 3.2 mEq/litre at T2. No significant elevations of GOT and LDH (T0: pre-treatment, T1: during WBH, T2: after 24 h)
Bull et al. [88]	WBH (highflow, heated-water perfusion suit enclosed in insulated cover, Webb Associates) alone, 39.5–41.8 °C for 1–4 h. N = 4 repeated exposures at 2 to 3-week intervals at 41.8 °C for 4 h for 6–26 procedures. Period of time: ni		*n* = 14. Mixed cancer diagnosis	Serum-CPK: elevated at T2 in comparison with T0. Creatinine and creatinine clearance, sodium, potassium, chloride, bicarbonate, BUN, serum protein, albumin, bilirubin, LDH, AP: no significantly change during T0-T2. Serum phosphate: T0 median: 3.5 mg/dl (range: 2.3–4.0 mg/dl) at the end of treatment: 1.0 mg/dl (range: 0.6–1.5 mg/dl). Values returned to normal levels by 36 h. Magnesium: T0 median: 1.7 meq/litre at end of treatment: 1.3 meq/litre, returned to normal range by 24 h. Phosphate and magnesium changes: due to respiratory alkalosis. Transient elevation of GOT (T0: 27 U/litre. T2: 68 U/litre) and GPT (T0: 26 U/litre. T2: 97 U/litre): n = 5, in normal range within 6 days. Leucocyte count: median at T0: 7.8 × 10^3^, at T1: elevated to median: 11.5 × 10^3^ cells (range: 7.5–32.5). Granulocyte count median at T0: 6.5 × 10^3^, at T1: elevated to: median 10.9 × 10^3^ cells (range: 6.0–28.7). Lymphocyte count: insignificant fall from: median 1.2 × 10^3^ cells to 0.9 × 10^3^ cells (range: 0.3–1.4) →counts returned toward normal values at 24 h. Coagulation parameters: no significant alteration during T0–T2. No significant changes of haemoglobin level or platelet count (T0: BL, T1: at 41.8 °C, T2: 24 h after WBH procedure)
Gerad et al. [74]	WBH (nylon and vinyl mesh water perfused suit (Whittaker General Medical), heating blankets) + CTx, 41.8–43.0 °C for 2 h. Total of 35 treatments. Period of time: ni		*n* = 11. Soft tissue Sarcoma	At 41.8 °C: significant (*p* < 0.05) shift in: sodium, chloride, bicarbonate, BUN, glucose, creatinine, total bilirubin, calcium, phosphorus and CPK compared to BL. Liver enzymes significant delayed change 24 h post-treatment (GOT, GPT, LDH: increase, AP: decrease) Follow-up: return to BL or normal range for all values. No significant change in prothrombin- and partial thromboplastin time, thrombin time, or fibrinogen levels. No evidence of disseminated intravascular coagulation Mean WBC nadirs: 1620 µl ± 305 (18 euthermic treatments) versus 1590 µl ± 235 (32 WBH treatments): no significantly difference. Platelet count nadirs (mean ± SD): at BL: 285.6 10^3^/µl ± 21.4, 24 h after WBH: 177.9 10^3^/µl ± 12.7: significant decrease (*p* = 0.0001). Fall in haemoglobin between 1 or 2 g/dl in all patients over first 48 h post-WBH due to dilution, blood sampling, and possibly heat. Leucocyte differentials: immediate leucocytosis resolved over 2–3 days
Locker et al. [89]	ECC-WBH (Rota-Flow) alone, 41.8 ± 0.2 °C for 120 min. Number of cycles for each patient: range: 1–4. Period of time: ni		*n* = 6, 12 treatments. Soft tissue sarcoma	Hypocalcemia: (grade 1): 8%, (grade 2): 42%. Hypophosphatemia: (grade 2): 25%, (grade 3): 50%. Hypomagnesemia: (grade 1): 33%. Hypopotassemia: (grade 1): 42%. Hypermagnesemia: (grade 1): 33%. Hyperchloremia: (grade 1): 8%. Hypernatremia: (grade 1): 8%. Hyperbilirubinemia: (grade 1): 33%, (grade 2): 17%, (grade 3): 25%. Hypoalbuminemia: (grade 1): 50%. Elevated lipase: (grade 1): 8%, (grade 2): 8%, (grade 3): 17%. AST elevation: (grade 1): 33%, (grade 2): 25%, (grade 3): 17%, (grade 4): 17%. ALT elevation: (grade 1): 33%, (grade 2): 8%, (grade 3): 25% (grade 4): 17%. GGT elevation: (grade 1): 17%, (grade 2): 8%. Elevated phosphatase: (grade 1): 17%. Amylase elevation: (grade 3): 33%. Hypoglycaemia: (grade 1): 8%. Hyperglycaemia: (grade 1): 8%. Creatinine elevation: (grade 1): 8%. CPK elevation: (grade 1): 33%, (grade 2): 25%, (grade 4): 8%. Troponin T elevation: (grade 2): 8%. Anaemia: (grade 1): 42%. Thrombocytopenia: (grade 1): 25% (grade 2): 17%, (grade 3): 33%, (grade 4): 25%. Thrombocytes significantly decreased with a nadir at 24 h after ECC-WBH (*p* < 0.05), but spontaneously resolved during the following days. Leucopenia: (grade 1): 25%, (grade 2): 8%. Neutropenia: (grade 1): 8%. Haemolysis: (grade 1): 33%. PTT prolongation: (grade 1): 25%
Worel et al. [91]	ECC-WBH (Rota-Flow) alone, 41.8 ± 0.2 °C for 120 min. Number of cycles for each patient: range: 1–4. Period of time: ni		Included: *n* = 6, analysed: 12 treatments. Soft tissue sarcoma	T1 versus T0: coagulation alterations most likely due to anticoagulation. (70 U/kg of UFH (unfractionated heparin)) →significant increase of aPTT (> 60 s). PT, fibrinogen, D-dimers, platelet counts and liver enzymes remained stable T2 versus T1: Effect of initially applied UFH declined (aPTT: 46.3 ± 2.9 s). Mild but significant signs of coagulation activation: increase of D-dimers. Thrombocytopenia (platelet counts: slightly but significant decrease, within normal range (173 ± 24 g/l)). Liver enzymes (AST, ALT, bilirubin): significant increase, but not clinically relevant T3 versus T2: D-dimer: significantly increased. Platelet counts: significantly decreased (58 ± 34 g/l, in 50% of treatments: platelets < 50 g/l). AST, ALT, bilirubin: significant increase (*p* < 0.05), AP remained within normal range T4 versus T2: PT, fibrinogen, and AT III (anti-thrombin III). Significantly increased (*p* < 0.05, exceeded BL values). D-Dimer decreased, but remained above normal range. AST and bilirubin: decreased to nearly normal values. ALT and AP: further increase. ALT remained above normal range. Platelet counts exceeded BL counts (T4: 287 ± 61 vs. BL: 195 ± 21 g/l. *p* < 0.05) (T0: BL, T1: after 30 min on normothermic ECC, T2: end of heating period, T3: 24 h after ECC-WBH, T4: 8 days after ECC-WBH) Changes in platelet counts and liver enzymes tended to correlate, but not significant. (AST vs. platelets *R*^2^ = 0.49)
Koga et al. [75]	ECC-WBH (Parks and Smith) + CTx, 41.5 °C for 3–5 h, 4 times at intervals of 7–10 days (some patients received treatment only once). Period of time: ni		*n* = 17. Gastro-intestinal cancer	Thrombocytopenia (7 × 1 ${0}^{4}$/mm^3^): *n* = 13 (76.5%), between 1–3 days after ECC-WBH. Leucocytopenia (< 3 × 10^3^/mm): *n* = 8 (47.1%), time to leucocyte count nadirs not uniform. Serum GOT, GPT, LDH and AP levels little elevated. Pretreatment serum total bilirubin level (0.9 ± 0.3 mg/dl) significantly elevated only on the third day after ECC-WBH (1.4 ± 0.5 mg/dl. *p* < 0.05), but declined to pretreatment level after the fifth day. Creatinine and urea nitrogen levels little affected and marked urine abnormalities not shown. Pre-treatment serum CPK level (32 ± 41 mU/ml) significantly elevated on first day after ECC-WBH (164 ± 143 mU/ml. *p* < 0.05), but decreased gradually
Wiedemann et al. [76]	ECC-WBH (Parks and Smith) + CTx, 41.8 °C for 1 h. Patients received 3 thermo-chemotherapy treatments every 3 weeks. Total of 49 treatments. Period of time: ni		*n* = 19. Sarcoma or malignant teratoma	CTx without ECC-WBH: WBC nadir: 2.2 ± 0.37 k/ml (range: 1.6–3.9), platelet nadir: 67 ± 19 k/ml (range: 29–122). CTx with ECC-WBH: WBC nadir: 2.6 ± 0.41 k/ml (range: 1.7–4.9), platelet nadir 58 ± 17 k/ml (range: 27–130) →differences not statistically significant → bone marrow toxicity of given CTx not increased by WBH. Liver parameters (LDH, AP, AST): no change after WBH: *n* = 5. transient LDH elevation without rise of AP and AST: *n* = 11. LDH twice pretreatment value, returned to pre-WBH values 3 days after last thermo-chemotherapy: *n* = 1
Steinhart et al. [78]	ECC-WBH (heated air blanket, Cincinnati Sub-Zero hyper-hypothermia machine) alone, 40 °C or 42 °C for 1 h. period of time: ni		*n* = 6. Kaposi’s sarcoma	40 °C group: no significant changes. 42 °C group: modest increase in CPK, GOT, GPT and bilirubin. Serum phosphate levels fell slightly at end of WBH. Platelet count fell and bicarbonate, prothrombin time and free haemoglobin increased in 42 °C group. None of changes associated with clinical symptoms and all normalized by end of follow-up period
Yu et al. [83]	EH (Celsius42 +) + RTx, 60 min, skin surface temp.: 36–37.5 °C, twice a week, at intervals of at least 72 h, for 5 total sessions. Period of time: November 2013–August 2014		at 1 month: *n* = 10, at 3 months: *n* = 4. Colorectal cancer, hepatic metastasis	Haemoglobin, platelet, AST, ALT, albumin, total bilirubin, creatinine: of them significant changes at 1-, 2-, 3-month-follow up: platelets: (cells/μL) BL: 232 (range: 132–560), at 1 month: 121 (range: 40–227) (p = 0.008), at 3 months: 241 (range: 115–329). Creatinine (mg/dl): BL: 0.72 (range: 0.59–1.09), at 1 month: 0.65 (range: 0.46–0.97) (*p* = 0.002), at 3 months: 0.71 (range: 0.57–0.85)
*Outcome: Pharmacokinetics of CTx*				
Robins et al. [68]	WBH (Enthermics) + CTx, total of WBH-treatments. at 41.0 °C for 85 min: *n* = 93, at 41.8 °C for 75 min: *n* = 105. Repetition due to escalation temperature scheme. Period of time: ni		*n* = 23. Mixed cancer diagnosis	Difference lonidamine-serum levels: before versus after WBH: No significant difference → WBH no significant effect on pharmacokinetics of lonidamine
Robins et al. [70]	WBH (Enthermics) + CTx, 41.8 ± 0.2 °C for 60 min		*n* = 30. Mixed cancer diagnosis	Analysis of platinum in plasma ultrafiltrate and urine: WBH no significant effect on pharmacokinetics and renal excretion of platinum
Wiedemann et al. [76]	ECC-WBH (Parks and Smith) + CTx, 41.8 °C for 1 h. Patients received 3 thermo-chemotherapy treatments every 3 weeks. Total of 49 treatments. Period of time: ni		*n* = 19. Sarcoma or malignant teratoma	Area under the curve of CTx: 37 °C versus 41.8 °C: significantly different (*p* < 0.001) →one-third reduction of 4-Hydroxyifosfamide (activated intermediate metabolite of Ifosfamide and Carboplatin), due to loss by haemodialysis. But increase of Chloroacetaldehyde (Ifosfamide metabolite)
*Outcome: Course of tumour marker*				
Atmaca et al. [59]	WBH (Aquatherm) + CTx, 41.8 °C for 1 h. Cycles repeated every 28 days up to a total of 6 cycles. Median: 2.7 (range: 1–6). Period of time: May 1997–December 2002		*n* = 30. Ovarian carcinoma	CA 125: response (serum CA 125 decrease > = 50% of BL): *n* = 18, biochemical progress: *n* = 7, no change: *n* = 5
Richel et al. [64]	WBH (Aquatherm) + CTx, 41.8 °C for 1 h. Total of 82 courses, median of 3 per patient (range: 1–6). Period of time: ni		*n* = 9 (stable patients analysed). Cervical cancer	CA 125, SCC-Ag: Substantial marker decrease (> 50%): *n* = 5, increase (> 50%): *n* = 1
*Outcome: modified Brunner-Score: (integrates: PFS, change of physical performance, quality of life self-assessment, toxicity)*				
Bruns et al. [61]	WBH (Aquatherm) + CTx, 41.8 °C for 1 h. Responding up to 2 additional cycles. Period of time: April 1999–February 2001		*n* = 22. Pleural mesothelioma	MBS for overall study group: 4.21 points (range: − 4.43–16.45), 16 of 22 patient achieved positive score. Subgroups of MBS: improvement of performance index: + 0.29, QoL: + 1.41
*Outcome: Body weight*				
Steinhart et al. [78]	ECC-WBH (heated air blanket, Cincinnati Sub-Zero hyper-hypothermia machine) alone, 40 °C or 42 °C for 1 h. Period of time: ni		*n* = 6. Kaposi’s sarcoma	40 °C group: no change, 42 °C group: gained weight
Bull et al. [71]	WBH (Heckel HT-2000) + CTx, 40 °C for 6 h. Cycle repeated up to 7 times (range: 1–8). Period of time: January 2000-–June 2004		*n* = 37. Mixed cancer diagnosis	n = 29 reported weight loss of 5–35 pounds prior to treatment. 14 of the 16 responding patients with weight loss regained weight (range: 45–100%, median: 76%)
*Outcome: Heat dose tolerance*				
Bull et al. [88]	WBH (highflow, heated-water perfusion suit enclosed in insulated cover, Webb Associates) alone, 39.5–41.8 °C for 1–4 h. 4 repeated exposures at 2 to 3-week intervals at 41.8 °C for 4 h for 6–26 procedures. Period of time: ni		*n* = 14. Mixed cancer diagnosis	1. Heat escalation over weeks versus initial heat exposure over 1 h at 41.8 °C: no difference in tolerance 2. Exposure at 41.8 °C: 2 h versus 1 h: increased fatigue treated 2 h
Robins et al. [67]	WBH (Enthermics) alone, 39.5–41.8 °C for 35–140 min, number of treatments at each level: 3–7. Total of 52 treatments. Period of time: ni		*n* = 12. Mixed cancer diagnosis	At core temperature (41.8 °C): average maximum skin temperature: 42.66 ± 0.58 °C Temperatures in bladder: close to rectal temperatures but differed from concurrent esophageal temperatures. Axillary profiles of 3 patients treated at 41.8 °C: temperature of 41.8 °C in pulmonary artery achieved → patient covered with blankets and a vapour barrier and removed from apparatus → rectal temperature of 41.8 °C achieved about 10 min later, after plateau phase → coverings removed: pulmonary artery temperature decreased immediately, drop in blood temperature precedes fall in rectal temperature
Lee et al. [90]	EH (Oncotherm EHY2000) alone, 38.5 ± 0.8 °C for 60 min. Period of time: ni		*n* = 20. Cervical carcinoma	Peri-tumour temperature (mean ± SD) with BC-p-values, comparison with BL: T0: 36.7 ± 0.2 °C, T1: 37.5 ± 0.5 °C, T2: 38.5 ± 0.8 °C, T3: 37.1 ± 0.3 °C. T1: *p* < 0.001, T2: *p* < 0.001, T3: *p* < 0.05 (T0: 30 min before EH, T1: 30 min during EH, T2: 60 min during EH, T3: 30 min after EH)
*Outcome: Fatigue*				
Bull et al. [71]	WBH (Heckel HT-2000) + CTx, 40 °C for 6 h. Cycle repeated up to 7 times (range: 1–8). Period of time: January 2000–June 2004		*n* = 37. Mixed cancer diagnosis	N = 34 reported grade 1–2 fatigue, with n = 1 reporting grade 3 fatigue prior to treatment. All 16 patients with objective tumour response reported increased energy, improved sense of well-being and 15 resumed normal activities, including n = 7 resuming former employment
*Outcome: Respiratory parameters*				
Robins et al. [67]	WBH (Enthermics) alone, 39.5–41.8 °C for 35–140 min. Number of treatments at each level: 3–7. Total of 52 treatments. Period of time: ni		*n* = 12. Mixed cancer diagnosis	pH: BL: mean pH value: 7.42 ± 0.02, treated at 39.5–40.5 °C: mean 7.38 ± 0.05, treated at 41.5–41.8 °C mean: 7.38 ± 0.06. Arterial CO_2-_tension: normal during WBH. Serum lactate at plateau: 2.53 ± 0.08 mmol/l. At core temperature: rise in oxygen consumption. Arterial and venous oxygen saturation: normal, even in patients not receiving nasal oxygen
Barlogie et al. [73]	WBH (water blankets, Cincinnati Sub-Zero) alone or WBH + CTx, 42 °C for 4 h. Frequency: Ø 3x. Period of time: June 1977–April 1978		*n* = 12. Mixed cancer diagnosis	No significant changes in pH and base deficit (pre-treatment to 24 h after treatment)
Bull et al. [88]	WBH (highflow, heated-water perfusion suit enclosed in insulated cover, Webb Associates) alone, 39.5–41.8 °C for 1–4 h. N = 4 repeated exposures at 2 to 3-week intervals at 41.8 °C for 4 h for 6–26 procedures. Period of time: ni		*n* = 14. Mixed cancer diagnosis	Thermally induced hyperventilation → respiratory alkalosis with median arterial pH: 7.5 ± 0.05 and arterial PaCO_2_: range 18–20 mmHg. Respiratory rate: increased from median of 11 ± 3 to 38 ± 5 at 41.8 °C. Arterial oxygen saturation remained unchanged throughout treatment
Locker et al. [89]	ECC-WBH (Rota-Flow) alone, 41.8 ± 0.2 °C for 120 min. Number of cycles for each patient: range: 1–4. Period of time: ni		*n* = 6. Soft tissue sarcoma	Oxygen delivery and consumption: significantly increased during ECC-WBH (*p* < 0.03). Respiratory rate: initially dropped, but then significantly increased during heating (*p* < 0.05), remained elevated during heating period. Arterial pH: significant changes over time (increase), but within normal range. PaCO_2_: moderate increase on normothermic ECC, significantly decrease during plateau phase (*p* < 0.05). Standard bicarbonate and base excess: continuously decreased (*p* < 0.05) until end of WBH. Lactate level: significant elevation up to .6 mmol·${\mathrm{L}}^{-1}$ (*p* < 0.05)

*y OS* 1 year-overall-survival; *2y OS* 2 year-overall-survival. *ALT* alanine transaminase. *Anal.* Analysed; *AP* alkaline phosphatase; *APr* arterial pressure; *aPTT* activated partial thromboplastin time; *ARDS* acute respiratory distress syndrome; *AST* aspartate aminotransferase; *AUC* area under the curve; *AV* atrioventricular; *BC* Bonferroni-corrected; *BL* baseline; *BP* blood pressure; *BUN* blood urea nitrogen; *CaI* cardiac Index; *CI* confidence-interval; *CPK* creatine phosphokinase; *CR*: complete response CTCAE common terminology criteria for adverse events version; *CTx* chemotherapy; *DBP* diastolic blood pressure; *DCR* disease control rate; *DIC* disseminated intravascular coagulation; *ECC-WBH* extracorporeal-circulation-WBH; *ECOG*Eastern Cooperative Oncology Group. *EEG* electroencephalogram. *EORTC* European Organization for Research and Treatment of cancer; *EH* electro hyperthermia; *FACT-Hep* Functional Assessment of Cancer Therapy-hepatobiliary; *GFR* glomerular filtration rate; *GGT* gamma-glutamyl transferase; *GOT* glutamic oxaloacetic transaminase; *GPT* glutamic pyruvic transaminase; *h* hours; *HR* heart rate; *HRQoL* health-related quality of life; *IBMCG* International Bone Metastases Consensus Group (IBMCG); *Iv* Intra-venous; *Incl* included; *KS* Kaposi’s sarcoma; *LDH* lactic dehydrogenase; *MAP* mean-arterial blood pressure; *MBS* modified Brunner Score; *MCP* Metoclopramid; *min* minutes; *MR* minor response; *MTD* maximum tolerated dose; *n* number of patients; *NC* no change; *NCICTC* National Cancer Institute Common Toxicity Criteria; *ni* no information; *ORR* objective response rate; *OS* overall survival; *PD* progressive disease; *PFS* progression free survival; *PR* partial response; *PTT* partial thromboplastin time; *QLQ-C30* European Organization for the Research and Treatment of Cancer Quality of Life Questionnaire; *QoL* quality of Life; *RI* resistance index; *RR* response rate; *RTx* radiotherapy; *S/D ratio* peak systolic velocity/end-diastolic velocity ratio; *SBP* systolic blood pressure; *Scc-Ag* squamous cell carcinoma antigen; *SCR* serologic complete response; *SD* stable disease; *SIRS* systemic inflammatory response syndrome; *SMD* standardized mean deviation; *ST* survival time; *Tmax* maximum temperature; *TTF* time to treatment failure; *TTP* time to progression; *UFH* unfractionated heparin; *VAS* visual analogue scale; *vs* versus; *WBC* white blood cell count; *WBH* whole-body hyperthermia

**Table 5:** Efficacy of hyperthermia in case reports

Reference	Intervention, Duration of target temp. per session, Period of time of the study	Number of participants, Cancer Type	Outcome
*Outcome: Tumour response*			
Bakhshandeh et al. [92]	WBH (Aquatherm) + CTx, 41.8 °C for 1 h. Period of time: ni	*n* = 1. Pleural mesothelioma	PR: *n* = 1
Sagowski et al. [93]	WBH (Enthermics, RHS-7500) + CTx, 41.8 °C for 60 min. Period of time: ni	*n* = 1. Squamous cell carcinoma of oral cavity	PR: *n* = 1
Jeung et al. [94]	EH (Oncotherm EHY2000 +) + CTx or RTx, or EH as monotherapy than combination not feasible, 60 min. 2∼3 times/week, 12 times in one cycle Average number of treatments: 33 sessions in 4 cycles. Period of time: Start: December 2011 and summarized results until September 2012	Included: *n* = 216, chosen 16 cases characteristically shown. Mixed cancer diagnosis	Different results for each case. In most of the reported cases: good response results
Yeo et al. [95]	EH (Oncotherm EHY2000) + RTx, 60 min, 2 sessions per week, for a total of 12 sessions. Period of time: ni	*n* = 1. Non-small-cell Lung cancer	CR: *n* = 1
*Outcome: Temperature*			
Sagowski et al. [93]	WBH (Enthermics, RHS-7500) + CTx, 41.8 °C for 60 min. Period of time: ni	*n* = 1. Squamous cell carcinoma of oral cavity	With latency of 10 min, increase of intra-tumoural temperature in oral cavity, maximum: 41.8 °C, comparable to esophageal and rectal temperatures
*Outcome: Tumour oxygenation*			
Sagowski et al. [93]	WBH (Enthermics, RHS-7500) + CTx, 41.8 °C for 60 min. Period of time: ni	*n* = 1. Squamous cell carcinoma of oral cavity	Average increase > = 100% in each cycle, also significantly improved in tumour in head and neck area despite the fact that this area outside hyperthermia chamber
Pereira Arias et al. [96]	WBH (Aquatherm) + CTx, 41.8 °C for 1 h. Period of time: first treatment: 22th April 1997, died 2th May 1997	*n* = 1. Leiomyosarcoma of uterus	After treatment: recovery within 2 days: full consciousness, could be extubated, inotropic support stopped and creatinine returned to pre-treatment levels. All cultures remained sterile. After almost complete recovery, 5 days later, second episode of fever during neutropenia and, despite antibiotic treatment, died of sepsis
Feyerabend et al. [97]	ECC-WBH (Parks and Smith) + CTx, 41.8 °C for 60 min. Period of time: ni	*n* = 1. Embryonal testicular cancer	After > 5 years of follow up, patient alive and disease-free, patient refused further therapy as well as follow-up examinations

*CR* complete response; *CTx* chemotherapy; *ECC-WBH* extracorporeal WBH; *EH* electro hyperthermia; *n* number of patients; *ni* no information; *OS* overall survival; *PR* partial response; *QoL* Quality of Life; *RTx* radiotherapy; *ST* survival time; *Temp.* temperature; *WBH* whole-body-hyperthermia; *WHO* World-Health-Organization

**Table 6:** Adverse events in studies of the second level of evidence (single-arm studies, case series and case reports)

Side effect	Reference	Specific/Grade (n)
*Related to WBH*		
Skin lesions	Westermann et al. [66]	(grade 1): 0.3%, (grade 2): 0.3%. Redness or blisters (grade 1) on pressure spots: 5%
	Atmaca et al. [59]	(grade 1 and 2): 4%
	Richel et al. [64]	Painless self-limiting blisters: *n* = 3 (12%)
	Bakhshandeh-Bath et al. [60]	(grade 1): 3%, (grade 3): 3%
	Bakhshandeh-Bath et al. [62]	(grade 1): 8%
	Kraybill et al. [72]	Group 39–39.5 °C for 6 h: small blisters middle and index fingers of right hand and 4 knuckles left hand: *n* = 1 (33%). Group 39.5–40 °C for 6 h: blister at site of oxygen monitor: *n* = 1 (33%)
	Bull et al. [71]	Burn: (grade 2): 3%
	Bull et al. [88]	Skin: initially, several pressure ulcers on heels and occasional small burns. Problems alleviated by positioning and insulating pressure points
	Barlogie et al. [73]	Burns: (grade 1): *n* = 8 (67%), (grade 2): *n* = 5 (42%), (grade 3): *n* = 11 (8%)
	Gerad et al. [74]	Burns (grade 1 or 2): 37% of treatments, at contact points with heating pads
Herpes infection	Westermann et al. [65]	Mucosal, responsive to valacyclovir: *n* = 4 (29%)
	Robins et al. [68]	Involving lips: *n* = 7 (30%)
	Robins et al. [69]	Oral (grade 1): *n* = 2 (25%)
	Robins et al. [70]	*n* = 5 (17%) (all patients with history of herpes simplex infection experienced a recurrence with every WBH treatment)
	Barlogie et al. [73]	*n* = 6 (50%)
	Gerad et al. [74]	Perioral: *n* = 8 (73%), all after first WBH-treatment
Headache	Westermann et al. [65]	(grade 1): *n* = 1 (7%)
	Robins et al. [68]	During first 6 h post-WBH: *n* = 5 (22%)
	Robins et al. [69]	(grade 1 and 2): *n* = 4 (50%)
	Robins et al. [70]	(grade 1): *n* = 2 (7%)
	Kraybill et al. [72]	Group: 39.5–40 °C for 6 h (*n* = 3): frontal headaches: *n* = 2 (67%)
Fevers	Westermann et al. [65]	Post-WBH low grade fevers (lasting about 24 h): *n* = 2 (14%)
	Robins et al. [68]	*n* = 3 (13%)
	Bull et al. [88]	Post-hyperthermia: *n* = 4 (19%) (38.5 °C–40.2 °C, 18–24 h after WBH)
	Barlogie et al. [73]	> 38 °C (24–36 h after treatment): *n* = 10 (83%)
Urological	Westermann et al. [65]	Urinary tract infections: 5 episodes in n = 2 patients (14%)
	Robins et al. [68]	Urinary tract infections: *n* = 2 (9%)
	Robins et al. [69]	Urinary tract infections: *n* = 1 (13%)
	Kraybill et al. [72]	Blood in urine: *n* = 7, most likely related to Foley catheter insertion (77%)
Diarrhoea	Westermann et al. [65]	(grades 1 or 2): 3 episodes in *n* = 2 patients (14%)
	Robins et al. [70]	(grades 1 and 2): 2 episodes (7%)
	Bull et al. [88]	*n* = 5 (36%)
	Barlogie et al. [73]	(mild to moderate): *n* = 6 (50%)
Neurological	Westermann et al. [65]	Not arousable for a period of 6 h post-WBH: *n* = 1 (7%)
	Westermann et al. [66]	Paradoxical excitation with sedation protocol (could not be treated): *n* = 1 (1%)
	Atmaca et al. [59]	Hallucination: (grade 1 and 2): 4%. Psycho-motoric dysfunction: (grade 1 and 2): 4%
	Robins et al. [67]	Pretreatment activity levels within 6 h of treatment conclusion
	Bull et al. [88]	Peripheral neuropathy: *n* = 4 (29%) (severe bilateral leg weakness with slowed conduction in femoral and peroneal nerves: *n* = 1 (7%), bilateral paresthesia and weakness of muscles innervated by the ulnar nerve: *n* = 1 (7%), footdrop and bilateral paresthesia recurred after 1 h at 41.8 °C with slowing of conduction of bilateral peroneal nerves: *n* = 1 (7%), isolated left peroneal nerve palsy with paresthesia and mild footdrop after 2 h at 41.8 °C with slowed peroneal nerve conduction velocity: *n* = 1 (7%))
	Barlogie et al. [73]	Peripheral neuropathy manifest after WBH alone: *n* = 3 (25%). Severe rhabdomyolysis: *n* = 1 (8%). EEG (electroencephalography): slowing with temperature > 40 °C and seizure activity: *n* = 2 (17%). Convulsions: *n* = 2 (17%) (without central nervous system metastases)
Cardiac	Atmaca et al. [59]	Cardiac: (grade 1 and 2): 47%, (grade 3 and 4): 2%: (AV-block (atrioventricular block): (grade 1 and 2): 28%. Ventricular extra-systole: (grade 1 and 2): 4%. Sinus arrhythmia: (grade 1 and 2): 4%. Ventricular tachycardia: (grade 1 and 2): 2%, (grade 3 and 4): 2%, ST-segment depression: (grade 1 and 2): 9%)
	Bakhshandeh-Bath et al. [60]	Cardiac arrhythmia despite use of prophylactic lidocaine during WBH: *n* = 2 (7%)
	Robins et al. [68]	Uncoupled premature ventricular contractions: *n* = 3 (13%) (disappeared with lidocaine)
	Bull et al. [88]	Arrhythmic episode: *n* = 1 (7%) (unifocal ventricular premature beats and ventricular bigeminy, 6 h after cooling)
	Gerad et al. [74]	Occasional premature ventricular contractions, supraventricular or ventricular tachycardia during WBH: 20%
Nausea/ vomiting	Robins et al. [67]	Single emesis (3–5 h post-WBH): *n* = 3 (6%) (due to gastric stasis → iv. Metoclopramide after WBH eliminated it)
	Robins et al. [68]	Vomiting and minimal nausea: *n* = 5 (22%) (3 h post-WBH, related to thiopental, subsided within 12 h post WBH)
	Robins et al. [69]	Vomiting (grade 1 and 2, related to thiopental): *n* = 6 (75%)
	Bull et al. [88]	Nausea: *n* = 7 (50%), (< 4 h after procedure)
	Barlogie et al. [73]	Nausea and or vomiting: *n* = 5 (42%)
Fatigue	Robins et al. [68]	Fatigue and lethargy: *n* = 5 (22%) (cleared at 24–48 h)
	Robins et al. [69]	(grade 1 and 2): *n* = 6 (75%)
	Bull et al. [88]	*n* = 2 (14%)
	Barlogie et al. [73]	General weakness and fatigue, 48 h after WBH: *n* = 4 (33%)
	Gerad et al. [74]	All patients after WBH, *n* = 11 (100%)
Hepatic dysfunction	Robins et al. [70]	Minor liver function test elevations and right upper quadrant pain post-WBH: *n* = 1 episode (3%)
	Kraybill et al. [72]	WBH no effect on AP (alkaline phosphatase) or other liver function tests. Many patients elevated levels of AP prior treatment. No evidence of exacerbation of liver function abnormalities secondary to hyperthermia
Renal dysfunction	Atmaca et al. [59]	Oligo-/anuria: (grade 1 and 2): 9%. Renal failure: (grade 1 and 2): 32%
	Kraybill et al. [72]	No adverse effect on renal function (exception of *n* = 1 (11%), grade 1 toxicity), all creatinine values either normal
Haemodynamic	Westermann et al. [66]	Hypotension at start of WBH (was not treated): *n* = 1 (1%)
	Atmaca et al. [59]	Hypertension: (grade 1 and 2): 2%, no pulmonary complications
	Kraybill et al. [72]	No adverse effect on BP (blood pressure) or pulse. Adequate urine output maintained with fluid resuscitation (varied from hour-to-hour in individual patients and from patient-to-patient)
	Barlogie et al. [73]	Fluid challenge well tolerated without signs of fluid overload or pulmonary oedema
	Gerad et al. [74]	Anasarca: all patients after WBH
Hiccups	Robins et al. [68]	*n* = 1 (4%)
Heel discomfort	Robins et al. [68]	*n* = 2 (9%)
Thrombophlebitis	Robins et al. [68]	Calf thrombophlebitis: *n* = 1 (4%), resolved on heparin therapy
Haematologic	Bakhshandeh-Bath et al. [60]	No differences in blood count nadirs for WBH + CTx as compared to reports of patients treated with CTx alone
Multiple organ dysfunction	Pereira Arias et al. [96]	*n* = 1 “WBH should be added as a new cause to the already known list of physical–chemical insults which can result in multiple organ dysfunction syndrome.”
Further	Atmaca et al. [59]	No treatment related deaths. Events resolved spontaneously after WBH
	Robins et al. [67]	No significant toxicity in association with WBH. No changes in cardiovascular, respiratory, haematological, biochemical indices requiring clinical intervention
	Robins et al. [68]	In no instance addition of WBH to CTx increased grade of toxicity. No acute toxicity observed during WBH
	Kraybill et al. [72]	Group: 39–39.5 °C for 3 h (*n* = 3): no adverse events. Other groups: all toxicities resolved totally within 24 h of completion of treatment
*Related to WBH and /or CTx (no further distinction by the authors)*		
Haematologic	Westermann et al. [65]	Neutropenia: (grade 1): 16%, (grade 2): 34%, (grade 3): 18%, (grade 4): 14%. Thrombocytopenia: (grade 1): 7%, (grade 2): 18%, (grade 3): 25%, (grade 4): 25%. Anaemia: (grade 1): 11%, (grade 2): 39%, (grade 3): 14%, (grade 4): 9%
	Westermann et al. [66]	Leucopenia: (grade 1): 1%, (grade 2): 7.3%, (grade 3): 22.6%, (grade 4): 57.1%. Thrombocytopenia: (grade 1): 2.7%, (grade 2): 11.5%, (grade 3): 16.7%, (grade 4): 43.9%. Anaemia: (grade 1): 14.9%, (grade 2): 28.6%, (grade 3): 8.7%, (grade 4): 5.9%
	Richel et al. [64]	Leucopenia: (grade 0): 15.9%, (grade 1): 11%, (grade 2): 37.8%, (grade 3): 29.3%, (grade 4): 6.1%. Thrombocytopenia: (grade 0): 25.6%, (grade 1): 2.4%, (grade 2): 11.0%, (grade 3): 22.0%, (grade 4): 39.0%. Neutropenia: (grade 0): 17.1%, (grade 1): 13.4%, (grade 2): 25.6%, (grade 3): 22%, (grade 4): 22%. Anaemia: (grade 0): 6.1%, (grade 1): 25.6%, (grade 2): 46.3%, (grade 3): 18.3%, (grade 4): 3.7%. Major bleeding episodes: did not occur. Neutropenic fever: 7 episodes in *n* = 5
	Bruns et al. [61]	Neutropenia: (grade 1): 8%, (grade 2): 4%, (grade 3): 24%, (grade 4): 50%. Thrombocytopenia: (grade 1): 18%, (grade 2): 22%, (grade 3): 15%, (grade 4): 18%. Anaemia: (grade 1): 22%, (grade 2): 35%, (grade 3): 9%, (grade 4): 1%
	Bakhshandeh-Bath et al. [62]	Neutropenia: (grade 1): 23%, (grade 2): 15%. Thrombocytopenia: (grade 1): 8%, (grade 2): 15%. Anemia: (grade 1): 23%. Haematologic: (grade 2): 38%
	Barlogie et al. [73]	Median lowest recorded count: platelets: 125 × 10^3^ /µl on day 12, WBC (white blood cells): 3.3 × 10^3^ /µl on day 8, granulocytes: 2.0 × 10^3^ /µl on day 8
Gastro-intestinal	Westermann et al. [65]	Nausea/vomiting: (grade 3): 2%, (grade 4): 2%. Diarrhoea: (grade 1): 2%, (grade 2): 5%. Weight loss: (grade 2): 2%. Dehydration: (grade 1): 2%, (grade 2): 2%
	Westermann et al. [66]	Gastro-intestinal: (grade 1): 11.1%, (grade 2): 6.6%, (grade 3): 0.7%. Nausea: (grade 1): 6%, (grade 2): 3.8%, (grade 3): 2%. Vomiting (grade 1): 5.6%, (grade 2): 4.2%, (grade 3): 0.3%
	Richel et al. [65]	Nausea: (grade 0): 62.2%, (grade 1): 19.5%, (grade 2): 13.4%, (grade 3): 3.7%. Vomiting: (grade 0): 63.4%, (grade 1): 22.0%, (grade 2): 9.8%, (grade 3): 2.4%, (grade 4): 1.2%. Diarrhoea (grade 1): 3 episodes in *n* = 3. Constipations: 7 episodes in *n* = 3. Dehydration: *n* = 2
	Bruns et al. [61]	Gastro-intestinal: (grade 1): 13%, (grade 2): 8%, (grade 3): 1%. Nausea: (grade 1): 51%, (grade 2): 8%, (grade 3): 9%. Vomiting: (grade 1): 41%, (grade 2): 4%, (grade 3): 4%
	Bakhshandeh-Bath et al. [60]	Gastro-intestinal: (grade 1): 13%, (grade 2): 8%, (grade 3): 1%. Nausea: (grade 1): 51%, (grade 2): 8%, (grade 3): 9%. Vomiting: (grade 1): 41%, (grade 2): 4%, (grade 3): 4%
	Bakhshandeh-Bath et al. [62]	Gastro-intestinal: (grade 1): 8%. Emesis: (grade 1): 38%, (grade 2): 8%. Vomiting: (grade 1): 31%
	Robins et al. [70]	Nausea: (grade 1): *n* = 11 episodes (37%), (grade 2): *n* = 7 episodes (23%), (grade 3): *n* = 2 episodes (7%), (grade 4): *n* = 1 episode (3%). Vomiting: (grade 1): *n* = 9 episodes (30%), (grade 2): *n* = 8 episodes (27%), (grade 3): *n* = 4 episodes (13%), no vomiting observed after implementation of ondansetron
	Gerad et al. [74]	Nausea and vomiting: in 66% of treatments (lasting about 24 h). Diarrhoea: *n* = 9 (81%)
Hepatic dysfunctions	Westermann et al. [66]	(grade 1): 5.9%, (grade 2): 10.8%, (grade 3): 1.4%
	Bakhshandeh-Bath et al. [60]	(grade 1): 14%, (grade 2): 3%
Renal dysfunction	Westermann et al. [66]	(grade 1): 3.5%, (grade 2): 0.7%, (grade 3): 0.3%, (grade 4): 0.7%
	Richel et al. [64]	(grade 0): 78%, (grade 1): 13.4%, (grade 2): 6.1%, (grade 3): 1.2%, (grade 4): 1.2%. Excessive renal toxicity: *n* = 2
	Bakhshandeh-Bath et al. [60]	(grade 1): 8%, (grade 2): 3%
	Bakhshandeh-Bath et al. [62]	(grade 1): 8%
Fatigue	Westermann et al. [66]	(grade 1): 0.3%, (grade 2): 0.7%
	Richel et al. [64]	(grade 0): 39.0%, (grade 1): 30.5%, (grade 2): 23.2%, (grade 3): 6.1%
	Bakhshandeh-Bath et al. [60]	(grade 1): 4%
	Gerad [74]	Seizure like activity during first treatment: *n* = 1
Pain	Westermann et al. [66]	(grade 1): 0.7%
	Bakhshandeh-Bath et al. [62]	(grade 2): 31%. Only *n* = 4 (31%) agreed to > 2 cycles (patients progressive or considered treatment as too demanding)
Infection	Westermann et al. [66]	Infection: (grade 1): 1%, (grade 2): 0.3%, (grade 3): 1.4%, (grade 4): 1% Deaths, associated with ureteral obstruction and sepsis: *n* = 2 → excluding patients with ureteral obstruction or stents from study entry
	Richel et al. [64]	Urinary tract infections: 4 infections in *n* = 3
	Bakhshandeh-Bath et al. [60]	Infection: (grade 1): 10%, (grade 2): 6%, (grade 3): 5%. Death associated with PD and sepsis: *n* = 1
	Gerad et al. [74]	Fever: *n* = 2 (> 38 °C at 24 h post-WBH, due to tumour and tumour-related thrombophlebitis). Granulocytopenia and fever: *n* = 4 (due to Pseudomonas aeruginosa bacteremia, urinary tract infection, transfusion reaction and respiratory infection)
Neurological	Richel et al. [64]	Neuropathy: (grade 0): 87.8%, (grade 1): 11.0%. Somnolence, recovered within 6 h: *n* = 1 (4%). Wrist drop: *n* = 1 (4%)
	Gerad et al. [74]	Myalgias: 20%. Transient paresthesia: 20%
Psychological	Richel et al. [64]	Development of a previously undiagnosed mood disorder, attempted to commit suicide: *n* = 1 (4%)
Cardiac	Bruns et al. [61]	Treatment interrupted due to cardiac arrhythmias: *n* = 2 (7%)
Alopecia	Richel et al. [64]	*n* = 1 (4%)
*Related to ECC-WBH:*		
Death	Koga et al. [75]	Probably ascribable to ECC-WBH: *n* = 4 (24%) (intra-abdominal bleeding: *n* = 1 (6%), lung oedema: *n* = 1 (6%), hepatorenal syndrome: *n* = 2 (12%))
Hepatic dysfunction	Worel et al. [91]	Transient liver failure (grade 2) resolved within 10 days after conservative management: *n* = 1 (8%)
	Wiedemann et al. [77]	Hepatitis (grade 1): *n* = 3 (25%)
Infection	Locker et al. [89]	Herpes labialis: (grade 1): 17%, (grade 2): 8% (→Famciclovir 500 mg for 5 days, postprocedural prophylaxis in patients with history of herpes virus infection)
	Koga et al. [75]	Infection of A-V (atriovenous) shunt, necessitated removal of graft: *n* = 1
	Wiedemann et al. [76]	Perioral herpes simplex: *n* = 8 (42%), resolved without specific treatment, only noted after first WBH treatment
	Wiedemann et al. [77]	Perioral herpes simplex: *n* = 3 (25%)
Fatigue	Locker et al. [89]	(grade 1): 17%, (grade 2): 58%
	Steinhart et al. [78]	Fleeting fatigue: *n* = ni
Coagulation	Locker et al. [89]	DIC (disseminated intravascular coagulation): (grade 2): 8%, PTT (partial thromboplastin time) prolongation: (grade 1): 25%
	Worel et al. [91]	Induction of hyperthermia: thrombocytopenia, increased fibrin degradation products, prolonged clotting times, alteration in coagulation factors
	Koga et al. [75]	Thrombosis in graft: *n* = 1 (6%)
	Wiedemann et al. [77]	Episodes of bleeding: *n* = 3
Renal	Locker et al. [89]	Proteinuria: (grade 1): 33%, (grade 2): 8%, (grade 3): 8%
Skin lesions	Locker et al. [89]	Burn (grade 1): 8%
	Steinhart et al. [78]	Skin imprints from heating pad: *n* = 1 (17%). Heel blisters: *n* = 2 (33%)
	Wiedemann et al. [76]	Pressure scores at contact points with blankets: *n* = 2 (11%)
	Wiedemann et al. [77]	Pressure scores (grade 3): *n* = 3 (25%)
Gastro-intestinal	Locker et al. [89]	Nausea: (grade 1): 8%. Diarrhoea: (grade 1): 17%, (grade 2): 33%
	Wiedemann et al. [76]	Diarrhoea (grade 1): *n* = 19 (100%)
	Wiedemann et al. [77]	Diarrhoea: *n* = 12 (100%). Nausea, vomiting (grade 1 or 2): *n* = 12 (100%)
Hemodynamic/Cardiac	Locker et al. [89]	Hypotension: (grade 2): 8%. No evidence of cardiovascular side effects such as arrhythmia or congestive heart failure
	Wiedemann et al. [76]	Mild anasarca: after every WBH treatment. Lung oedema: *n* = 2 (11%) (WBH had to be discontinued)
	Wiedemann et al. [77]	Mild anasarca: *n* = 12. Ventricular arrhythmias: *n* = 3
Neurological	Koga et al. [75]	Weakness of muscles in lower extremities (disappeared within about 1 month after treatment): (severe, drop foot): *n* = 3 (17%), (moderate): *n* = 4 (24%), (slight): *n* = 5 (29%)
	Steinhart et al. [78]	Severe encephalopathy: *n* = 1 (17%) (expired 3 weeks later, according to the authors: death not attributed to treatment)
	Wiedemann et al. [77]	Reversible paresthesia (grade 2) of hands and feet: *n* = 2 (17%)
Fever	Locker et al. [89]	(grade 1): 33%
Cough	Locker et al. [89]	(grade 1): 17%
Catheter related	Locker et al. [89]	Placement of pulmonary artery catheter resulted in intravascular burling, requiring surgical removal: *n* = 1 (17%)
Pain	Steinhart et al. [78]	Mild muscle soreness. Tenderness over vascular access sites, *n* = ni
*Related to ECC-WBH and/or CTx (no further distinction by the authors):*		
Haematologic	Koga [75]	Thrombocytopenia: *n* = 13 (76%) (1–3 days after treatment). Leucopenia: *n* = 8 (47%), time to leucocyte count nadirs not uniform
	Wiedemann et al. [77]	Thrombocytopenia (grade 3 or 4): *n* = 7 (58%), (grade 1 or 2): *n* = 4 (33%). Leucopenia (grade 3 or 4): *n* = 12 (100%). Anaemia (grade 3 or 4): *n* = 4 (33%), (grade 1 or 2): *n* = 8 (67%)
	Feyerabend et al. [97]	Thrombocytopenia (grade 4): *n* = 1
Renal dysfunction	Wiedemann et al. [76]	(mild): *n* = 4 (21%), (severe): *n* = 2 (11%) → acute renal failure, required haemodialysis
	Wiedemann et al. [77]	Elevated creatines values: *n* = 12 (100%), signs of nephrotoxicity: *n* = 5 (42%), severe renal toxicity: *n* = 1 (8%) → acute renal failure, required haemodialysis
Neurological	Wiedemann et al. [76]	Severe objective sensory loss and weakness with impairment of function: *n* = 1 (5%). Somnolence: *n* = 1 (5%)
	Feyerabend et al. [97]	Tolerated well, no neurologic sequelae occurred. Polyneuropathy. “WBH at 41.8 °C must not be used primarily in patients with cerebral or spinal metastases because of the risk of a deleterious increase of intracranial or intraspinal pressure.”
Fatigue	Wiedemann et al. [76]	*n* = 19 (100%)
Nausea/vomiting	Wiedemann et al. [76]	Nausea or vomiting well controlled with antiemetics
*Related to EH:*		
Pain	Wismeth et al. [81]	Local pain: (grade 1): *n* = 3 (20%), (grade 2): *n* = 9 (60%)
	Yu et al. (EH + RTx) [83]	10% energy reduction needed: *n* = 2 (20%) (EH related pain). Refusal of further EH-session after third and fourth sessions (EH related pain): *n* = 2 (20%)
Headache	Wismeth et al. [81]	(grade 2): *n* = 7 (47%), (grade 3): *n* = 5 (33%), (grade 4): *n* = 1 (7%)
Neurological	Wismeth et al. [81]	Increased intracranial pressure (→ corticosteroids or mannitol standard to resolve/prevent side effects). Nausea: (grade 1): *n* = 3 (20%), (grade 2): *n* = 3 (20%). Vomiting: (grade 1): *n* = 2 (13%), (grade 2): *n* = 6 (40%). Confusion: (grade 1): n = 1 (6%), (grade 2): *n* = 4 (27%), (grade 3): *n* = 3 (20%). Slowed psychomotor function: (grade 2): *n* = 1 (7%). Dizziness: (grade 1): *n* = 4 (27%), (grade 2): *n* = 2 (13%). Somnolence: (grade 2): *n* = 1 (7%), (grade 3): *n* = 2 (13%). Focal neurological symptoms: Hemiparesis: (grade 2): *n* = 7 (47%), (grade 3): *n* = 5 (33%). Cranial nerve dysfunction: (grade 1): *n* = 3 (20%), (grade 2): n = 2 (13%), (grade 3): *n* = 1 (7%). Aphasia: (grade 3): *n* = 5 (33%), (grade 4): *n* = 1 (7%). Seizures: (grade 2): *n* = 5 (33%). Hydrocephalus: (grade 3): *n* = 1 (7%)
Skin	Jeung et al. [94]	In negligible cases: skin erythema, handled with appropriate cream → did not terminate further treatments
Further (according the authors)	Jeung et al. [94]	In four cases: comments regarding side effects (all of them describes good compatibility). In the other cases: no information concerning side effects. No adverse effect originated from EH. Any negative side effects did not appear even though high temperature been expected at metal stent site. Metal stent in the canal or duct: no absolute contraindication for EH
Further	Wismeth et al. [81]	Reasons for patients, dose could not fully escalated: subjective overheating, headache, other signs of intracranial pressure. Didn’t reach minimum protocol dose: *n* = 2 (13%)
*Related to EH and/or CTx (no further distinction by the authors):*		
Haematologic	Sahinbas et al. [84]	Leucopenia: (grade 1): *n* = 1 (25%), (grade 2): *n* = 3 (75%). Thrombocytopenia: (grade 1): *n* = 2 (50%), (grade 2): *n* = 1 (25%), (grade 3): *n* = 1 (25%). Anaemia: (grade 1): *n* = 1 (25%), (grade 2): *n* = 2 (50%), (grade 3): *n* = 1 (25%). Granulocytopenia: (grade 2): *n* = 3 (75%)
Hepatic dysfunction	Sahinbas et al. [84]	Bilirubin elevation: (grade 3): *n* = 1 (25%). GOT (glutamate oxaloacetate transaminase) elevation: (grade 1): *n* = 2 (50%), (grade 2): *n* = 1 (25%). GPT (glutamate pyruvate transaminase) elevation: (grade 1): *n* = 3 (75%). AP (alkaline phosphatase) elevation: (grade 2): *n* = 2 (50%), (grade 3): *n* = 1 (25%)
Gastro-intestinal	Sahinbas et al. [84]	Nausea: (grade 1): *n* = 1 (25%)
	Gadaleta-Caldarola et al. [80]	Vomiting (grade 1 or 2): 10%, diarrhoea (grade 3): 5%, anorexia (grade 1 or 2): 25%
Skin lesions	Gadaleta-Caldarola et al. [80]	Hyperemia (grade 1 or 2): 20%, hand foot skin reaction (grade 3): 10%
Alopecia	Sahinbas et al. [84]	(grade 1): *n* = 1 (25%)
Fatigue	Gadaleta-Caldarola et al. [80]	(grade 3): 5%
Hypertension	Gadaleta-Caldarola et al. [80]	(grade 3): 5%
*Related to EH and/or RTx (no further distinction by the authors):*		
Haematologic	Heo et al. [86]	Anaemia: (grade 1): 5%, (grade 2): 30%. Leucopenia: (grade 1): 5%, (grade 2): 5%
Gastro-intestinal	Heo et al. [86]	Nausea: (grade 2): 15%. Vomiting: (grade 1): 5%
Headache	Heo et al. [86]	Dizziness: (grade 1): 5%, (grade 2): 5%. Headache: (grade 2): 10%
Skin lesions	Heo et al. [86]	Skin burn: (grade 2): 5%
Odynophagia	Yeo et al. [95]	Mild, subsided with conservative management
*Related to CTx:*		
Haematologic	Atmaca et al. [59]	Leucopenia: (grade 1 and 2): 15%, (grade 3 and 4): 49%. Thrombocytopenia: (grade 1 and 2): 6%, (grade 3 and 4): 65%. Anaemia: (grade 1 and 2): 28%, (grade 3 and 4): 49%
	Bakhshandeh-Bath et al. [60]	Neutropenia: (grade 1): 8%, (grade 2): 4%, (grade 3): 24%, (grade 4): 50%. Thrombocytopenia: (grade 1): 18%, (grade 2): 22%, (grade 3): 15%, (grade 4): 18%. Aanemia: (grade 1): 22%, (grade 2) 35%, (grade 3): 9%, (grade 4): 1% →No differences in blood count nadirs for WBH + CTx as compared to reports of patients treated with CTx alone
	Robins et al. [70]	Myelosuppression: major toxicity
	Bull et al. [71]	Leucopenia: (grade 1): 31%, (grade 2): 55%. Thrombocytopenia: (grade 1): 62%, (grade 2): 21%. Anaemia: (grade 1): 52%, (grade 2): 21%
	Wiedemann et al. [76]	Myelosuppression major toxicity. Anaemia (grade 1): *n* = 18 (95%), (grade 2): *n* = 7 (37%)
	Wismeth et al. [81]	Leucopenia: (grade 1): *n* = 1, (grade 2): *n* = 5, (grade 3): *n* = 2. Febrile neutropenia: (grade 3): *n* = 1. Thrombocytopenia: (grade 1): *n* = 2, (grade 2): *n* = 3, (grade 3): *n* = 2, (grade 4): *n* = 1
	Douwes et al. [79]	Leucopenia: (grade 2): *n* = 6 (20%), (grade 3): *n* = 2 (7%). Thrombocytopenia: (grade 2): *n* = 4 (13%). Anemia: (grade 2): *n* = 13 (43%), (grade 3): *n* = 3 (10%)
Hepatic dysfunction	Atmaca et al. [59]	Hepatic failure: (grade 1 and 2): 10%
Neurological	Atmaca et al. [59]	Neuro-sensory failure: (grade 1 and 2): 11%
	Robins et al. [68]	Myalgias (mild): *n* = 10 (59%), (moderate): *n* = 2 (12%), (severe): *n* = 1 (6%). Photophobia (mild): *n* = 5 (29%)
	Bull et al. [71]	Neuropathy: (grade 1): 7%, (grade 2): 3%
Gastro-intestinal	Atmaca et al. [59]	Nausea: (grade 1 and 2): 45%, (grade 3 and 4): 2%. Diarrhoea: (grade 1 and 2): 2%. Constipation: (grade 1 and 2): 2%
	Robins et al. [68]	Diarrhoea: (mild): *n* = 2 (12%), (moderate): *n* = 2 (12%)
	Bull et al. [71]	Nausea: (grade 1): 17%, (grade 2): 3%
Pain	Robins et al. [68]	Testicular pain (mild): *n* = 3 (18%), (moderate): *n* = 2 (12%). Nipple tenderness (mild): *n* = 2 (12%)
Infection	Atmaca et al. [59]	Infection: (grade 1 and 2): 4%, (grade 3 and 4): 11%. Herpes: (grade 1 and 2): 20%, (grade 3 and 4): 4%
	Robins et al. [68]	Urinary frequency (mild): *n* = 2 (12%), (moderate): *n* = 4 (24%), (severe): *n* = 1 (6%)
Mucositis	Atmaca et al. [59]	Mucositis: (grade 1 and 2): 6%
Further	Robins et al. [68]	Alopecia (moderate): *n* = 1 (6%). Ototoxicity (mild): *n* = 5 (29%), (moderate): *n* = 1 (6%)
*Related to RTx:*		
Pneumonitis	Yeo et al. [95]	Signs of radiation pneumonitis and fibrosis around treated region, with no specific associated symptoms

*CTx* chemotherapy; *ECC-WBH* WBH with extracorporeal circulation; *EH* electro hyperthermia; *h* hour; *n* number of patients; *RTx* radiotherapy; *WBH* whole-body-hyperthermia

**Table 7:** Characteristics of studies with multiple intervention

Reference	Study type	Type of cancer	Multiple interventions
Aschhoff et al. [98]	Single-arm	Prostate cancer	EH + Ukrain
Bremer et al. [99]	Single-arm	Breast-, ovarian-, and colorectal cancer	WBH + CTx + hyperoxemia + hyperglycaemia
DeCesaris et al. [100]	Case report	Cutaneous squamous cell carcinoma	Cetuximab + RTx + EH
Douwes et al. [101]	Single-arm	Ovarian cancer	WBH + CTx + hyperglycaemia
Fiorentini et al. [102]	Cohort study	Pancreatic cancer	Group A: EH (*n* = 39), subgroup A1: EH + CTX (*n* = 32), subgroup A2: EH alone: (*n* = 7) Group B: no EH (*n* = 67), subgroup B1: CTx: (*n* = 36), subgroup B2: supportive care (vitamins, analgesics, parenteral nutrition, acupuncture, and phytotherapy) (*n* = 31) (Exclusion, because no demographic data for the subgroups)
Hager et al. [103]	Single-arm	Colorectal cancer	EH + unspecific immunotherapy: thymus peptides, lectins and enzymes
Hager et al. [104]	Case series	Pancreatic carcinoma	EH + complementary treatments
Hildebrandt et al. [105]	Case report	Germ cell tumor	WBH + CTx + hyperoxemia + hyperglycaemia
Hildebrandt et al. [106]	Unclear	Metastatic colorectal cancer	WBH + CTx + hyperoxemia + hyperglycaemia
Holzhauer et al. [107]	Case report	Bone and hepatic metastasizing prostate cancer	EH + CTx + several other complementary methods
Iyikesici et al. [108]	Single-arm	Non-small cell lung cancer	CTx + ketogenic diet + EH + hyperbaric oxygen therapy
Iyikesici et al. [109]	Single-arm	Gastric cancer	CTx + ketogenic diet + EH + hyperbaric oxygen therapy
Iyikesici et al. [110]	Single-arm	Metastatic pancreatic cancer	CTx + hyperbaric oxygen therapy + EH
Jun et al. [111]	Single-arm	Different entities of cancer	EH + Gun-Chil Jung capsule
Kerner et al. [112]	Single-arm	Colorectal cancer, germ cell tumour, ovarian cancer, cervix cancer, lymphoma	WBH + CTx + hyperoxemia + hyperglycaemia
Kleef et al. [113]	Case report	Ovarian cancer	EH + CTx + dendritic cells
Kleef et al. [114]	Case series	Different entities of cancer	Ipilimumab + nivolumab + Interleucin-2 + EH or WBH + high dose vitamin C intravenously + alpha lipoic acid
Ko et al. [115]	Cohort study	Different entities of cancer	EH + RTx + different combination of surgery, immunotherapy, CTx, hormone therapy (not comparable)
Koike et al. [116]	Single-arm	Metastatic lymph nodes of oral squamous cell carcinoma	Retrograde superselective intra-arterial CTx + surgery + EH
Krasny et al. [117]	Cohort	Renal carcinoma	Different combinations of WBH + surgery + CTx + hyperglycaemia (not comparable)
Lee et al. [118]	Case report	Breast leiomyosarcoma	EH + Pazopanib
Nagata et al. (2021) [119]	Case series	Breast cancer	Different combinations of: hormonal therapy, RTx, surgery, various CTx, targeted molecular treatment, EH
Nozato et al. [120]	Single-arm	Metastatic lymph nodes of squamous cell carcinoma	Retrograde superselective intra-arterial CTx + EH + surgery
Ou et al. [121]	Single-arm	Non-small cell lung cancer	EH + intravenous ascorbic acid
Ou et al. [122]	RCT	Non-small lung cancer	Arm A: intravenous Vit C + EH + best supportive care Arm B: best supportive care alone
Pang et al. [123]	RCT	Peritoneal carcinomatosis with malignant ascites	Group A: EH + TCM (traditional Chinese medicine), n = 130 Group B: intraperitoneal CTx, *n* = 130
Qiao et al. [124]	Cohort study	Different entities of cancer	Different combination of autologous adoptive cell therapy + CTx + anti-PD-1 antibody + EH
Ranieri et al. [125]	Single-arm	Metastatic Colon Cancer	Bevacizumab + CTx + EH
Robins et al. [126]	Single-arm	Different entities of cancer	WBH + IFN (interferon)
Robins et al. [127]	Single-arm	Different entities of cancer	WBH + CTx + TNF α (tumour-necrosis-factor)
Roussakow et al. [128]	Retrospective cohort	Glioma	Different combinations of EH + supportive treatments: mistletoe, selenium, boswellia caterii (not comparable)
Rubovszky et al. [129]	Case report	Non-small cell lung cancer	WBH + CTx + Bevacizumab
Sahinbas et al. [130]	Case series	Glioma	EH + CTx + RTx + supportive treatments: mistletoe, selenium, boswellia caterii
Sakuma et al. [131]	Case report	Carcinoma of the buccal mucosa	Superselective intra-arterial CTx + EH + CTx + cetuximab
Sawai et al. [132]	Case report	Breast cancer	CTx + RTx + EH + hormone therapy
Scheer et al. [133]	Single-arm	Desmoplastic small round cell tumours	Different combinations of CTx, RTx, autologous stem cell rescue, EH, HIPEC (hyperthermic intraperitoneal CTx)
Schencking et al. [134]	Case report	Metastases of breast cancer in lumbar spine	WBH + analgesics + several other therapies
Wehner et al. [135]	Single-arm	Different entities of cancer	WBH + CTx + hyperoxemia + hyperglycaemia
Weingartner et al. [136]	Case series	Head and neck squamous cell carcinoma	RTx + some patients CTx + two patients cetuximab or nivolumab
Wust et al. [137]	Single-arm	Metastatic colorectal cancer	WBH + CTx + hyperoxemia + hyperglycaemia
Yu et al. [138]	Single-arm	Hepatocellular carcinoma	EH + RTx + TACE (transcatheter arterial chemoembolization)
Yu et al. [139]	Single-arm	Hepatocellular carcinoma	EH + RTx + TACE (transcatheter arterial chemoembolization)
Zheng et al. [140]	Case report	Ovarian cancer	Poly-ADP-ribose-polymerase + CTx + EH

*CTx* chemotherapy; *ECC-WBH* WBH with extracorporeal circulation; *EH* electro hyperthermia; *h* hour; *n* number of patients; *RCT* randomized controlled trial; *RTx* radiotherapy; *WBH* whole-body-hyperthermia

**Table 8:** Adverse events related to hyperthermia treatment of studies with multiple interventions

Side Effect	Reference	Specific/Grade (*n*)
*Related to WBH:*		
Skin lesions	Wehner et al. [135]	(grade 1, erythema): *n* = 20 (25%), disappeared after the 2.-3. day post-WBH, (grade 2, skin areas with a mean of 14.4 cm, range: 0.1–70 cm^2^): *n* = 15 (19%), (grade 3, area of 8,8 cm^2^): *n* = 1 (1%)
	Bremer et al. [99]	(grade 0): *n* = 10 (53%), (grade 1): *n* = 5 (26%), (grade 2): *n* = 3 (16%), (grade 3): *n* = 1 (5%)
	Wust et al. [137]	(grade 0, temporal erythema): 30%, (grade 1, erythema or pain, > 1 day): 20%, (grade 2, superficial burns): 30%, (grade 3, deep tissue lesions): 20%
	Douwes et al. [101]	Mild, skin reactions, mild burn (grade 1): *n* = ni
	Hildebrandt et al. [105]	Thermic skin lesions
Herpes infection	Robins et al. [127]	*N* = 3 episodes (15%), resolved with acyclovir
	Robins et al. [126]	Self-limiting: *n* = 4 (24%)
	Wust et al. [137]	(grade 1): 30%, (grade 2): 5%
	Bremer et al. [99]	Labial: (grade 0): *n* = 10 (53%), (grade 1): *n* = 7 (37%), (grade 2): *n* = 2 (11%)
Haematologic	Wehner et al. [135]	Treatments without CTx (*n* = 14): no haematotoxic side effects
Neurological	Kerner et al. [112]	Slight CNS dysfunction for < = 12 h: disorientation and a GCS of 12–14 → GCS returned to 15 spontaneously: *n* = 4 (18%). Encephalopathy (agitation and disorientation): *n* = 2 (9%) (reversed spontaneously after 6 days, 12 h) *n* = 1: due to brain metastasis)
	Wust et al. [137]	Neuropathy (grade 0 and 1): 70%, (grade 2): 15%, (grade 3 and 4): 15%. Mild disorientation: (< 12 h): 10%, (3–6 d): 10%. Transitional psychosis: 5%. Encephalopathy (directly after WBH, lasted for 6 days, reversed spontaneously): *n* = 1
Headache	Robins et al. [126]	6 episodes (in *n* = 3, 18%)
Nausea/vomiting	Robins et al. [126]	Vomiting: *n* = 10 episodes (30%)
	Robins et al. [127]	Nausea (grade 1): *n* = 3 episodes (15%). Vomiting (grade 2): *n* = 1 episode (5%)
Fatigue	Robins et al. [126]	Moderate fatigue: *n* = 8 episodes (*n* = 4) (24%)
	Bremer et al. [99]	Fatigue/asthenia: (grade 0): *n* = 13 (68%), (grade 1): *n* = 2 (11%), (grade 2): *n* = 4 (21%)
Hepatic dysfunction	Robins et al. [127]	Transient elevation in liver function tests (grade 1): *n* = 1 episode (5%)
Haemodynamic	Wust et al. [137]	Haemodynamic depression: (grade 0): 65%, (grade 1 and 2): 5%, (grade 3): 30%
Pain	Wust et al. [137]	Persisting pain (< 24 h): 35%, (> 24 h): 5%
Cardiac	Kerner et al. [112]	Supraventricular tachyarrhythmias: *n* = 2 (10%)
	Wust et al. [137]	Supraventricular tachycardia: (grade 3): 5%
*Related to EH:*		
Skin lesions	Sahinbas et al. [130]	Local redness of skin: 8%. Subcutaneous fibrosis of fatty tissue: 1%. Skin burn (diameter < 1.5 cm) (grade 1–2): 2%
	Jun et al. [111]	Burn: (grade 1): *n* = 1 (1.8%)
	Qiao et al. [124]	Blistering *n* = 3 (9.1%). Subcutaneous fat induration: *n* = 4 (12.1%)
	Ranieri et al. [125]	Erythema: *n* = 3 (7.5%)
	Fiorentini et al. [102]	Brun: (grade 1): *n* = 6, (grade 2): *n* = 2
Pain	Yu et al. [139]	Pain: *n* = 39 (57%). No pain or an NRS pain score < 5: *n* = 11 (16%). Refusal of further EH sessions, mainly because of pain: *n* = 21 (30%) → to complete treatment, administration of opioids. Failure of planned escalation of energy to 200 W: *n* = 45 (65%). Received hyperthermia of only < = 100 W, because of pain: *n* = 23 (33%)
	Jun et al. [111]	Abdominal pain: (grade 1): *n* = 1 (1.8%). Right chest pain: *n* = 1 (1.8%)
	Qiao et al. [124]	Local heating pain: *n* = 3 (9.1%)
	Ranieri et al. [125]	Local pain: *n* = 4 (10%)
	Fiorentini et al. [102]	Skin pain: 2%
Headache	Sahinbas et al. [130]	(< 2 h): 12%
Neurological	Sahinbas et al. [130]	Short term asthenia after treatment (< 2 h): 9%
Sinus tachycardia	Qiao et al. [124]	*n* = 1 (3.0%)
Vomiting	Qiao et al. [124]	*n* = 1 (3.0%)

*cm* centimetre; *CNS* central nerve system; *CTx* chemotherapy; *EH* electro hyperthermia; *GCS* Glasgow Coma Scale; WBH with extracorporeal circulation. *h* hour; *n* number of patients; *RTx* radiotherapy; *WBH* whole-body-hyperthermia

### Characteristics of included studies

The first level of evidence included 14 studies, with one systematic review (SR), six randomized controlled trials (RCT), three controlled trials (CT) and four cohort studies with overall 1366 patients, from which 1137 were analysed, due to 229 drop-outs. The age of patients ranged from 17 to 86 years. In total, 955 participants were females, and 411 were males. In one study with 131 participants [50] data on age, gender and drop-outs were missing, whereas in the SR [45] data on age, gender, number of participants and drop-outs were missing. The publications by Minnaar et al. [51–53] used the same patient collective, but the endpoints were different, so the studies were analysed individually.

The second level of evidence consisted of 32 single-arm studies, one case series and six case reports comprising 712 patients. Of these, 332 were females and 363 were males. In one study, data on gender were missing for 17 patients [63]. The age of patients ranged from 12 to 86 years. The publications by Bruns et al. [61] and Bakhshandeh et al. [60] reported the same 27 patients. Because the focus was partially on different outcomes, these studies were analysed individually.

Overall, WBH was used in 33 studies (WBH in 24 studies [45–48, 59–74, 88, 92, 93, 96], ECC-WBH (WBH with extracorporeal circulation) in 7 studies [75–78, 89, 91, 97], in one study WBH and ECC-WBH each used for different groups [49] and in one study WBH (water-filtered infrared-A radiation) was administered together with therapeutic fevers [50]). EH was used in 20 studies [51–58, 79–87, 90, 94, 95]. Outcomes included tumour response, course of tumour markers, survival data, pain, quality of life or quality of recovery, body weight, ascites and fatigue. Further outcomes included haemodynamic, haematologic, serum chemistries, immunological and pH values, PaCO_2_ and respiratory rate. Other specific results contained measured temperatures, tumour oxygenation and heat-dose tolerances.

### Risk of Bias in included studies

The main shortcomings of the studies are summarized in Fig. 2 (first level of evidence) and Fig. 3 (second level of evidence). Among the first level of evidence seven studies have an acceptable quality, six studies have a low quality, and one study is rated as unacceptable. The single-arm studies showed a quality from low to acceptable. A detailed description of the methodological quality of each study is provided in supplementary table 1 (first level of evidence) and supplementary table 2 (second level of evidence).

### Endpoints in the first level of evidence (SR, RCTs, CTs and cohort studies)

Detailed information to the outcomes and side effects can be seen in Table 3.

#### Benefit and risks of hyperthermia in combination with systemic therapy on solid tumours (Whole-body hyperthermia)

In the SR by Lassche et al. [45] pre-treated patients with heterogeneous solid tumours were treated with WBH and CTx (chemotherapy). Among these, ovarian cancer (*n* = 3 studies), colorectal carcinoma (*n* = 2 studies), lung cancer (*n* = 2 studies) and sarcoma (*n* = 3 studies) were the most common entities analysed in this SR. In total, 13 studies included patients with various malignancies. These studies were then referred to as phase-I studies. CTx was used in all studies. These regimes were not uniform, but in most cases, platinum containing agents (*n* = 10 studies) or alkylating agents (*n* = 6 studies) were used. In the vast majority temperature reached 41.8 °C over a period of one hour and was generated by means of radiant heat. The response rate (including complete and partial response) was analysed in a total of 13 phase-I (various malignancies) and 14 phase-II (special malignancies) studies and varied between 12 and 89%. However, in the two studies with the highest response rates (86%, resp. 89%), the number of subjects was very small (*n* = 15, *n* = 18 resp.) and the pretreatment was described insufficiently. All the studies were single-arm studies. Grade 3 and grade 4 toxicities according to the CTAE criteria (Common Terminology Criteria for Adverse Events) occurred in almost all studies.

#### Recovery after surgery (Whole-body hyperthermia)

The RCT by Sulyok et al. [46] analysed the effect of WBH on the quality of recovery after curative colorectal cancer surgery (*n* = 18). The intervention group was sedated 3.5 h before the beginning of surgery and then treated with WBH by infrared radiation. The time at the target temperature of 39 °C was 2 h. Twenty-four hours after surgery, no significant difference was found for quality of recovery (QoR) overall or in quality of life (quality of recovery, global QoR-40, *p* = 0.81).

#### Effect of whole-body hyperthermia on the toxicity of chemotherapy (Whole-body hyperthermia)

The phase-II-CT by Hegewisch-Becker et al. [48] included 44 patients with adenocarcinoma of colon or rectum with progressive disease. Every second cycle of the biweekly chemotherapy regime, consisting of oxaliplatin, leucovorin and 5-fluoruracil, was combined with two hours of WBH with an estimated intra-tumoural temperature of 41.8 °C. WHO (World Health Organization)-grade three side effects of chemotherapy, including haematologic, gastrointestinal, peripheral neurological toxicities and fatigue, were rare and evenly balanced between the cycles with or without hyperthermia. Due to the crossover design, the comparability of the two groups is given, but carry-over effects cannot be ruled out.

#### Tumour response, abscopal response, survival and quality of life in cervical cancer patients (electro hyperthermia)

In the RCT by Minnaar et al. [51] 210 patients received RTx (external beam radiation) and cisplatin with or without EH. Heating reached estimated 42.5 °C and was generated via a capacitive heating device (EHY2000, Oncotherm) for two times a week. According to the authors, there was no need to measure the temperature, and the applied dose of EH should be controlled by means of the absorbed energy. Six-month local disease-free survival in the intervention group was significantly higher than in the control group (*p* = 0.003). Furthermore, a significant higher rate of complete metabolic responses was reported in the arm with the administration of EH (*p* = 0.005). A complete metabolic response as well in the primary tumour as in the lymph nodes within and outside the radiation field occurred significantly more often in the EH group (*p* = 0.013) [53]. The authors discussed that as abscopal effect which has been described in a few case reports in the literature due to radiotherapy. The quality of life (QoL) was determined using the EORTC questionnaires QLQ C30 and Cx24 6 weeks and 3 months post-treatment. Six weeks after treatment, mean change in cognitive function in the intervention group was significantly higher than in the control group (*p* = 0.031). Three months after treatment a significant improvement in social functioning (*p* = 0.049), emotional functioning (*p* = 0.017), fatigue (*p* = 0.037) and pain (*p* = 0.007) was reported in the EH group, compared to the control group [52]. No data on the target temperature in the tumour field are reported. In these studies, many calculations are performed. However, in the exact comparison of the intervention and control group regarding the therapy, these data are missing. Therefore, it is not possible to accurately compare the treatments between the two arms with and without hyperthermia. In addition, information about prior treatments is not specified and a description of possible additional co-interventions is missing. For the endpoints tumour response and local disease control, reasons for the drop-out of part of the participants are not given. Therefore, it cannot be excluded that for these endpoints only suitable patients were considered.

#### Tumour response and survival in breast cancer patients (electro hyperthermia)

In the RCT by Loboda et al. [54] 103 patients were treated with EH together with neoadjuvant CTx. The control group, containing 97 patients, received only the neoadjuvant CTx. Target temperatures were estimated to reached up to 38.8 °C and maintained for about 30 min. The intra-tumour temperature estimations were based on an unvalidated bioheat model combined with skin temperatures measured using a thermal imaging camera. Considering the individual values for the tumour response according to the RECIST criteria (Response Evaluation Criteria in Solid Tumours), no significant difference between the two groups was described. The 10-year OS in the hyperthermia group was significantly longer (*p* < 0.009). Some points must be questioned in this study. The abstract reports that the intervention group had achieved a significantly higher rate of objective responses. An objective response included patients with a partial and complete response. However, when looking at the individual values for a complete response, partial response or stable disease, no significant difference can be found. Therefore, the significant difference in the abstract has to be doubted. Moreover, no information about the method of randomization is indicated. Another point for statistical criticism concerns the 10-year OS. No exact figures are given, for example, by how much longer survival was in the intervention group. The calculation of a significant difference is thus not comprehensible and remains vague. In addition, a comparison of the altered blood flow after treatment between the two groups is lacking.

#### Survival and tumour response in patients with glioblastoma (electro hyperthermia)

In the CT by Mahdavi et al. [55] glioblastoma patients were treated with CTx and RTx. The patients in the intervention arm received additional EH with estimated 41 °C (Celsius 42 +) two times a week for one hour each. Although tumour volume was significantly lower after treatment in the intervention group (*p* < 0.05), the OS between the two arms after 18 months was not significantly different (*p* = 0.55). Furthermore, the Karnofsky performance status did not differ significantly. In total, no information about the allocation method is given. Moreover, no comparison of the patient and tumour characteristics at baseline was conducted at all and in addition with the small number of participants (*n* = 38) an unequal distribution of subjects cannot be excluded. Additionally, side effects were reported only briefly.

#### Pathologic outcome and survival in patients with rectum carcinoma (electro hyperthermia)

In the retrospective cohort study by Kim et al. [58], 120 patients with rectum carcinoma received neoadjuvant RTx and CTx. The intervention group was treated additionally with EH (EHY 2000, Oncotherm). A hyperthermia session lasted one hour, and the number of sessions varied between one and twelve. Information about the targeted temperature or the temperature measurement is not given. A comparison of the two groups was done at baseline. Regarding the pathologic outcome (near total regression and total regression), no significant differences between arms were found, except for tumours with an initial primary volume more than 65 ml (*p* = 0.024). Considering the 2-year OS (*p* = 0.73), the 2-year disease-free survival (*p* = 0.054), the 2-year locoregional recurrence-free survival (*p* = 0.09) and the 2-year distant metastases-free survival (*p* = 0.083) no significant difference was found between the two groups. It has to be noticed that the dose of RTx was different between the treatment groups. In the intervention group the RTx-dose varied between 40 and 50.4 Gy, and in the control group, all participants received 50.4 Gy. Furthermore, it is not clear how the allocation to the different treatment groups was made.

#### Tumour response and survival of glioblastoma and astrocytoma patients (electro hyperthermia)

In the retrospective cohort study by Fiorentini et al. [56] 111 glioblastoma multiforme and 38 astrocytoma patients were divided into two groups. The intervention group (*n* = 52) was treated with EH (EHY2000, Oncotherm), whereas the control group (*n* = 97) received best supportive care together with CTx. The temperature in the EH group was between 40 and 42.5 °C and was only estimated. In the subgroup of astrocytoma patients, the results show an overall positive response, including complete response, partial response and stable disease in favour of the intervention (*p* < 0.005). The same applies for the subgroup of glioblastoma patients (*p* < 0.05). Moreover, the median OS was significantly better in both subgroups compared to the controls (*p* = 0.0065; *p* = 0.047). In the control arm, different CTx-regimes were administered; in contrast, the intervention arm received no CTx at all. With regard to the methodology of the study, there are several drawbacks. The functional recovery was only measured by ECOG grading. A comparison of the baseline data between control group and intervention group is missing. Therefore, it is not clear whether both arms can be compared at all.

#### Pain relief in lung cancer (electro hyperthermia)

In this retrospective cohort study by Kim et al. [57], the intervention group was treated additionally with EH (EHY2000, Oncotherm) two to three times (estimated 39–42 °C for about 60 min). Data collection was carried out at four different time points during 180 days after the start of the study. No significant differences could be found at any time for pain intensity and effective analgesic score (EAS), while the changes of the EAS over the time distinguished in dependence of the treatment (*p* = 0.038) with worse values in the intervention group in the first 60 days (*p* = 0.030). Exactly in these days, a significantly higher opioid analgesic dose was used in the intervention group (*p* = 0.022).

It must be critically noted that at baseline only 47.4% of the initially matched control group entered the study. Owing to this high drop-out, the comparability is limited and perhaps not given anymore. Despite that, the results suggest that EH leads to more pain immediately after the treatment.

#### Effect of preceding WBH on induced therapeutic fevers

In the retrospective phase-I-CT by Reuter et al. [50], participants (*n* = 131) were allocated to three different treatment groups and the desired target temperature was about estimated 39–40 °C. In group A1, with 44 participants, the bacterial extracts Serratia marcescens + Streptococcus pyogenes or Pseudomonas aeruginosa were used. In group A2 62 patients were treated with the same bacterial extracts, preceded by 30 min WBH (IRA 1000, Von Ardenne). In group B, containing 25 participants, therapeutic fever was induced by the application of combinations of approved drugs (Colibiogen, Iscador, Picibanil, Polyvaccinum forte, Strovac) and preceded by WBH (*n* = 25). Even though the authors conclude a reduction of side effects of some bacterial extracts through the preceding treatment with hyperthermia, no statistical data are given for this conclusion.

The drawbacks of this study are low and incomplete reporting regarding basic demographic data and the allocation procedure. Furthermore, the patient collective is very heterogeneous and perhaps also selective. Therefore, we classify the methodological quality of the study as unacceptable and refrain from further discussion of the results in the following.

#### Endpoints in the second level of evidence (single-arm studies, case series and case reports)

Further information to the individual results of each study is shown in Table 4 (outcomes in single-arm and case series) and Table 5 (outcomes in case reports).

#### Endpoint tumour response, pain, quality of life and fatigue

Three single-arm studies exist, which used alternative hyperthermia alone. In these trials no clinical tumour responses were documented [67, 72, 78]. In 22 single-arm studies and in one case series, alternative hyperthermia was used in combination with CTx or RTx. The incidence of a (complete response) CR ranged from 0% in 13 studies [60–62, 67, 72, 73, 75–78, 80, 83, 84] to 37.5% in a study with B-cell neoplasm with 8 participants [69]. In summary, due to the heterogeneous tumour entities and to the fact that all single-arm studies, which reported an improvement in tumour response, used alternative hyperthermia combined with CTx or RTx, and no conclusion can be drawn, whether the addition of hyperthermia to CTx or RTx has an effect on tumour response.

Pain as outcome was analysed in five studies including 106 patients. A reduction of pain was only seen in the 13 patients with objective tumour response, after a treatment which combined WBH with other treatments. Looking at ECC-WBH as part of therapy, 18% and 21% of the 36 participants reported a reduction of abdominal cancer pain [75]. Moreover, for EH as part of therapy for 33 patients, a decrease in median VAS score was reported [83]. In 23 patients, significant reduction in worst pain, least pain, average pain and current pain (*p* < 0.001 for all) was reported after treatment and was maintained during the next three months. While at baseline 74% of the patients were still taking analgesics, three months after treatment the rate dropped to 48% [87]. The attention patients received due to the hyperthermia intervention must be considered with respect to subjective outcomes such as pain. Considering adverse events, it has to be noted that pain was also caused by the EH treatment in one study [81] and 20% of the participants refused further EH sessions because of pain [83]. Overall, heterogeneous results on the endpoint pain were documented.

QoL was addressed in six studies including 117 patients. In one study with WBH changes in patient’s well-being were again documented for those patients whose disease responded to therapy [71]. Another study with 22 patients using WBH reported an improvement in QoL [61]. No significant differences in the QoL were seen from baseline to three months after EH with RTx in one study with 10 patients [83] and in another study [82] with initially 19 patients. Another study using EH with 23 participants [87] showed that except for nausea and vomiting, loss of appetite, diarrhoea and financial problems, the patients’ quality of life improved significantly in all the functional scales within three months. Also these results may be explained in part by the attention received during the application of hyperthermia.

One study with 37 patients treated with WBH and CTx reported the outcome fatigue [71]. In total, 34 patients complained about grade one to two fatigue and one patient suffered from grade three fatigue prior to treatment. After treatment, all 16 patients with objective tumour responses reported, as is to be expected, an improved sense of well-being. On the other hand, fatigue is also ascribable to WBH, as reported in five studies [68, 69, 73, 74, 88], or to ECC-WBH, as documented in two studies [78, 89].

#### Endpoint survival

Survival data were documented in 15 single-arm studies and in one case series. Median overall survival was analysed in 14 studies, but explicit data for OS being calculated from first diagnoses can be found in 3 studies [60, 61, 81]. OS from first diagnoses ranged from 18.6 months [81] to 19.3 months [60, 61]. The TTP (time to progression) or the PFS (progression-free survival) measured in ten studies ranged from 2.5 months [82] to 6.8 months [60, 61]. The 1-year OS was part of analysis in five studies and ranged from 30% [86] to 68% [60, 61]. Due to lead time bias, the data have to be treated with caution. By reason of the design of single-arm studies, no data are available about the lifetime of a control group, so it is not possible to derive a clear statement in which way hyperthermia may influence survival data.

### Adverse events

Detailed information to the different side effects can be found in Tables 3 and 6.

#### Related to WBH

In the SR by Lassche et al. [45], myelosuppression grade 3 and 4 occurred most frequently in studies using WBH along with CTx. Grade 3 and 4 side effects that were directly attributable to WBH therapy included cardiac arrhythmias, dermal side effects and kidney failure. Four patients died of treatment-related complications [48, 60, 66]. In the prospective cohort study by Gerke et al. [49] 43 patients with advanced sarcoma were divided into three groups (ICE (ifosfamide, carboplatin and etoposide)-CTx in combination with extracorporeal WBH (e-WBH), ICE-CTx with r-WBH by infrared radiation (Aquatherm) or only ICE-CTx). In both hyperthermia groups, the time at a target temperature of 41.8 °C was one hour. On the third day of the cycle, the glomerular filtration rate (GFR) decreased significantly more in the WBH groups than in the group treated with ICE-CTx alone and no difference between e-WBH and r-WBH was found (*p* = 0.364). Three weeks after the start of the CTx-cycle, GFR and the serum creatinine showed no significant difference between the different treatment modalities. In summary, nephrotoxicity sees to be aggravated by WBH immediately after chemotherapy especially when nephrotoxic agents are used along with WBH.

In the RCT by Robins et al. [47] 16 patients with different types of advanced cancer were treated with WBH (Aquatherm) alone during week 1. Thereafter, they were randomized to receive either Melphalan alone in the second week, Melphalan plus WBH for one hour at a target temperature of 41.8 °C in the fifth week or the reverse sequence. Across all CTx levels for Melphalan + WBH the average mean nadir WBC count was 35% and the mean nadir platelet count was 20% lower compared to Melphalan alone (*p* = 0.006, *p* = 0.04), denoting that myelosuppression was more pronounced in cycles with WBH.

Lesions of the skin were a frequent side effect. In one study with 9 participants treated with WBH, 60% showed a transient erythema and in one subject two round thermal lesions (grade 2) appeared [46], respectively; 3 of 44 patients showed pressure scores [48]. In the lower class of evidence skin lesions, most pronounced in grade 1 were also a frequent side effect [59, 60, 62, 64, 66, 71–74, 88]. The lesions included blisters, erythema, burns and ulcers especially at contact points with the heating pads. Besides waterbed and infrared rays, WBH also uses insulating measures and thermal blankets. These can then cause the skin lesions.

According to the authors, herpes simplex infections were attributed to WBH. In one study 39% were detected with mucosal herpes infections, responsive to acyclovir [48] or such infections (grade 1) occurred in 7 of 16 patients [47]. In the lower class of evidence herpes infections were also often reported [65, 68–70, 73, 74] and the incidence ranged from 17% [70] to 73% [74].

In the RCT by Robins et al. [47] a transient increase in liver function tests (grade 2) was seen in 3 patients and additionally low-grade fever (< 24 h post-treatment) occurred to 3 patients. While taking a closer look at serum chemistries in the lower class of evidence, liver enzymes showed a significant elevation 24 h post-WBH [74], but returned to normal range at follow-up [74, 88]. Another study reported a transient elevation in liver enzymes in 25% [67].

A fatigue syndrome grade 3 and 4 was noted in a quarter of the patients in cycles with WBH; compared to cycles without WBH, grade 3 and 4 occurred in 9% [48]. In the lower evidence class, fatigue was mentioned likewise [68, 69, 73, 74, 88] and the incidence ranged from 14% [88] to 100% [74]. Transient cardiac arrhythmias with electrocardiographic signs of myocardial ischaemia (WHO grade 3) concerned to 5 of overall 44 patients in the CT by Hegewish-Becker [48]. In single-arm studies, case series and case reports, cardio-circulatory events were reported as arrhythmic episodes [59, 60, 68, 74, 88], depression of blood pressure [68, 70, 73, 74, 88] or an increase in heart rate [67, 73, 74, 88].

In addition, in studies of the lower class of evidence, following adverse events were mentioned, which were related to WBH, according to the authors. After treatment, some patients suffered from slight headache (between grade one and two) [65, 68–70, 72]. Neurological adverse events were peripheral neuropathy of the femoral, peroneal and ulnar nerve [73, 88], psycho-motoric dysfunctions (grade one and two) [59], convulsions, hallucination (grade one and two) and severe rhabdomyolysis [73]. Further side effects, related to WBH, included post-hyperthermia fevers, lasting for a maximum of 36 h after treatment [65, 68, 73, 88], urinary tract infections [65, 68, 69], nausea and vomiting at most grade two [67, 68, 73], diarrhoeas between grade one and two [65, 70, 73, 88] renal failure grade one or two [59] and calf thrombophlebitis [68].

#### Related to therapeutic fever

In one study by Reuter et al., patients experienced nausea and vomiting, headache, back pain, circulatory reactions and weakness the following days [50].

#### Related to ECC-WBH

Side effects ascribable to that modality were reported in studies of the lower class of evidence. Probably ascribable to ECC-WBH were 4 deaths (24%) due to intra-abdominal bleeding, 1 death due to lung oedema and to 2 due to hepato-renal syndrome [75]. Proteinuria grade 1 to grade 3 was reported in three patients [89]. Transient liver failure occurred in 1 patient [89, 91] and hepatitis was detected in 3 participants [77]. During ECC-WBH alone elevation of bilirubin, albumin, lipase, AST (aspartate aminotransferase), ALT (alanine transaminase), γGT (gamma-glutamyl transferase) could be found [89] or AST, ALT and bilirubin significantly increased 24 h after treatment in another study [91]. Moreover, perioral herpes infections occurred [76, 77, 89]. Skin lesions included burns [89], pressure scores [76, 77] or a skin imprint [78]. Several studies reported side effects which were most probably due to cardiovascular stress such as mild anasarca [76, 77], episodes of hypotension grade two [89, 91] or the significant increase of the heart rate during heating [76, 77, 89, 91]. Moreover, the administration of catecholamines or crystalloid solutions were necessary [76, 77, 89, 91]. Adaptation to warming during ECC treatment may well be associated with circulatory difficulties and is therefore not suitable for a wide patient population. Further side effects included an infection of the shunt necessitated the removal of graft in 1 patient [75], changes in coagulation parameters [77, 89, 91]. Fatigue [89], nausea [77, 89], vomiting [77] and diarrhoea [76, 77] were documented with grade 1 or 2. Moreover, between slight to severe weakness of muscles [75], reversible paresthesia grade 2 [77] and post-treatment fever grade 1 [89] occurred. In the combination ECC-WBH with CTx 11% [76], 8%, respectively, [77] of the participants developed acute renal failure, requiring haemodialysis and nephrotoxicity was reported in 42% of the participants [77]. Neurological problems included encephalopathy in one patient, who died 3 weeks later, but according to the authors, the death was not attributed to treatment [78].

#### Related to EH

In the RCT by Minnaar et al. [51], adipose tissue burns occurred in 9.5% and pain in 9% of patients treated with EH. In the cohort study by Kim et al. [57] in the first 60 days after treatment, a significantly higher opioid dose was used in the group treated with hyperthermia (*p* = 0.022). Comparing the arms in the RCT by Loboda et al. [54] which included treatment with and without EH, there were no differences in haematological and gastrointestinal toxicities or liver and kidney function. In the RCT by Mahdavi et al. [55] EH-related side effects were mild headache with no necessity for any additional medication. In the RCT by Kim et al. [58] fat necrosis and hot spots occurred in 1 patient each. Comparing arm A, which received EH in addition to CTx and RTx, with arm B, the number of side effects did not differ significantly, except for gastrointestinal side effects. These occurred significantly more frequently in arm B (*p* = 0.01). Adverse events caused by EH in the RCT by Fiorentini et al. [56] included headache, scalp burn and seizures. More than an hour after treatment, seizures occurred in 4 additional patients.

In the lower class of evidence, local pain grade 1 occurred to 20%, grade 2 was documented in 60% [81], and in the study by Yu et al., 20% of the participants refused further EH sessions because of pain [83]. Typical symptoms of increased intracranial pressure, e.g. nausea (grade one to two), confusion (grade one to three), somnolence (grade two to three) and focal neurological symptoms, for example aphasia (grade three to four) or hemiparesis (grade two to three), were documented in 1 study [81]. Furthermore, 87% of the patients suffered from headache up to grade 4 in 1 study [81].

### Adverse events in studies with multiple interventions

A brief characterization of these studies can be found in Table 7, and information about the adverse events related to the hyperthermia treatment in Table 8.

#### Related to WBH

The side effects are in accordance with the prior reported adverse events. The incidence of the skin lesions was up to 100% [99, 137] and the severity was up to grade 3 [99, 135, 137]. Herpes infections were a common side effect [99, 126, 127, 137], and the incidence was also up to 100% [99]. Neurological side effects included encephalopathy in overall 3 patients [112, 137], which reversed spontaneously. Moreover slight CNS (central nerve system) dysfunctions or disorientations [112, 137], neuropathy or transitional psychosis [137] was reported. During treatment tachycardia [137] or tachyarrhythmia [112] occurred. Fatigue as an adverse event was noticed in two studies, and the incidence ranged from 24% [126] to 100% [99]. Further side effects included headache [126], haemodynamic depression [137], nausea and vomiting [126, 127], transient elevation in liver function tests [127] and pain [137].

#### Related to EH

Skin lesions consisted of local redness, subcutaneous fibrosis of fatty tissue and slight skin burns [102, 124, 125, 130, 141]. A small number of participants suffered from short-term asthenia, headache, abdominal or local pain and chest pressure [124, 125, 130, 141]. In the study by Yu et al. 30% of the patients refused further EH sessions, mainly because of pain. To complete treatment, the administration of opioids was necessary [139]. Further adverse events related to EH included vomiting or tachycardia [124].

## Discussion

We categorized the studies into two levels of evidence. The higher evidence level one includes SR, RCTs, CTs and cohort studies, whereas evidence level two reports on single-arm studies, case series and case reports.

### Studies evidence level one with methodologically acceptable evaluation

#### Potential benefits of WBH

The SR by Lassche et al. [45] showed no benefit of the invasive WBH treatment. Due to the absence of two-arm studies in the SR, the effect of WBH as addendum on tumour response rate is only speculative. With the numerous grade 3 to 4 side effects, attributable to WBH, the risk/benefit ratio clearly shifts the side of the risks.

#### Potential benefits of EH

In the RCT by Loboda et al. [54] the 10-year OS in the arm with EH was significantly higher, although no significant difference was seen in tumour response. It must be critically noted with regard to the 10-year OS that no exact numbers are given, but only illustrations, from which no exact data can be read off. In addition, no information about the method of randomization is given. In the RCT by Minnaar et al. [52] the intervention group showed significant improvements in some sub-items (social functioning, emotional functioning, fatigue and pain) of the EORTC questionnaire compared to control. Furthermore, the group treated additionally with EH achieved significantly better values at six-month local disease-free survival, local disease control and tumour response. The reasons for the missing data of part of the participants are not stated; therefore, selective reporting cannot be excluded. Additionally, with such a high drop-out rate and without any reasons given, the comparability of the groups cannot further be assumed. It is therefore possible that healthier or more motivated patients remained in the study. Those patients then may achieve a better result and do not constitute a representative sample [51]. Although, six months after treatment, the tumour volume in the intervention group was lower than in the control group in the RCT by Mahdavi et al. [55], neither OS after 18 months nor the Karnofsky Performance Status Scale showed any benefit. However, due to missing comparison of the patient and tumour characteristics at baseline, it is unclear whether the groups already differed from the beginning and the intervention group may have had better values all along. Therefore, no valid interpretation of these results is possible. In addition, a rationale for the allocation to the two treatment arms is not specified [55].

### Studies evidence level one with methodologically low evaluation

#### Potential benefit of WBH

WBH did not improve the quality of recovery, as no significant difference was found in the RCT by Sulyok et al. [46] for the quality of recovery after surgery overall or in the dimensions assessed (global QoR-40, *p* = 0.81). On the other hand, despite the fact that in vitro data demonstrated that hyperthermia distinctively enhances the cytotoxic side effects of oxaliplatin [142], and the incidence of toxicities most likely related to chemotherapy and was hardly different between the chemotherapy cycles treated with or without WBH in the phase-II-CT by Hegewish-Becker et al. [48].

#### Potential benefit of EH

In the retrospective cohort study by Kim et al. [57] the EAS over the time showed worse values in the EH group and a significant higher opioid dose within the first 60 days. In another retrospective cohort study by Kim et al. [58] treating the intervention group with EH, no significant difference was found in the pathologic outcome. Moreover, no difference was seen in 2-year OS. The only significant difference was reported in the EH group in the two-year locoregional recurrence-free survival. In the retrospective cohort study by Fiorentini et al. [56] the intervention group was treated only with EH, whereas the control arm, received best supportive care together with CTx. The overall response rate and the median OS were significantly better in the hyperthermia group, although the difference in survival was only a few months. With regard to the methodology of the study, the results must be considered with caution. No demographic parameters comparing the intervention and control group at baseline as well as information on prior treatments are not specified. Even if a benefit of hyperthermia treatment is first conveyed in these retrospective studies, this cannot be considered an evidence-based benefit due to the methodological limitations of the studies.

### Adverse events in evidence level one and well-reported adverse events in evidence level two

#### Side effects related to WBH/ECC-WBH

The SR of Lassche et al. [45] points to several grade 3 and 4 toxicities, the invasiveness of the procedure, the elaborate supportive care and the high costs. Moreover, the RCT by Robins et al. [47] showed that myelosuppression was more pronounced in cycles with WBH. One major problem of hyperthermia is organ toxicity as nephrotoxicity. Special care should be taken with patients with restricted kidney function [49]. Other reported adverse events included cardio-circulatory stress during WBH or ECC-WBH. So patients with higher grade of cardiac arrhythmias have to be excluded [3] as arrhythmic episodes occurred regularly [59, 60, 62, 68, 74, 88, 137] and heart rate increased [67, 73, 74, 76, 77, 88, 89, 91] during heating. Therefore, cardiologic examinations must be carried out to prove the patients’ cardio-pulmonary capacity before treatment and continuous cardiac monitoring during WBH is necessary [62]. To maintain a sufficient blood pressure, crystalloid solutions and/or catecholamines were needed during plateau phase [76, 77, 89, 91]. Furthermore, patients with markedly restricted hepatic capacity have to be precluded [3], because during WBH, hepatic dysfunction was mentioned [60, 66, 70, 127] or a transient elevation in liver enzymes occurred [67]. Another severe side effect is the more pronounced myelosuppression in cycles with WBH [47]. In addition, the application of WBH for patients with cerebral or spinal metastases should be critically reconsidered, because there may be the risk of an increase in intracranial or intraspinal pressure [3, 97]. In particular, the use of ECC-WBH should be reconsidered critically, as due to the high invasiveness 4 deaths from overall 76 patients treated were attributed to ECC-WBH [75]. Additionally, elevation of liver enzymes occurred [89, 91] and participants developed acute renal failure, requiring haemodialysis [76, 77]. Moreover, most of the patients needed analgo-sedation or deep anaesthesia during WBH or ECC-WBH [3].

#### Side effects related to EH

A somewhat milder side effect profile was seen with EH treatment. EH-related side effects were mild headache with no necessity for any additional medication [55]. Other adverse events included fat necrosis or hot spots [51, 58], scalp burn, seizures [56] and pain [51]. In the cohort study by Kim et al. [52] a significantly higher opioid analgesic dose was used in the group treated with hyperthermia. (*p* = 0.022) or participants refused further EH sessions because of pain [83].

### Risk/benefit ratio

When considering the risk/benefit ratio, it becomes apparent that, due to the very heterogeneous results and methodological limitations of the included studies, clinical evidence for the benefit of alternative hyperthermia in cancer patients is still lacking. Based on the current research, the adverse events outweigh the potential but yet unproven benefits of alternative hyperthermia.

### Absence of intra-tumoural temperature measurement in alternative hyperthermia

Moreover, the term hyperthermia is misleading because it is not clearly defined. Based on the rationale behind hyperthermia, some clinical studies with conventional hyperthermia were able to show evidence-based benefits for selected types of cancer [143]. These hyperthermia treatments were carried out with defined quality standards, including an intra-tumoural temperature measurement every minute, a exactly determination of the treatment area previous via MR (magnetic resonance) or CT (computer tomography) or the presence of an engineer or physicist during treatment [144]. Invasive temperature probes represent the gold standard in thermometry. For non-invasive monitoring, CT-, MR- and ultrasound-based thermometry methods have been developed although these do not deliver the same accuracy [145, 146]. Exact temperature control is essential in hyperthermia, on the one hand to avoid side effects [147], on the other hand to reach the desired target range, with regard to the close dose–effect relationship [145, 148]. Provider of alternative hyperthermia concepts also advertise with the same mentioned theoretical principles, but the implementation of uniform rules is missing. In the present review, information about temperature measurements is lacking in 15 studies [46, 50, 55, 57, 58, 64, 81, 82, 84, 85, 87, 94–97]. In the studies by Minnaar et al. [51–53], the authors do not consider it necessary to measure the temperature. In only one study with electro hyperthermia thermal mapping was performed in accordance with the guidelines of the European Society of Hyperthermic Oncology, according to the authors, but further information about the exact way of measuring temperature is missing [25–30, 80]. In one study WBH temperature was measured constantly by a probe placed in the centre of the tumour [93]. In another study with EH intra-tumoural temperature was approximately determined using a thermal imaging camera [54]. The other studies did not measure the intra-tumoural temperature directly but only offered indirect methods for which clinical data are missing [47–49, 59–63, 65–78, 83, 86, 88–92].

### Limitations of this work

Some limitations of this systematic review must be mentioned. First, we focused on adults, omitting literature that included more than 20% children as patients. This is however not a limitation as paediatric patients generally form an even more heterogeneous population than adult patients, making solid conclusions about clinical effects of alternative hyperthermia even more challenging. So excluding these categories is not expected to change our conclusions. Also, studies that were not in English or German were also not considered. However, including these would have made our in-depth analysis of content and methodology too challenging.

## Conclusion

No clear statement regarding the efficacy of hyperthermia treatment in complementary medicine on cancer patients may be derived from published studies. Further randomized controlled trials are necessary, which compare groups treated with standard cancer therapy to groups treated with alternative hyperthermia along with standard cancer therapy to draw conclusions whether alternative hyperthermia influences tumour response, survival data or the quality of life and to find out, which side effects are exactly assignable to hyperthermia. Due to this and to the heterogeneous results of the systematic review regarding the outcomes pain and quality of life, no benefit of alternative hyperthermia has been shown and no evidence-based indications can be stated. The adverse events especially of WBH and ECC-WBH may overweigh the potential benefit. Physicians should not prescribe WBH or ECC-WBH in case of comorbidities like renal or hepatic diseases, cardiac arrhythmias, cardia-pulmonal insufficiency, increased intracranial or intraspinal pressure or existing aspects impeding the essential analogue sedation or anaesthesia.

Due to the highly different methods offered with the same terminus hyperthermia, it is especially difficult for patients to distinguish between scientifically proven hyperthermia treatments and alternative hyperthermia methods.

To help patients and physicians who are not experts in oncology, institutions which offer or evaluate conventional hyperthermia treatments should clearly differentiate their procedures from the offers of alternative providers in words also comprehensible for patients.

## Supplementary Information

Below is the link to the electronic supplementary material.

Supplementary file1 (DOCX 92 kb)

Supplementary file2 (DOCX 82 kb)

Supplementary file3 (DOCX 235 kb)
