# Supplementary material for: Systematic review about complementary medical hyperthermia in oncology
Source: Clin Exp Med. 2022 Jun 29;22(4):519–65. doi: 10.1007/s10238-022-00846-9 (PMC9244386; doi:10.1007/s10238-022-00846-9)
Supplement: Supplementary file 1 — Supplementary file1 (DOCX 92 kb) [file 10238_2022_846_MOESM1_ESM.docx]

## Supplementary table 1: Risk of bias of studies of the first level of evidence (SR, RCTs, CTs and cohort studies)

| **Reference** | **Study type** | **Standardized rating of risk of bias** | **Additional comments on methodology** | **Evidence Level (Oxford)** |
| --- | --- | --- | --- | --- |
| **Lassche (2019) [1]**  Included studies in the SR:   - Atmaca et al.   (2009) [2]   - Douwes et al.   (2004) [3]   - Westermann et   al. (2001) [4]   - Richel et al.   (2004) [5]   - Hegewisch-   Becker et al.  (2002) [6]   - Bakhshandeh-   Bath et al.  (2009) [7]   - Westermann et   al. (2003) [8]   - Wiedemann et   al. (1996) [9]   - Bakhshandeh   -Bath et al. (2003) [10]  Hildebrandt et al. (2004) [11]  Engelhardt et al. (1982) [12]  Neumann et al. (1982) [13]  Bull et al. (1992) [14]  Engelhardt et al. (1990) [15] | **SR**  SA  SA  SA  SA  CT  SA  SA  SA  SA  Un-  clear  SA  SA  SA  SA | AMSTAR:  Positive: 4 points  Partial positive: 2 points  Negative: 7 points  See below, study included in our review  See below, study included in our review  See below, study included in our review  See below, study included in our review  See below, study included in our review  See below, study included in our review  See below, study included in our review  See below, study included in our review  See below, study included in our review  IHE:  Positive: 6 points  Negative: 6 points  Unclear: 6 points  Partial: 2 points  No full-text available  IHE:  Positive: 2 points  Negative: 7 points  Unclear: 6 points  Partial: 5 points  IHE:  Positive: 6 points  Negative: 6 points  Unclear: 5 points  Partial: 3 points  IHE:  Positive: 5 points  Negative: 5 points  Unclear: 5 points  Partial: 5 points | PRO: Detailed search string. Study report available. Detailed description of each treatment and additional treatments.  CONTRA: Moderate quality of all included studies. Small patient samples in the studies (n <100). Includes only non-randomized studies.  CONTRA: Multiple intervention, systemic cancer multistep therapy. Inadequate study setting.  CONTRA: Unclear, if patients were recruited consecutively. Incomplete description of included patients. Side effects without grading. No information about funding or COI. No information about ethics approval.  CONTRA: No information about funding or about the COI. Unclear, if patients were recruited consecutively. No information about the period of time.  CONTRA: No further description of characteristics of patients. No information about funding or about the COI. Unclear, if patients were recruited consecutively. Side effects without grading. | 2a  4  4  4  4  2b  4  4  4  4  4  4  4  4 |
| Sulyok et al. (2012) [16] | RCT | SIGN-2 (controlled trials):  Positive: 5 points  Can’t say: 2 points  Negative: 1 point  Does not apply: 1 point  Overall quality: low | PRO: No drop-out/attrition. Randomized with a computer-generated randomization list. Power-analysis even if the number of participants is very low.  CONTRA: Small number of participants, n per group: n=9. Comparability of the groups and basics conditions not assessable, because no information about histology, type of cancer, severity, suffering from other diseases and possible additionally other therapies in none of the groups given. No comparison of the demographic variables. Statistical methods: quality of recovery: no information about average, adjusted averaging difference, 95% CI, effect size, global score: no p-value. No information about the number of participants, editing questionnaire. Side effects collected without systematically or standardized methods. | 2b |
| Robins et al. (1997) [17] | RCT | SIGN-2 (controlled trials):  Positive: 5 points  Negative: 2 points  Can’t say: 1 point  Does not apply: 1 point  Overall quality: acceptable | PRO: Description of previous therapy and tumour site. Cross-over-design: every participant reached WBH alone, thereafter: Melphalan alone than Melphalan + WBH or reverse sequence.  CONTRA: No further information about COI and ethics approval. Spot check very heterogeneous, perhaps also selective (different entities of cancer). No description of possible additional co-interventions. Small number of participants (n=16). Due to study design, carry-over effects can’t be ruled out. | 2b |
| Hegewisch-Becker et al. (2002) [6] | CT | SIGN-2 (controlled trials):  Positive: 4 points  Not relevant/does not apply: 5 points.  Overall quality: low | PRO: Detailed description of pre-treatments, number and location of metastases. Validation of the outcome: measurable lesion, proved by imaging. Power-analysis existing. Number of drop-outs and adverse events given. No need for randomization, because all patients received in a weekly change CTx with or without WBH. Comparability of the two groups given.  CONTRA: Small number of participants, n=44. Due to weekly change, carry-over effects can’t be ruled out. Mixture of a potential effect of the WBH-treatment possible. Declaration of the authors, that neurosensory side effects less pronounced in cycles combined with WBH, not supported by the results. Statistically methods: comparison of the side effects of the two different treatment options without statistical substantiation (no information about p-value, 95%-CI and effect size), even though results obviously balanced. | 2b |
| Gerke et al. (2000) [18] | Cohort, prospective | SIGN-3 (cohort studies):  Positive: 6 points  Negative: 7 points  Does not apply: 1 point  Overall quality: low | PRO: At T3 (day 3 of ICE): information about drop out/adverse events. Validation of the outcome.  CONTRA: Small number of participants, n=43. No blinding. Basic conditions not assessable, because no information about histology, severity, suffering under other diseases, possible additionally other and prior therapies and demographic variables. Comparison of the groups not given. No reasons for allocation to the different treatment options. Statistical methods: no information about average, adjusted averaging difference, standard error 95% CI. Side effects collected without systematically or standardized methods. | 2b- |
| Reuter et al. (2018) [19] | CT, retrospective | SIGN-2 (controlled trials):  Positive: 0 points  Negative: 4 points  Can’t say: 1 point  Does not apply: 4 points  Overall quality: unacceptable | CONTRA: No information about basic demographic values. Allocation to the three different treatment-groups unclear. Different number of participants in treatment groups. No information about number of included participants, about validation of the outcome, about way of temperature measurement and about severity of side effects and classification of the adverse events. Spot check very heterogeneous, perhaps also selective. Many different entities of cancer and other illnesses, no information about severity. Dubious presentation of materials and methods. No description of possible additional co-interventions. Unclear, if patients were recruited consecutively. Inclusion and exclusion criteria not clear for participants. Unclear why study was conducted retrospective. “Not need for ethics approval and consent.”-doubtful. Long interval between treatment and publication. (Group A1 start: 2006, published: 2018). | 4 |
| Loboda et al. (2020) [20] | RCT | SIGN-2 (controlled trials):  Positive: 4 points  Can’t say: 3 points  Negative: 1 point  Does not apply: 1 point  Overall quality: acceptable | PRO: No drop-out/attrition. At beginning no significant difference between arms regarding age, tumour stage, histological type and tumour subtype. N=200 participants. Outcomes measured with valid methods.  CONTRA: No information about method of randomization. No information about possible additionally other therapies. Statistics: comparison blood flow and blood pressure: no p-value for comparison before and after EH-treatment, no comparison of blood flow and blood pressure between the two arms. In abstract: intervention achieved a significantly higher rate of objective responses, but individual values for a complete response, partial response or stable disease: no significant difference. 10y-OS: no numbers of lifetime extension, vague calculation. | 2b |
| Minnaar et al. (2019, 2020, 2020) [21-23] | RCT | SIGN-2 (controlled trials):  Positive: 7 points  Can’t say: 0 points  Negative: 1 point  Does not apply: 1 point  Overall quality: acceptable | PRO: Information about method of randomization. At beginning no significant difference between arms regarding HIV Status, FIGO staging, ECOG staging, age and BMI. Number of adverse events given. Outcomes measured with valid methods.  CONTRA: No information about prior treatment. No description of possible additional co-interventions. For outcomes local disease control and tumour response no reasons for drop out of part of the participants. Comparison of the arms regarding therapy not possible. | 2b |
| Mahdavi et al. (2020) [24] | CT, prospective | SIGN-2 (controlled trials):  Positive: 2 points  Not relevant/does not apply: 7 points.  Overall quality: acceptable | PRO: No drop-out/attrition. At beginning no significant difference between arms regarding tumour volume.  CONTRA: Small number of participants (n=38). No information about allocation to different arms. No information about possible additionally other therapies. Inadequate description of the demographic values. Statistics: no p-value for difference in patient characteristics before treatment. Only short report of adverse events. No information about temperature measurement. | 4 |
| Fiorentini et al. (2019) [25] | Cohort, retrospective | SIGN-3 (cohort studies):  Positive: 5 points  Negative: 4 points  Can’t say: 3 points  Does not apply: 2 points  Overall quality: low | PRO: Multicenter study, outcome measured with valid methods.  CONTRA: Different CTx-regimes in arm B, no CTx in arm A. Functional recovery only measured by ECOG-grading. Demographic characteristics before treatment: no distinction between arm A and B. Unclear, if patients were recruited consecutively. Selection basis for the hyperthermia group: patient’s intention to treat. No information about prior treatments. | 2b- |
| Kim et al. (2015) [26] | Cohort, retrospective | SIGN-3 (cohort studies):  Positive: 8 points  Negative: 1 point  Can’t say: 3 points  Does not apply: 2 points  Overall quality: low | PRO: PSM between the hyperthermia and control group. PS calculated for each patient by logistic regression analysis. Covariates: age, gender, year of initial diagnosis, tumour site, cytologic type, TNM stage. Due to PSM: comparability of the two groups in term of known variables given. Validation of the outcome (standard scale of pain, measurement of opioid analgesic dose). Detailly description of: demographic values, year of initial diagnosis, cancer site, cytologic type, TNM-stage. Due to linear mixed effects model for measure EAS changes over time: no imputation techniques for missing data necessary. Adequate use of statistic methods. Inclusion of variables as covariates (even those, over which were not matched at all).  CONTRA: Small number of participants, in the end n (total)=65. No blinding. No description of conventional cancer therapy, no information, if cancer therapies of the two groups are comparable. No information about possible further therapies outside the study. Side effects not reported. Reason for drop out only for n=6, no information about further drop-outs and adverse events. At BL 47.4% of the initially matched control-group (arm B) 🡪 due to PSM: groups in term of important variables actually comparable, but owing to high drop-out the comparability is limited and perhaps not given anymore. | 2b- |
| Kim et al. (2021) [27] | Cohort, retrospective | SIGN-3 (cohort studies):  Positive: 5 points  Negative: 5 points  Can’t say: 3 points  Does not apply: 1 point  Overall quality: low | PRO: Description of prior treatment. Information about patient characteristics before treatment, therefore no significant differences between arms. Number of drop outs given.  CONTRA: Dose of RTx different between groups, another grading system for EH-related adverse events. No information about reasons for lost of follow up. No reasons for allocation to the different treatment options. Unclear, if patients were recruited consecutively. Statistical methods for pathological outcome: no information about average, adjusted averaging difference, standard error, 95% CI. | 2b- |
| AMSTAR: a measurement tool to assess systematic reviews. BL: baseline. BMI: body-mass-index. CI: confidence interval. COI: conflicts of interests. CT: controlled trial. CTx: chemotherapy. EAS: effective analgesic score. EH: electro hyperthermia. ICE: CTx of Ifosfamide, Carboplatin and Etoposide. OS: overall survival. PS: propensity score. PSM: propensity score matching. RCT: randomized controlled trial. SA: single-arm-study. SIGN: Scottish Intercollegiate Guidelines Network Methodology. SR: systematic review. TNM: classification of malignant tumours (tumour, lymph nodes, metastasis). WBH: whole-body hyperthermia. | | | | |

1. Lassche G, Van Herpen CML, Crezee J. Whole-body hyperthermia in combination with systemic therapy in advanced solid malignancies. Critical Reviews in Oncology/Hematology. 2019; 139: 67-74. http://dx.doi.org/10.1016/j.critrevonc.2019.04.023.

2. Atmaca A, Al-Batran S-E, Neumann A, et al. Whole-body hyperthermia (WBH) in combination with carboplatin in patients with recurrent ovarian cancer - a phase II study. Gynecologic oncology. 2009; 112(2): 384-8. https://dx.doi.org/10.1016/j.ygyno.2008.11.001.

3. Douwes F, BogoviC J, Douwes O, Migeod F, Grote C. Whole-body hyperthermia in combination with platinum-containing drugs in patients with recurrent ovarian cancer. International journal of clinical oncology. 2004; 9(2): 85-91.

4. Westermann AM, Grosen EA, Katschinski DM, et al. A pilot study of whole body hyperthermia and carboplatin in platinum-resistant ovarian cancer. European journal of cancer (Oxford, England : 1990). 2001; 37(9): 1111-7.

5. Richel O, Zum Vorde Sive Vording PJ, Rietbroek R, et al. Phase II study of carboplatin and whole body hyperthermia (WBH) in recurrent and metastatic cervical cancer. Gynecologic oncology. 2004; 95(3): 680-5.

6. Hegewisch-Becker S, Gruber Y, Corovic A, et al. Whole-body hyperthermia (41.8 degrees C) combined with bimonthly oxaliplatin, high-dose leucovorin and 5-fluorouracil 48-hour continuous infusion in pretreated metastatic colorectal cancer: a phase II study. Annals of oncology : official journal of the European Society for Medical Oncology. 2002; 13(8): 1197-204.

7. Bakshandeh-Bath A, Stoltz AS, Homann N, Wagner T, Stolting S, Peters SO. Preclinical and clinical aspects of carboplatin and gemcitabine combined with whole-body hyperthermia for pancreatic adenocarcinoma. Anticancer research. 2009; 29(8): 3069-77.

8. Westermann AM, Wiedemann GJ, Jager E, et al. A Systemic Hyperthermia Oncologic Working Group trial. Ifosfamide, carboplatin, and etoposide combined with 41.8 degrees C whole-body hyperthermia for metastatic soft tissue sarcoma. Oncology. 2003; 64(4): 312-21.

9. Wiedemann GJ, Robins HI, Gutsche S, et al. Ifosfamide, carboplatin and etoposide (ICE) combined with 41.8 degrees C whole body hyperthermia in patients with refractory sarcoma. European journal of cancer (Oxford, England : 1990). 1996; 32A(5): 888-92.

10. Bakhshandeh A, Bruns I, Traynor A, et al. Ifosfamide, carboplatin and etoposide combined with 41.8 degrees C whole body hyperthermia for malignant pleural mesothelioma. Lung cancer (Amsterdam, Netherlands). 2003; 39(3): 339-45.

11. Hildebrandt B, Drager J, Kerner T, et al. Whole-body hyperthermia in the scope of von Ardenne's systemic cancer multistep therapy (sCMT) combined with chemotherapy in patients with metastatic colorectal cancer: a phase I/II study. International journal of hyperthermia : the official journal of European Society for Hyperthermic Oncology, North American Hyperthermia Group. 2004; 20(3): 317-33.

12. Engelhardt R, Neumann H, von der Tann M, Löhr GW. Preliminary results in the treatment of oat cell carcinoma of the lung by combined application of chemotherapy (CT) and whole-body hyperthermia. Progress in clinical and biological research. 1982; 107: 761-5.

13. Neumann H, Fabricius H-A, Engelhardt R. Moderate whole-body hyperthermia in combination with chemotherapy in the treatment of small cell carcinoma of the lung: a pilot study. Natl Cancer Inst Monogr. 1982; 61: 427-429.

14. Bull JMC, Cronau LH, Newman BM, et al. Chemotherapy resistant sarcoma treated with whole body hyperthermia (WBH) combined with 1-3-Bis(2-chloroethyl)-1-nitrosourea (BCNU). International Journal of Hyperthermia. 1992; 8(3): 297-304. https://dx.doi.org/10.3109/02656739209021784.

15. Engelhardt R, Müller U, Weth-Simon R, Neumann HA, Lühr GW. Treatment of disseminated malignant melanoma with cisplatin in combination with whole-body hyperthermia and doxorubicin. International Journal of Hyperthermia. 1990; 6(3): 511-5. https://dx.doi.org/10.3109/02656739009140947.

16. Sulyok I, Fleischmann E, Stift A, et al. Effect of preoperative fever-range whole-body hyperthermia on immunological markers in patients undergoing colorectal cancer surgery. British journal of anaesthesia. 2012; 109(5): 754‐61. https://dx.doi.org/10.1093/bja/aes248.

17. Robins HI, Rushing D, Kutz M, et al. Phase I clinical trial of melphalan and 41.8 degrees C whole-body hyperthermia in cancer patients. Journal of clinical oncology : official journal of the American Society of Clinical Oncology. 1997; 15(1): 158-64.

18. Gerke P, Filejski W, Robins HI, Wiedemann GJ, Steinhoff J. Nephrotoxicity of ifosfamide, carboplatin and etoposide (ICE) alone or combined with extracorporeal or radiant-heat-induced whole-body hyperthermia. Journal of cancer research and clinical oncology. 2000; 126(3): 173-7.

19. Reuter URM, Oettmeier R, Hobohm U. Safety of Therapeutic Fever Induction in Cancer Patients Using Approved PAMP Drugs. Translational oncology. 2018; 11(2): 330-7. https://dx.doi.org/10.1016/j.tranon.2018.01.019.

20. Loboda A, Syvak L, Golovko T, et al. Efficacy of Combination Neoadjuvant Chemotherapy and Regional Inductive Moderate Hyperthermia in the Treatment of Patients With Locally Advanced Breast Cancer. Technology in cancer research & treatment. 2020; http://dx.doi.org/10.1177/1533033820963599.

21. Minnaar CA, Kotzen JA, Naidoo T, et al. The effect of modulated electro-hyperthermia on local disease control in HIV-positive and -negative cervical cancer women in South Africa: Early results from a phase III randomised controlled trial. PLoS ONE. 2019; 14(6): e0217894. http://dx.doi.org/10.1371/journal.pone.0217894.

22. Minnaar CA, Kotzen JA, Ayeni OA, Vangu M-D-T, Baeyens A. Potentiation of the Abscopal Effect by Modulated Electro-Hyperthermia in Locally Advanced Cervical Cancer Patients. Frontiers in oncology. 2020; 10: 376. https://dx.doi.org/10.3389/fonc.2020.00376.

23. Minnaar CA, Kotzen JA, Tunmer M, et al. Analysis of the effects of mEHT on the treatment-related toxicity and quality of life of HIV-positive cervical cancer patients. International Journal of Hyperthermia. 2020; 37(1): 263-272. http://dx.doi.org/10.1080/02656736.2020.1737253.

24. Mahdavi SR, Khalafi L, Shivaliloo S, et al. Thermal enhancement effect on chemo-radiation of Glioblastoma multiform. International Journal of Radiation Research. 2020; 18(2): 255-62. http://dx.doi.org/10.18869/acadpub.ijrr.18.2.255.

25. Fiorentini G, Sarti D, Mattioli G, et al. Modulated Electrohyperthermia in Integrative Cancer Treatment for Relapsed Malignant Glioblastoma and Astrocytoma: Retrospective Multicenter Controlled Study. Integrative Cancer Therapies. 2019; 18. http://dx.doi.org/10.1177/1534735418812691.

26. Kim Y-P, Choi Y, Kim S, et al. Conventional cancer treatment alone or with regional hyperthermia for pain relief in lung cancer: A case-control study. Complementary Therapies in Medicine. 2015; 23(3): 381-7. https://dx.doi.org/10.1016/j.ctim.2015.04.004.

27. Kim S, Cha J, You SH, Lee JH. Beneficial effects of modulated electro-hyperthermia during neoadjuvant treatment for locally advanced rectal cancer. International Journal of Hyperthermia. 2021; 38(1): 144-51. http://dx.doi.org/10.1080/02656736.2021.1877837.
