# Supplementary material for: Systematic review about complementary medical hyperthermia in oncology
Source: Clin Exp Med. 2022 Jun 29;22(4):519–65. doi: 10.1007/s10238-022-00846-9 (PMC9244386; doi:10.1007/s10238-022-00846-9)
Supplement: Supplementary file 2 — Supplementary file2 (DOCX 82 kb) [file 10238_2022_846_MOESM2_ESM.docx]

## Supplementary table 2: Risk of bias of studies of the second level of evidence (single arm-studies and case series)

| **Reference** | **Study type** | **Standardized rating of risk of bias** | **Additional comments on methodology** | **Evidence Level (Oxford)** |
| --- | --- | --- | --- | --- |
| Atmaca et al. (2009) [1] | Single-arm | IHE:  Positive: 11 points  Negative: 4 points  Unclear: 3 points  Partial: 2 points | PRO: Number and reasons for drop out described. Validation of the outcome (measurable lesion, proved after every two cycles of therapy by CT, tumour marker and physical examination).  CONTRA: No further information about funding. Unclear, if patients were recruited consecutively. No description of possible additional co-interventions. Loss of information, because age summarized in two categories. | 4 |
| Bakhshandeh-Bath et al. (2003) [2] | Single-arm | IHE:  Positive: 11 points  Negative: 3 points  Unclear: 4 points  Partial: 2 points | PRO: Histology and stage of disease described. Validation of the outcome (measurable lesion, proved by CT).  CONTRA: No further information about the COI. Unclear, if patients were recruited consecutively. No description of possible additional co-interventions. | 4 |
| Bakhshandeh-Bath et al. (2009) [3] | Single-arm | IHE:  Positive: 10 points  Negative: 4 points  Unclear: 4 points  Partial: 2 points | PRO: Histology and site of metastasis described.  CONTRA: Unclear, if patients were recruited consecutively. No description of possible additional co-interventions. No description of the side effects: haematologic, gastrologic and others. Mixture of preclinical and clinical studies. Small number of participants (n=13). No information about the classification of the outcome tumour response. No information about funding or COI. | 4 |
| Bruns et al. (2004) [4] | Single-arm | IHE:  Positive: 7 points  Negative: 4 points  Unclear: 6 points  Partial: 3 points | Clinical results already published: Bakhshandeh (2003), Ifosfamide, Carboplatin and Etoposide combined with 41.8°C whole body hyperthermia for malignant pleural  mesothelioma (Amsterdam, Netherlands).  CONTRA: No further information about the COI. Unclear, if patients were recruited consecutively. Inclusion and exclusion criteria not clear for participants. No description of possible additional co-interventions. No information about timepoint of collecting data for MBS score, no information about QoL-score before treatment. | 4 |
| Katschinski et al. (1997) [5] | Single-arm | IHE:  Positive: 6 points  Negative: 6 points  Unclear: 4 points  Partial: 4 points | PRO: Randomization (early vs late schedule: even vs odd numbered patients entering protocol), cycles alternated between early and late schedules. Two different schedules would be exactly equivalent (40 min CTx at 41.8°C).  CONTRA: No information about the period of time, about basic conditions and about overall observation period. No information about severity of cancer, age, sex and other demographic values. Inclusion and exclusion criteria not clear for participants. Spot check very heterogeneous, perhaps also selective. No information about number of analysed participants and number of drop-outs. Unclear, if patients were recruited consecutively. No description of possible additional co-interventions. No description of side effects. | 4 |
| Richel et al. (2004) [6] | Single-arm | IHE:  Positive: 11 points  Negative: 3 points  Unclear: 4 points  Partial: 2 points | PRO: Patients assessed weekly for side effects. Validated outcome (clinical examination, serum tumour markers and CT). Prior treatment and cytological type of cancer detailly described.  CONTRA: No further information about the COI. No information about ethics approval. Unclear, if patients were recruited consecutively and no information about the period of time. No description of possible additional co-interventions. 18 treatment delays (due to logistical problems, for example, unavailability of anesthesiological personnel: n=11). | 4 |
| Westermann et al. (2001) [7] | Single-arm | IHE:  Positive: 14 points  Negative: 2 points  Unclear: 3 points  Partial: 1 point | PRO: Measurable outcome (physical exams, laboratory evaluation and CT). Prior treatment detailly described.  CONTRA: Unclear, if patients were recruited consecutively. No description of possible additional co-interventions. | 4 |
| Westermann et al. (2003) [8] | Single-arm | IHE:  Positive: 10 points  Negative: 2 points  Unclear: 4 points  Partial: 4 points | PRO: Reasons for drop-out described. Validation of the outcome (measurable lesion, proved by CT).  CONTRA: Unclear, if patients were recruited consecutively. No description of possible additional co-interventions. | 4 |
| Robins et al. (1985) [9] | Single-arm | IHE:  Positive: 9 points  Negative: 6 points  Unclear: 3 points  Partial: 2 points | PRO: Description of previous therapy and tumour site for every patient. Relationship of skin, esophageal and rectal temperature as a function of time.  CONTRA: No information about the COI. No information about period of time, about basic conditions and about overall observation period. Spot check very heterogeneous, perhaps also selective. Measurable disease or objectively evaluable disease preferred but not required. No side effects measurable 🡪 questionable. Dubious presentation of therapy response. Unclear, if patients were recruited consecutively. No description of possible additional co-interventions. | 4 |
| Robins et al. (1988) [10] | Single-arm | IHE:  Positive: 7 points  Negative: 4 points  Unclear: 5 points  Partial: 4 points | PRO: Description of previous therapy and tumour site for every patient. Systematically analysis of haematologic and serum chemistries. All patients received in the same course WBH alone, WBH + CTx and CTx alone.  CONTRA: No information about the period of time, about basic conditions and about overall observation period. Spot check very heterogeneous (different entities of cancer), perhaps also selective. Number of analysed participants and drop-outs dubious. Unclear, if patients were recruited consecutively. No description of possible additional co-interventions. | 4 |
| Robins et al. (1990) [11] | Single-arm | IHE:  Positive: 9 points  Negative: 3 points  Unclear: 5 points  Partial: 3 points | CONTRA: No information about basic conditions and about overall observation period. Confusing presentation: Mixture of clinical trial, in vivo study on mice and in vitro study on lymphoid cell line. No information about prior treatment. Spot check very heterogeneous, perhaps also selective. Study quality regarding comparison of adjunctive therapy (lonidamine or WBH) dubious, so WBH + RTx was analysed as a single-arm study. Unclear, if patients were recruited consecutively. No description of possible additional co-interventions. | 4 |
| Robins et al. (1993) [12] | Single-arm | IHE:  Positive: 8 points  Negative: 5 points  Unclear: 5 points  Partial: 2 points | PRO: All patients received in the same course WBH alone, WBH + CTx and CTx alone.  CONTRA: No information about period of time, about basic conditions and about overall observation period. Spot check very heterogeneous (different entities of cancer), perhaps also selective. Unclear, if patients were recruited consecutively. No description of possible additional co-interventions. No information about the number of analysed participants, drop-outs and the number of patients with PD. | 4 |
| Bull et al. (2008) [13] | Single-arm | IHE:  Positive: 11 points  Negative: 4 points  Unclear: 3 points  Partial: 2 points | PRO: Prior treatment and type of cancer described. Validation of the outcome: measurable lesion, proved by imaging.  CONTRA: Unclear, if patients were recruited consecutively. No description of possible additional co-interventions. Inclusion and exclusion criteria not clear for participants. Spot check very heterogeneous, perhaps also selective (variety of malignancies and different numbers of treatment cycles). QoL not formally assessed. Decrease in pain medication not substantiated with values. | 4 |
| Kraybill et al. (2002) [14] | Single-arm | IHE:  Positive: 9 points  Negative: 4 points  Unclear: 4 points  Partial: 3 points | CONTRA: Unclear, if patients were recruited consecutively. No information about the period of time, about basic conditions and about the overall observation period. No description of possible additional co-interventions and about prior treatments. Spot check very heterogeneous, perhaps also selective. Very small number of participants (n=9). | 4 |
| Steinhart et al. (1996) [15] | Single-arm | IHE:  Positive: 8 points  Negative: 5 points  Unclear: 5 points  Partial: 2 points | PRO: Relationship of esophageal, rectal, bladder and blood temperature as a function of time. Patients randomized into two groups, subjects blinded as to which temperature they received.  CONTRA: No information regarding COI. No information about basic conditions and about the overall observation period. No information about prior treatment. Unclear, if patients were recruited consecutively. No description of possible additional co-interventions. All results not expounded with values and not measured with valid instruments. Very small number of participants (n=6). | 4 |
| Bull et al. (1979) [16] | Single-arm | IHE:  Positive: 6 points  Negative: 7 points  Unclear: 5 points  Partial: 2 points | PRO: Description of previous therapy for every patient.  CONTRA: No information about funding and about the COI. No information about ethics approval. No information about period of time and about basic conditions. Inclusion and exclusion criteria not clear for participants. Spot check very heterogeneous, perhaps also selective. Confusing hyperthermia treatment protocol and unclear information about drop outs and number of analysed patients. Unclear, if patients were recruited consecutively. No description of possible additional co-interventions. | 4 |
| Barlogie et al. (1979) [17] | Single-arm | IHE:  Positive: 6 points  Negative: 6 points  Unclear: 5 points  Partial: 3 points | PRO: ANOVA for statistical changes in laboratory values.  CONTRA: No further information about the COI. Inclusion and exclusion criteria not clear for participants. Spot check very heterogeneous, perhaps also selective. Minor protocol violations in 3 patients. | 4 |
| Gerad et al. (1984) [18] | Single-arm | IHE:  Positive: 9 points  Negative: 5 points  Unclear: 4 points  Partial: 2 points | PRO: Description of previous therapy and tumour site for every patient. One euthermic CTx-course, to obtain BL pharmacokinetic data, then CTx + WBH.  CONTRA: No information about funding or about the COI. No information about ethics approval. No information about the period of time and about basic conditions. Inclusion and exclusion criteria not clear for participants. Spot check very heterogeneous, perhaps also selective. Unclear, if patients were recruited consecutively. No description of possible additional co-interventions. | 4 |
| Locker et al. (2011) [19] | Single-arm | IHE:  Positive: 10 points  Negative: 4 points  Unclear: 4 points  Partial: 2 points | PRO: Validation of the outcome (laboratory parameters, physical examination). Side effects detailly described. Reasons for end of study described.  CONTRA: Prior treatment and severity of cancer not detailly described. No description of possible additional co-interventions. Unclear, if patients were recruited consecutively. No information about the period of time. Inclusion and exclusion criteria not clear for participants. Small number of participants (n=6). | 4 |
| Worel et al. (2014) [20] | Single-arm | IHE:  Positive: 7 points  Negative: 6 points  Unclear: 5 points  Partial: 2 points | CONTRA: Prior treatment and severity of cancer not described. No information about period of time. No description of possible additional co-interventions. Unclear, if patients were recruited consecutively. No information about the period of time. Inclusion and exclusion criteria not clear for participants. Spot check very heterogeneous, perhaps also selective. Small number of participants (n=6). | 4 |
| Koga et al. (1985) [21] | Single-arm | IHE:  Positive: 8 points  Negative: 5 points  Unclear: 5 points  Partial: 2 points | PRO: Description of previous therapy, tumour site and survival time after ECC-WBH for every patient.  CONTRA: No information about funding or about the COI. No information about ethics approval. No information about basic conditions and about the overall observation period. Unclear, if patients were recruited consecutively. No description of possible additional co-interventions. Reduction of pain not measured with valid instruments. | 4 |
| Wiedemann et al. (1994) [22] | Single-arm | IHE:  Positive: 11 points  Negative: 4 points  Unclear: 4 points  Partial: 1 point | PRO: Description of previous therapy and tumour site for every patient. All patients received WBH + CTx and some received CTx alone to obtain BL-pharmacokinetics.  CONTRA: No information about period of time and about overall observation period. Inclusion and exclusion criteria not clear for participants. Unclear, if patients were recruited consecutively. No description of possible additional co-interventions. Result pain before and after WBH treatment not measured with valid instruments. | 4 |
| Wiedemann et al. (1996) [23] | Single-arm | IHE:  Positive: 10 points  Negative: 4 points  Unclear: 4 points  Partial: 2 points | PRO: Diagnosis, tumour site and prior treatment detailly described. Validation of the outcome: measurable lesion, proved by CT.  CONTRA: Unclear, if patients were recruited consecutively. Spot check very heterogeneous, perhaps also selective. No description of possible additional co-interventions. Small number of participants (n=12). | 4 |
| Lee et al. (2018) [24] | Single-arm | IHE:  Positive: 9 points  Negative: 6 points  Unclear: 4 points  Partial: 1 point | PRO: Cytologically type and severity of cancer described.  CONTRA: No information about side effects. No description of possible additional co-interventions. Unclear, if patients were recruited consecutively. Inclusion and exclusion criteria not clear for participants. Changes in blood vessels alone precludes estimating extent of increased blood perfusion in whole tumour mass. | 4 |
| Douwes et al. (2006) [25] | Single-arm | IHE:  Positive: 7 points  Negative: 5 points  Unclear: 4 points  Partial: 4 points | PRO: Detailly description of severity and metastases. Validation of the outcome (measurable lesions).  CONTRA: No information about funding or COI and about ethics approval. Results of development of weight and analgesics consumptions not reported. Unclear, if patients were recruited consecutively and no information about the period of time. No description of possible additional co-interventions. Inclusion and exclusion criteria not clear for participants. Spot check very heterogeneous, perhaps also selective. Questionable, if all side effects reported. | 4 |
| Gadaleta-Caldarola et al.  (2014) [26] | Single-arm | IHE:  Positive: 11 points  Negative: 5 points  Unclear: 3 points  Partial: 1 point | PRO: Validation of the outcome: lesions, proved by imaging.  CONTRA: No further information about funding or COI. No description of possible additional co-interventions. Unclear, if patients were recruited consecutively. | 4 |
| Wismeth et al.  (2010) [27] | Single-arm | IHE:  Positive: 10 points  Negative: 3 points  Unclear: 5 points  Partial: 2 points | PRO: Validation of the outcome: measurable lesion, proved by MRI. Prior treatment, cytologically type and severity of cancer described. Side effects detailly described. Reasons for end of study described.  CONTRA: Mistake in number of cumulated side effects (headache grade 3). Unclear, if patients were recruited consecutively. No information about the allocation to the four different treatment groups. No description of tumour volume at the beginning in the different groups. No description of possible additional co-interventions. Trend to less tolerance in group 3 and 4 not statistically substantiated. No further information about the COI. | 4 |
| Yoo et al. (2019) [28] | Single-arm | IHE:  Positive: 9 points  Negative: 4 points  Unclear: 5 points  Partial: 2 points | PRO: Validated questionnaire. Prior treatment, cytologically type and severity of cancer described.  CONTRA: Questionnaire at day 6 of treatment, only filled out of 37%. Several clinical data were missing (progression free survival, quality of life at some patients). No description of possible additional co-interventions. Unclear, if patients were recruited consecutively. | 4 |
| Lee et al. (2013) [29] | Single-arm | IHE:  Positive: 4 points  Negative: 8 points  Unclear: 5 points  Partial: 3 points | CONTRA: Methodology very dubious. Over 6 years, only 31 patients were included. Control arm contained only 8 participants. Allegedly arms are comparable according to the authors, but no comprehensible specification therefore can be found. No information about demographic values, prior treatment and severity of cancer.  Control arm contains only 1 woman, it seems to be very selective. No description of possible additional co-interventions. Unclear, if patients were recruited consecutively. No information about side effects. No information about COI, funding or about ethics approval. Due to the dubious methodology, the study does not count as a prospective, double arm, monocentric study, like it was conducted by the authors. Only the active arm was used for analysis like a single arm-study. | 4 |
| Yu et al. (2016) [30] | Single-arm | IHE:  Positive: 11 points  Negative: 2 points  Unclear: 4 points  Partial: 3 points | PRO: Validation of the outcome: clinical and physical examinations, HRQoL, VAS, chemistries and imaging. Number of participants for follow-up illustrated in a diagram according to consolidated standards of reporting trials. Prior treatment, cytologically type and severity of cancer describe. ANOVA used to determine changes in HRQoL.  CONTRA: Only 40% of the patients were followed as planned due to primarily patient refusal. True temperature of tumour area could not be assessed. HRQoL assessment: only a small proportion of enrolled patients due to loss to follow-up. No description of possible additional co-interventions. Unclear, if patients were recruited consecutively. Inaccurate description of side effects. | 4 |
| Heo et al. (2017) [31] | Single-arm | IHE:  Positive: 11 points  Negative: 4 points  Unclear: 3 points  Partial: 2 points | PRO: Validation of the outcome (lesions, proved by imaging). Prior treatment, cytologically type and severity of cancer described.  CONTRA: No description of possible additional co-interventions. Unclear, if patients were recruited consecutively. Inclusion and exclusion criteria not clear for participants. No information about COI or funding. | 4 |
| Ariyafar et al. (2019) [32] | Single-arm | IHE:  Positive: 5 points  Negative: 7 points  Unclear: 4 points  Partial: 4 points | PRO: Validated questionnaire. Questionnaire filled out at BL and 3 months after treatment.  CONTRA: Types of cancer, stage of cancer and prior therapies unclear. No information about target temperature, way of temperature measurement. No description of possible additional co-interventions. No report of adverse events. | 4 |
| Sahinbas et al. (2013) [33] | Case series | IHE:  Positive: 6 points  Negative: 8 points  Unclear: 3 points  Partial: 2 points  Not relevant: 1 point | PRO: Prior treatment and TNM-status described.  CONTRA: No information about period of time of the study. No information about target temperature, way of temperature measurement and further information about hyperthermia device. No description of possible additional co-interventions. Unclear, if patients were recruited consecutively. Inclusion and exclusion criteria not clear for participants. Spot check perhaps also selective. Very Small number of participants (n=4). No further information about funding. | 4 |
| ANOVA: analysis of variance. COI: conflict of Interests. CT: computed tomography. CTx: chemotherapy. ECC-WBH: extracorporeal circulation WBH. IHE: Institute of Health Economics-Quality Appraisal checklist for case series studies. MBS: modified Brunner-Score. MRI: magnetic resonance imaging. OS: overall survival. PD: progressive disease. HRQoL: health-related quality of life. QoL: quality of Life. RTx: radiotherapy. TNM: classification of malignant tumours (tumour, lymph nodes, metastasis). VAS: visual analogue scale. WBH: whole-body-hyperthermia. | | | | |

1. Atmaca A, Al-Batran S-E, Neumann A, et al. Whole-body hyperthermia (WBH) in combination with carboplatin in patients with recurrent ovarian cancer - a phase II study. Gynecologic oncology. 2009; 112(2): 384-8. https://dx.doi.org/10.1016/j.ygyno.2008.11.001.

2. Bakhshandeh A, Bruns I, Traynor A, et al. Ifosfamide, carboplatin and etoposide combined with 41.8 degrees C whole body hyperthermia for malignant pleural mesothelioma. Lung cancer (Amsterdam, Netherlands). 2003; 39(3): 339-45.

3. Bakshandeh-Bath A, Stoltz AS, Homann N, Wagner T, Stolting S, Peters SO. Preclinical and clinical aspects of carboplatin and gemcitabine combined with whole-body hyperthermia for pancreatic adenocarcinoma. Anticancer research. 2009; 29(8): 3069-77.

4. Bruns I, Kohlmann T, Wiedemann GJ, Bakhshandeh A. [Evaluation of the therapeutic benefit of 41.8 degrees C whole body hyperthermia plus ifosfamide, carboplatin and etoposide (ICE) for patients with malignant pleural mesothelioma using the Modified Brunner-Score (MBS)]. Bewertung des therapeutischen Gewinns von Patienten mit Pleuramesotheliom unter Therapie mit 41,8 degrees C Ganzkorperhyperthermie plus Ifosfamid, Carboplatin und Etoposid (ICE) anhand des Modifizierten Brunner-Score (MBS). 2004; 58(4): 210-6.

5. Katschinski DM, Wiedemann GJ, Mentzel M, Mulkerin DL, Touhidi R, Robins HI. Optimization of chemotherapy administration for clinical 41.8 degrees C whole body hyperthermia. Cancer letters. 1997; 115(2): 195‐199. https://dx.doi.org/10.1016/s0304-3835(97)04747-2.

6. Richel O, Zum Vorde Sive Vording PJ, Rietbroek R, et al. Phase II study of carboplatin and whole body hyperthermia (WBH) in recurrent and metastatic cervical cancer. Gynecologic oncology. 2004; 95(3): 680-5.

7. Westermann AM, Grosen EA, Katschinski DM, et al. A pilot study of whole body hyperthermia and carboplatin in platinum-resistant ovarian cancer. European journal of cancer (Oxford, England : 1990). 2001; 37(9): 1111-7.

8. Westermann AM, Wiedemann GJ, Jager E, et al. A Systemic Hyperthermia Oncologic Working Group trial. Ifosfamide, carboplatin, and etoposide combined with 41.8 degrees C whole-body hyperthermia for metastatic soft tissue sarcoma. Oncology. 2003; 64(4): 312-21.

9. Robins HI, Dennis WH, Neville AJ, et al. A nontoxic system for 41.8 degrees C whole-body hyperthermia: results of a Phase I study using a radiant heat device. Cancer research. 1985; 45(8): 3937-44.

10. Robins HI, Longo WL, Lagoni RK, et al. Phase I trial of lonidamine with whole body hyperthermia in advanced cancer. Cancer research. 1988; 48(22): 6587-92.

11. Robins HI, Longo WL, Steeves RA, et al. Adjunctive therapy (whole body hyperthermia versus lonidamine) to total body irradiation for the treatment of favorable B-cell neoplasms: a report of two pilot clinical trials and laboratory investigations. International journal of radiation oncology, biology, physics. 1990; 18(4): 909‐20. https://dx.doi.org/10.1016/0360-3016(90)90416-h.

12. Robins HI, Cohen JD, Schmitt CL, et al. Phase I clinical trial of carboplatin and 41.8 degrees C whole-body hyperthermia in cancer patients. Journal of clinical oncology : official journal of the American Society of Clinical Oncology. 1993; 11(9): 1787-94.

13. Bull JMC, Scott GL, Strebel FR, et al. Fever-range whole-body thermal therapy combined with cisplatin, gemcitabine, and daily interferon-alpha: a description of a phase I-II protocol. International journal of hyperthermia : the official journal of European Society for Hyperthermic Oncology, North American Hyperthermia Group. 2008; 24(8): 649-62. https://dx.doi.org/10.1080/02656730802104740.

14. Kraybill WG, Olenki T, Evans SS, et al. A phase I study of fever-range whole body hyperthermia (FR-WBH) in patients with advanced solid tumours: correlation with mouse models. International journal of hyperthermia : the official journal of European Society for Hyperthermic Oncology, North American Hyperthermia Group. 2002; 18(3): 253-66.

15. Steinhart CR, Ash SR, Gingrich C, Sapir D, Keeling GN, Yatvin MB. Effect of whole-body hyperthermia on AIDS patients with Kaposi's sarcoma: a pilot study. Journal of acquired immune deficiency syndromes and human retrovirology : official publication of the International Retrovirology Association. 1996; 11(3): 271-81.

16. Bull JM, Lees D, Schuette W, et al. Whole body hyperthermia: a phase-I trial of a potential adjuvant to chemotherapy. Annals of internal medicine. 1979; 90(3): 317-23.

17. Barlogie B, Corry PM, Yip E, et al. Total-body hyperthermia with and without chemotherapy for advanced human neoplasms. Cancer research. 1979; 39(5): 1481-9.

18. Gerad H, van Echo DA, Whitacre M, et al. Doxorubicin, cyclophosphamide, and whole body hyperthermia for treatment of advanced soft tissue sarcoma. Cancer. 1984; 53(12): 2585-91.

19. Locker GJ, Fuchs E-M, Worel N, et al. Whole body hyperthermia by extracorporeal circulation in spontaneously breathing sarcoma patients: hemodynamics and oxygen metabolism. The International journal of artificial organs. 2011; 34(11): 1085-94. https://dx.doi.org/10.5301/ijao.5000009.

20. Worel N, Knobl P, Karanikas G, et al. Hepatic dysfunction contributes to coagulation disturbances in patients undergoing whole body hyperthermia by use of extracorporeal circulation. International journal of artificial organs. 2014; 37(9): 715‐26. https://dx.doi.org/10.5301/ijao.5000350.

21. Koga S, Maeta M, Shimizu N, et al. Clinical effects of total-body hyperthermia combined with anticancer chemotherapy for far-advanced gastrointestinal cancer. Cancer. 1985; 55(8): 1641-7.

22. Wiedemann GJ, d'Oleire F, Knop E, et al. Ifosfamide and carboplatin combined with 41.8 degrees C whole-body hyperthermia in patients with refractory sarcoma and malignant teratoma. Cancer research. 1994; 54(20): 5346-50.

23. Wiedemann GJ, Robins HI, Gutsche S, et al. Ifosfamide, carboplatin and etoposide (ICE) combined with 41.8 degrees C whole body hyperthermia in patients with refractory sarcoma. European journal of cancer (Oxford, England : 1990). 1996; 32A(5): 888-92.

24. Lee S-Y, Kim J-H, Han Y-H, Cho D-H. The effect of modulated electro-hyperthermia on temperature and blood flow in human cervical carcinoma. International journal of hyperthermia : the official journal of European Society for Hyperthermic Oncology, North American Hyperthermia Group. 2018; 34(7): 953-960. https://dx.doi.org/10.1080/02656736.2018.1423709.

25. Douwes FR. Thermo-chemotherapy of the advanced pancreas carcinoma. Biologische Medizin. 2006; 35(3-4): 126-130.

26. Gadaleta-Caldarola G, Infusino S, Galise I, et al. Sorafenib and locoregional deep electro-hyperthermia in advanced hepatocellular carcinoma: A phase II study. Oncology Letters. 2014; 8(4): 1783-7. http://dx.doi.org/10.3892/ol.2014.2376.

27. Wismeth C, Dudel C, Pascher C, et al. Transcranial electro-hyperthermia combined with alkylating chemotherapy in patients with relapsed high-grade gliomas: phase I clinical results. Journal of neuro-oncology. 2010; 98(3): 395-405. https://dx.doi.org/10.1007/s11060-009-0093-0.

28. Yoo HJ, Lim MC, Seo S-S, Kang S, Park S-Y, Joo J. Phase I/II clinical trial of modulated electro-hyperthermia treatment in patients with relapsed, refractory or progressive heavily treated ovarian cancer. Japanese Journal of Clinical Oncology. 2019; 49(9): 832-8. http://dx.doi.org/10.1093/jjco/hyz071.

29. Lee DY, Haam SJ, Kim TH, Lim JY, Kim EJ, Kim NY. Oncothermia with Chemotherapy in the Patients with Small-Cell Lung Cancer. Conference Papers in Medicine. 2013; https://dx.doi.org/10.1155/2013/910363.

30. Yu JI, Park HC, Choi DH, et al. Prospective phase II trial of regional hyperthermia and whole liver irradiation for numerous chemorefractory liver metastases from colorectal cancer. Radiation oncology journal. 2016; 34(1): 34-44. https://dx.doi.org/10.3857/roj.2016.34.1.34.

31. Heo J, Kim SH, Oh YT, Chun M, Noh OK. Concurrent hyperthermia and re-irradiation for recurrent high-grade gliomas. Neoplasma. 2017; 64(5): 803-8. http://dx.doi.org/10.4149/neo_2017_520.

32. Ariyafar T, Geraily G, Shirazi A, et al. Evaluating the effectiveness of combined radiotherapy and hyperthermia for the treatment response of patients with painful bony metastases: A phase 2 clinical trial. Journal of Thermal Biology. 2019; 84: 129-35. http://dx.doi.org/10.1016/j.jtherbio.2019.06.003.

33. Sahinbas H, Wehner H. Combined deep hyperthermia and chemotherapy with 5-fluorouracil/sodium folinate and mitomycin in previously treated patients with inoperable colorectal liver metastases - A case study. Deutsche Zeitschrift fur Onkologie. 2013; 45(2): 74-7. http://dx.doi.org/10.1055/s-0033-1334375.
