# Supplementary material for: Systematic review about complementary medical hyperthermia in oncology
Source: Clin Exp Med. 2022 Jun 29;22(4):519–65. doi: 10.1007/s10238-022-00846-9 (PMC9244386; doi:10.1007/s10238-022-00846-9)
Supplement: Supplementary file 3 — Supplementary file3 (DOCX 235 kb) [file 10238_2022_846_MOESM3_ESM.docx]

Supplementary table 3: Excluded studies after fulltext screening

| **Reference:** | **Reason for exclusion:** |
| --- | --- |
| Mandala et al. (2019) [1] | Another publication type (abstract) |
| Cheng et al. (2019) [2] | Another publication type (basic literature) |
| Hildebrandt et al. (2005) [3] | Another publication type (basic literature) |
| Szasz et al. (2014) [4] | Another publication type (basic literature) |
| Szasz et al. (2019) [5] | Another publication type (basic literature) |
| Ziske et al. (2002) [6] | Another publication type (basic literature) |
| Sauer et al. (2021) [7] | Another publication type (comment) |
| Ranieri et al. (2020) [8] | Another publication type (corrigendum) |
| Van der Horst et al. (2018) [9] | Another publication type (no systematic review and different kind of hyperthermia treatments mixed) |
| Reymond et al. (2020) [10] | Another publication type (editorial) |
| Lagendijk et al. (1998) [11] | Another publication type (guideline) |
| Chen et al. (2020) [12] | Another publication type (letter to the editor) |
| Notter et al. (2021) [13] | Another publication type (letter to the editor) |
| Roussakow et al. (2019) [14] | Another publication type (Letter to the editor) |
| Datta et al. (2015) [15] | Another publication type (meeting report) |
| Datta et al. (2019) [16] | Another publication type (meeting report) |
| Stutz et al. (2019) [17] | Another publication type (meeting report) |
| Stutz et al. (2019) [18] | Another publication type (meeting report) |
| Gao et al. (2019) [19] | Another publication type (poster) |
| Jiang et al. (2019) [20] | Another publication type (poster) |
| Minnaar et al. (2019) [21] | Another publication type (poster) |
| Nichols et al. (2019) [22] | Another publication type (poster) |
| Osong et al. (2019) [23] | Another publication type (poster) |
| Wang et al. (2019) [24] | Another publication type (poster) |
| Wang et al. (2020) [25] | Another publication type (poster) |
| You et al. (2020) [26] | Another publication type (poster) |
| Mansmann et al. (2019) [27] | Another publication type (reply) |
| Ding et al. (2020) [28] | Different language (not English or German) |
| Ishikawa et al. (2021) [29] | Different language (not English or German) |
| Jin et al.(2020) [30] | Different language (not English or German) |
| Xie et al. (2013) [31] | Different language (not English or German) |
| Yu et al. (2016) [32] | Different language (not English or German) |
| Aker et al. (2020) [33] | Fulltext not available |
| Chen et al. (1996) [34] | Fulltext not available |
| Du et al. (2010) [35] | Fulltext not available |
| Kim et al. (2019) [36] | Fulltext not available |
| Li et al. (2015) [37] | Fulltext not available |
| Mi et al. (2011) [38] | Fulltext not available |
| Qi et al. (2013) [39] | Fulltext not available |
| Shi et al. (2012) [40] | Fulltext not available |
| Wang et al. (2012) [41] | Fulltext not available |
| Zhao et al. (2016) [42] | Fulltext not available |
| Nct et al. (2004) [43] | Fulltext not available, study closed |
| Aiba et al. (2019) [44] | Hyperthermia, but not complementary medicine |
| Alonso-Gomez et al. (2019) [45] | Hyperthermia, but not complementary medicine |
| Arslan et al. (2017) [46] | Hyperthermia, but not complementary medicine |
| Atmaca et al. (2009) [47] | Hyperthermia, but not complementary medicine |
| Bailey et al. (2019) [48] | Hyperthermia, but not complementary medicine |
| Bakker et al. (2019) [49] | Hyperthermia, but not complementary medicine |
| Beck et al. (2021) [50] | Hyperthermia, but not complementary medicine |
| Bucklein et al. (2020) [51] | Hyperthermia, but not complementary medicine |
| Byun et al. (2019) [52] | Hyperthermia, but not complementary medicine |
| Carina et al. (2019) [53] | Hyperthermia, but not complementary medicine |
| Chen et al. (2020) [54] | Hyperthermia, but not complementary medicine |
| Chi (2020) [55] | Hyperthermia, but not complementary medicine |
| Ciampa et al. (2020) [56] | Hyperthermia, but not complementary medicine |
| Creeze et al. (2019) [57] | Hyperthermia, but not complementary medicine |
| Datta et al. (2016) [58] | Hyperthermia, but not complementary medicine |
| Datta et al. (2016) [59] | Hyperthermia, but not complementary medicine |
| Datta et al. (2018) [60] | Hyperthermia, but not complementary medicine |
| Datta et al. (2019) [61] | Hyperthermia, but not complementary medicine |
| Datta et al. (2019) [62] | Hyperthermia, but not complementary medicine |
| De Boer et al. (2020) [63] | Hyperthermia, but not complementary medicine |
| De Haas et al. (2006) [64] | Hyperthermia, but not complementary medicine |
| De-Colle et al. (2019) [65] | Hyperthermia, but not complementary medicine |
| De-Graaf-Strukowska et al. (1999) [66] | Hyperthermia, but not complementary medicine |
| Delhorme et al. (2020) [67] | Hyperthermia, but not complementary medicine |
| Dharmaiah et al. (2019) [68] | Hyperthermia, but not complementary medicine |
| Djokic et al. (2020) [69] | Hyperthermia, but not complementary medicine |
| Eberle et al. (2019) [70] | Hyperthermia, but not complementary medicine |
| El Sharouni et al. (1997) [71] | Hyperthermia, but not complementary medicine |
| Evrad et al. (2019) [72] | Hyperthermia, but not complementary medicine |
| Fang et al. (2019) [73] | Hyperthermia, but not complementary medicine |
| Franchi et al. (2007) [74] | Hyperthermia, but not complementary medicine |
| Franckena et al. (2008) [75] | Hyperthermia, but not complementary medicine |
| Franckena et al. (2009) [76] | Hyperthermia, but not complementary medicine |
| Gani et al. (2016) [77] | Hyperthermia, but not complementary medicine |
| Gani et al. (2019) [78] | Hyperthermia, but not complementary medicine |
| Ganul et al. (1999) [79] | Hyperthermia, but not complementary medicine |
| Garcia et al. (2019) [80] | Hyperthermia, but not complementary medicine |
| Guo et al. (2007) [81] | Hyperthermia, but not complementary medicine |
| Hamza et al. (2021) [82] | Hyperthermia, but not complementary medicine |
| Harima et al. (2000) [83] | Hyperthermia, but not complementary medicine |
| Harima et al. (2001) [84] | Hyperthermia, but not complementary medicine |
| Harima et al. (2009) [85] | Hyperthermia, but not complementary medicine |
| Harima et al. (2016) [86] | Hyperthermia, but not complementary medicine |
| He et al. (2019) [87] | Hyperthermia, but not complementary medicine |
| Hendricks et al. (2021) [88] | Hyperthermia, but not complementary medicine |
| Hendricksen et al. (2019) [89] | Hyperthermia, but not complementary medicine |
| Hu et al. (2017) [90] | Hyperthermia, but not complementary medicine |
| Huilgol et al. (2006) [91] | Hyperthermia, but not complementary medicine |
| Huilgol et al. (2010) [92] | Hyperthermia, but not complementary medicine |
| Hurwitz et al. (2002) [93] | Hyperthermia, but not complementary medicine |
| Ichikawa et al. (1996) [94] | Hyperthermia, but not complementary medicine |
| Iizumi et al. (2019) [95] | Hyperthermia, but not complementary medicine |
| Ishikawa et al. (2012) [96] | Hyperthermia, but not complementary medicine |
| Issels et al. (2020) [97] | Hyperthermia, but not complementary medicine |
| Iwahashi et al. (1999) [98] | Hyperthermia, but not complementary medicine |
| Jones et al. (2003) [99] | Hyperthermia, but not complementary medicine |
| Jow et al. (2019) [100] | Hyperthermia, but not complementary medicine |
| Kamisawa et al. (2005) [101] | Hyperthermia, but not complementary medicine |
| Kang et al. (2011) [102] | Hyperthermia, but not complementary medicine |
| Kato et al. (2014) [103] | Hyperthermia, but not complementary medicine |
| Kim et al. (2018) [104] | Hyperthermia, but not complementary medicine |
| Kitamura et al. (1998) [105] | Hyperthermia, but not complementary medicine |
| Kok et al. (2020) [106] | Hyperthermia, but not complementary medicine |
| Kok et al.(2020) [107] | Hyperthermia, but not complementary medicine |
| Kong et al. (2017) [108] | Hyperthermia, but not complementary medicine |
| Kouloulias et al. (2015) [109] | Hyperthermia, but not complementary medicine |
| Kroesen et al. (2019) [110] | Hyperthermia, but not complementary medicine |
| Kroesen et al.(2019) [111] | Hyperthermia, but not complementary medicine |
| Kuwano et al. (1995) [112] | Hyperthermia, but not complementary medicine |
| Lamprecht et al. (2021) [113] | Hyperthermia, but not complementary medicine |
| Letica-Kriegel et al. (2020) [114] | Hyperthermia, but not complementary medicine |
| Lindner et al. (2019) [115] | Hyperthermia, but not complementary medicine |
| Lindner et al. (2021) [116] | Hyperthermia, but not complementary medicine |
| Linthorst et al. (2015) [117] | Hyperthermia, but not complementary medicine |
| Liu et al. (2019) [118] | Hyperthermia, but not complementary medicine |
| Liu et al. (2020) [119] | Hyperthermia, but not complementary medicine |
| Lloret et al. (2019) [120] | Hyperthermia, but not complementary medicine |
| Lutgens et al. (2007) [121] | Hyperthermia, but not complementary medicine |
| Lyu et al. (2014) [122] | Hyperthermia, but not complementary medicine |
| Ma et al. (2019) [123] | Hyperthermia, but not complementary medicine |
| Maluta et al. (2007) [124] | Hyperthermia, but not complementary medicine |
| Maluta et al. (2011)[125] | Hyperthermia, but not complementary medicine |
| Merten et al. [126] (2019) | Hyperthermia, but not complementary medicine |
| Mitsumori et al. (2007) [127] | Hyperthermia, but not complementary medicine |
| Mittal et al. (1996) [128] | Hyperthermia, but not complementary medicine |
| Nakajima et al. (2015) [129] | Hyperthermia, but not complementary medicine |
| Nozato et al. (2019) [130] | Hyperthermia, but not complementary medicine |
| Nozoe et al. (1996) [131] | Hyperthermia, but not complementary medicine |
| Oberacker et al. (2020) [132] | Hyperthermia, but not complementary medicine |
| Ohguri et al. (2008) [133] | Hyperthermia, but not complementary medicine |
| Okamoto et al. (1996) [134] | Hyperthermia, but not complementary medicine |
| Oldenborg et al. (2019) [135] | Hyperthermia, but not complementary medicine |
| Osone et al. (2021) [136] | Hyperthermia, but not complementary medicine |
| Ostapenko et al. (2005) [137] | Hyperthermia, but not complementary medicine |
| Ott et al. (2019) [138] | Hyperthermia, but not complementary medicine |
| Ott et al. (2021) [139] | Hyperthermia, but not complementary medicine |
| Reitjenbagh et al. (2020) [140] | Hyperthermia, but not complementary medicine |
| Rizzo et al. (1998) [141] | Hyperthermia, but not complementary medicine |
| Rogers et al. (2021) [142] | Hyperthermia, but not complementary medicine |
| Saeki et al. (1998) [143] | Hyperthermia, but not complementary medicine |
| Sakurai et al. (2002) [144] | Hyperthermia, but not complementary medicine |
| Sano et al. (2019) [145] | Hyperthermia, but not complementary medicine |
| Schaffer et al. (2003) [146] | Hyperthermia, but not complementary medicine |
| Schullian et al. (2019) [147] | Hyperthermia, but not complementary medicine |
| Shimomura et al. (2021) [148] | Hyperthermia, but not complementary medicine |
| Silva et al. (2010) [149] | Hyperthermia, but not complementary medicine |
| Sneed et al. (1996) [150] | Hyperthermia, but not complementary medicine |
| Spalek et al. (2021) [151] | Hyperthermia, but not complementary medicine |
| Sridhar et al. (2019) [152] | Hyperthermia, but not complementary medicine |
| Stoetzer et al. (2020) [153] | Hyperthermia, but not complementary medicine |
| Stoetzer et al. (2021) [154] | Hyperthermia, but not complementary medicine |
| Striefler et al. (2020) [155] | Hyperthermia, but not complementary medicine |
| Sugarbaker et al. (2019) [156] | Hyperthermia, but not complementary medicine |
| Sugerbaker et al. (2019) [157] | Hyperthermia, but not complementary medicine |
| Tran et al. (2020) [158] | Hyperthermia, but not complementary medicine |
| Tsutsumi et al. (2011) [159] | Hyperthermia, but not complementary medicine |
| Unsoeld et al. (2020) [160] | Hyperthermia, but not complementary medicine |
| Van Vulpen et al. (2003) [161] | Hyperthermia, but not complementary medicine |
| Wang et al. (2013) [162] | Hyperthermia, but not complementary medicine |
| Yang et al. (2019) [163] | Hyperthermia, but not complementary medicine |
| Yang et al. (2020) [164] | Hyperthermia, but not complementary medicine |
| Zagar et al. (2014) [165] | Hyperthermia, but not complementary medicine |
| Zhao et al. (2019) [166] | Hyperthermia, but not complementary medicine |
| Zhou et al. (2019) [167] | Hyperthermia, but not complementary medicine |
| Zwirner et al. (2018) [168] | Hyperthermia, but not complementary medicine |
| Datta et al. (2019) [169] | Preclilnical study |
| Joiner et al. (2020) [170] | Preclilnical study |
| Thomsen et al. (2020) [171] | Preclilnical study |
| Miao et al. (2020) [172] | Preclinical study |
| Feyerabend et al. (1997) [173] | Topic not related to hyerthermia |
| Abdel-Rahman et al. (2018) [174] | Topic not related to hyperthermia |
| Aquilina et al. (2020) [175] | Topic not related to hyperthermia |
| Bogovic et al. (2001) [176] | Topic not related to hyperthermia |
| Depla et al. (2014) [177] | Topic not related to hyperthermia |
| Furness et al. (2011) [178] | Topic not related to hyperthermia |
| Goyal et al. (2021) [179] | Topic not related to hyperthermia |
| Kim et al. (2019) [180] | Topic not related to hyperthermia |
| Klos et al. (2019) [181] | Topic not related to hyperthermia |
| Lin et al. (2019) [182] | Topic not related to hyperthermia |
| Rim et al. (2021) [183] | Topic not related to hyperthermia |
| Schaper et al. (2020) [184] | Topic not related to hyperthermia |
| Schreckenbach (2019) [185] | Topic not related to hyperthermia |
| Villanueva et al. (2019) [186] | Topic not related to hyperthermia |
| Xu et al. (2016)[187] | Topic not related to hyperthermia |
| Zhang et al. (2020) [188] | Topic not related to hyperthermia |

1. Mandala M, Dummer R, Ascierto PA, et al. Characteristics of pyrexia with encorafenib (ENCO) plus binimetinib (BINI) in patients with BRAF-mutant melanoma. Pigment cell & melanoma research. 2019; https://dx.doi.org/10.1111/pcmr.12738.

2. Cheng Y, Weng S, Yu L, Zhu N, Yang M, Yuan Y. The Role of Hyperthermia in the Multidisciplinary Treatment of Malignant Tumors. Integrative cancer therapies. 2019; https://dx.doi.org/10.1177/1534735419876345.

3. Hildebrandt B, Hegewisch-Becker S, Kerner T, et al. Current status of radiant whole-body hyperthermia at temperatures >41.5degreeC and practical guidelines for the treatment of adults. The German 'Interdisciplinary Working Group on Hyperthermia'. International Journal of Hyperthermia. 2005; 21(2): 169-83. http://dx.doi.org/10.1080/02656730400003401.

4. Szasz A. Current status of oncothermia therapy for lung cancer. The Korean journal of thoracic and cardiovascular surgery. 2014; 47(2): 77-93. http/dx.doi.org/10.5090/kjtcs.2014.47.2.77.

5. Szasz AM, Minnaar CA, Szentmartoni G, Szigeti GP, Dank M. Review of the Clinical Evidences of Modulated Electro-Hyperthermia (mEHT) Method: An Update for the Practicing Oncologist. Frontiers in oncology. 2019; 9: 1012. https://dx.doi.org/10.3389/fonc.2019.01012.

6. Ziske C. Is whole body hyperthermia plus chemotherapy useful in pancreatic carcinoma? Deutsche Medizinische Wochenschrift. 2002; 127(31-32): 1660. http://dx.doi.org/10.1055/s-2002-33205.

7. Sauer R, Creeze H, Hulshof M, et al. Concerning the final report "Hyperthermia: a systematic review" of the Ludwig Boltzmann Institute for Health Technology Assessment, Vienna, March 2010. Strahlentherapie und Onkologie : Organ der Deutschen Rontgengesellschaft ... [et al]. 2012; 188(3): 209-13. https://dx.doi.org/10.1007/s00066-012-0072-9.

8. Ranieri G, Laface C, Porcelli M, et al. Corrigendum: Bevacizumab Plus FOLFOX-4 Combined With Deep Electro-Hyperthermia as First-line Therapy in Metastatic Colon Cancer: A Pilot Study (Front. Oncol, (2020), 10, (590707), 10.3389/fonc.2020.590707). Frontiers in Oncology. 2020; http://dx.doi.org/10.3389/fonc.2020.637880.

9. van der Horst A, Versteijne E, Besselink MGH, et al. The clinical benefit of hyperthermia in pancreatic cancer: a systematic review. International journal of hyperthermia : the official journal of European Society for Hyperthermic Oncology, North American Hyperthermia Group. 2018; 34(7): 969-79. https://dx.doi.org/10.1080/02656736.2017.1401126.

10. Reymond MA, Pocard M. "peritoneal failure": A new concept to explain negative results of randomized trials evaluating intraperitoneal therapies. Pleura and Peritoneum. 2020; http://dx.doi.org/10.1515/pp-2020-0117.

11. Lagendijk JJW, Van Rhoon GC, Hornsleth SN, et al. ESHO quality assurance guidelines for regional hyperthermia. International Journal of Hyperthermia. 1998; 14(2): 125-33.

12. Chen H, Ma G, Wang X, Zhou W, Wang S. Time interval after heat stress plays an important role in the combination therapy of hyperthermia and cancer chemotherapy agents. International journal of hyperthermia : the official journal of European Society for Hyperthermic Oncology, North American Hyperthermia Group. 2020; 37(1): 254-5. https://dx.doi.org/10.1080/02656736.2020.1736343.

13. Notter M, Thomsen AR, Grosu A-L, Vaupel P. Recommendation of Regional Hyperthermia in the Treatment of Breast Cancer. Integrative cancer therapies. 2021; https://dx.doi.org/10.1177/1534735420988606.

14. Roussakow S. Regional Hyperthermia With Neoadjuvant Chemotherapy for Treatment of Soft Tissue Sarcoma. JAMA oncology. 2019; 5(1): 113-4. https://dx.doi.org/10.1001/jamaoncol.2018.5296.

15. Datta NR, Ordonez SG, Gaipl US, et al. Local hyperthermia combined with radiotherapy and-/or chemotherapy: recent advances and promises for the future. Cancer treatment reviews. 2015; 41(9): 742-53. https://dx.doi.org/10.1016/j.ctrv.2015.05.009.

16. Datta NR, Stutz E, Gomez S, Bodis S. Systematic review & network meta-analysis to identify the optimum nonoperative therapeutic strategies in locally advanced cancer cervix. Strahlentherapie und Onkologie. 2019; 195(6): 580-1. http://dx.doi.org/10.1007/s00066-019-01459-0.

17. Stutz E, Puric E, Meister A, et al. First case report of concurrent proton therapy with hyperthermia in a large extra-abdominal desmoid tumor. Strahlentherapie und Onkologie. 2019; 195(6): 581-2. http://dx.doi.org/10.1007/s00066-019-01459-0.

18. Stutz E, Eberle B, Puric E, et al. Thermo-radiotherapy in bladder cancer is a promising option in patients unfit for cystectomy or chemo-radiotherapy. Strahlentherapie und Onkologie. 2019; 195(6): 583. http://dx.doi.org/10.1007/s00066-019-01459-0.

19. Gao XS, Van Doorn HC, Boere IA, et al. Acute and long-term toxicity in patients undergoing induction chemotherapy followed by radiotherapy and hyperthermia for advanced cervical cancer. International Journal of Gynecological Cancer. 2019; 29(Supplement 4): A276. http://dx.doi.org/10.1136/ijgc-2019-ESGO.484.

20. Jiang P, Qu A, Shao Y, Wang J, Sun W. Radical CCRT Combined Thermotherapy for Ib2, IIa and Local Advanced Bulky Cervical Cancer: A Single Center Retrospective Study. International Journal of Radiation Oncology, Biology, Physics. 2019; 105: 323-4. http/dx.doi.org/10.1016/j.ijrobp.2019.06.1807.

21. Minnaar CA, Kotzen JA. Modulated electro hyperthermia as an immune modulator with checkpoint inhibitors and radiotherapy. European Journal of Cancer. 2019; 110(Supplement 1): 19-20. http://dx.doi.org/10.1016/j.ejca.2019.01.068.

22. Nichols EM, Kowalski ES, Remick J, Mishra MV. Short-Term Outcomes of Re-Irradiation to the Chest Wall Using Intensity-Modulated Proton Therapy (IMPT) in Women with Breast Cancer. International Journal of Radiation Oncology Biology Physics. 2019; 105(1 Supplement): 5-6. http://dx.doi.org/10.1016/j.ijrobp.2019.06.628.

23. Osong ABA. Development of a nomogram for predicting overall survival in patients with Cervical cancer. Radiotherapy and Oncology. 2019; 133(Supplement 1). http://dx.doi.org/10.1016/S0167-8140%2819%2931253-8.

24. Wang Y, Chen H. 15-year survival and toxicity of locally advanced cervical cancer treatment with hyperthermia in combination with radiochemotherapy. International Journal of Gynecological Cancer. 2019; 29(Supplement 4): 279. http://dx.doi.org/10.1136/ijgc-2019-ESGO.490.

25. Wang Y, Chen H. Hyperthermia Combined with Concurrent Radiochemotherapy for 373 Patients with Cervical Cancer Stage IB-IV. International Journal of Radiation Oncology Biology Physics. 2020; 108(3 Supplement): 112. http://dx.doi.org/10.1016/j.ijrobp.2020.07.2301.

26. You SH, Kim S. Feasibility of modulated electro-hyperthermia in preoperative treatment for locally advanced rectal cancer: Early phase 2 clinical results. Neoplasma. 2020; 67(3): 677-83. https://dx.doi.org/10.4149/neo_2020_190623N538.

27. Mansmann U, Lindner LH, Issels R. Regional Hyperthermia With Neoadjuvant Chemotherapy for Treatment of Soft Tissue Sarcoma-Reply. JAMA oncology. 2019; 5(1): 114. https://dx.doi.org/10.1001/jamaoncol.2018.5293.

28. Ding PA, Liu Y, Guo HH, et al. Application of laparoscopic exploration combined with abdominal exfoliative cytology in the diagnosis and treatment of locally advanced gastric cancer. Zhonghua wei chang wai ke za zhi [Chinese journal of gastrointestinal surgery]. 2020; 23(2): 170‐6. http/dx.doi.org/10.3760/cma.j.issn.1671-0274.2020.02.013.

29. Ishikawa H, Kouki T, Oda R, et al. Neoadjuvant Hyperthermia and Chemoradiotherapy for Borderline Resectable Pancreatic Cancer. Gan to kagaku ryoho. Cancer & chemotherapy. 2021; 48(3): 388-90.

30. The Efficacy of Hyperthermia Combined with Radiotherapy in the Treatment of Advanced Gastric Cancer. Anti-tumor pharmacy. 2020; 10(3): 320‐3. http/dx.doi.org/10.3969/j.issn.2095-1264.2020.03.10.

31. Xie BP, Li Z, Yao N, He ZY. Chemotherapy combined with hyperthermia for advanced colorectal cancer: A meta-analysis. Chinese Journal of Evidence-Based Medicine. 2013; 13(3): 352-7. http://dx.doi.org/10.7507/1672-2531.20130060.

32. Yu X, Li X, Zhou J, et al. Different adjuvant chemotherapies for the retreatment patients with advanced primary hepatic carcinoma: an efficacy and survival analysis. Cancer research and clinic. 2016; 28(9): 608‐10 and 615. http/dx.doi.org/10.3760/cma.j.issn.1006-9801.2016.09.008.

33. Aker SS, Mantiero M. Systemic treatment of recurrent ovarian cancer. International Journal of Gynecological Cancer. 2020; 30(Supplement 1): 7. http://dx.doi.org/10.1136/ijgc-30-S1.

34. Chen HW, Fan JJ, Yang SY. The therapeutic effect of thermotherapy, radiotherapy and chemotherapy on uterocervical carcinoma and its electron microscope alteration. Chinese journal of physical therapy. 1996; 19(4): 216‐9.

35. Du CJ, Wang TL, Guo YS. Clinical observation of quality of life in patients with gastric carcinoma treated by combination of tradition Chinese medicine, chemotherapy and hyperthermia treatment. Hebei journal of traditional chinese medicine [ he bei zhong yi ]. 2010; 32(9): 1299‐301.

36. Kim K, Suh DH, No JH, et al. A phase I trial evaluating the safety and efficacy of weekly paclitaxel or cisplatin with electro-hyperthermia in patients with recurrent or persistent epithelial ovarian, fallopian tubal or primary peritoneal carcinoma (KGOG 3030). Journal of clinical oncology. 2019; 37(15).

37. Li N, Li Y, Gong P, Li J, Wang YL. Clinical effects of hyperthermia combined with S-1 chemotherapy in elderly patients with advanced gastric cancer. World chinese journal of digestology. 2015; 23(3): 445‐50. http/dx.doi.org/10.11569/wcjd.v23.i3.445.

38. Mi DH, Li Z, Yang KH, Tian JH, Wang DY. HRCT for non-small cell lung cancer: A meta-analysis. Chinese Journal of Evidence-Based Medicine. 2011; 11(11): 1262-7.

39. Qi JD, Guo BC. Clinical observation of local hypeythermia combined with best support treatments on cancer cachexia of digestive tract. Journal of practical oncology. 2013; 28(1): 53‐6.

40. Shi XY, Zhou TC, Lin XD. Comparison of chemoradiation combined with hyperthermia therapy and chemoradiation therapy alone in invasive cervical cancer: a perspective randomized controlled study. Journal of practical oncology. 2012; 27(4): 386‐90.

41. Wang Dy, Li Z, Yang Kh, Tian Jh, Zhang Qn, Wang Xh. Radiotherapy combined with hyperthermia for locally-advanced non-small cell lung cancer: A systematic review. Chinese Journal of Evidence-Based Medicine. 2012; 12(10): 1203-8.

42. Zhao Q, Diao J, Wen Q, Song L, Ren Y. Short-term effect of hyperthermia combined with concurrent radiochemotherapy on middle stage and advanced cervical cancer. Cancer research and clinic. 2016; 28(8): 546‐8.

43. Cisplatin and Radiation Therapy With or Without Hyperthermia Therapy in Treating Patients With Cervical Cancer. Nct. 2004. https://clinicaltrials.gov/show/NCT00085631. Accessed 27 March 2022.

44. Aiba H, Hayashi K, Yamada S, et al. Treatment of a Malignant Soft Tissue Tumor Arising in the Vicinity of the Sciatic Nerve with an In-Situ Preparation Technique and Intensive Multidisciplinary Therapy. Cancers. 2019; https://dx.doi.org/10.3390/cancers11040506.

45. Alonso-Gomez J, Silvestre-Rodriguez J, Bermejo-Guillen MI, Artiles-Armas M, Marchena-Gomez J. First PIPAC treatment in a Spanish public hospital: A novel technique for the treatment of carcinomatosis. Primer tratamiento PIPAC en un hospital publico espanol. Una novedosa tecnica para el tratamiento de la carcinomatosis. 2019; 97(3): 181-2. https://dx.doi.org/10.1016/j.ciresp.2018.08.007.

46. Arslan SA, Ozdemir N, Sendur MA, et al. Hyperthermia and radiotherapy combination for locoregional recurrences of breast cancer: A review. Breast Cancer Management. 2017; 6(4): 117-26. http://dx.doi.org/10.2217/bmt-2017-0011.

47. Atmaca A, Al-Batran S-E, Neumann A, et al. Whole-body hyperthermia (WBH) in combination with carboplatin in patients with recurrent ovarian cancer - a phase II study. Gynecologic oncology. 2009; 112(2): 384-8. https://dx.doi.org/10.1016/j.ygyno.2008.11.001.

48. Bailey RE, Core J, Cortizo Vidal LL, et al. Safety and efficacy of locoregional therapy for metastatic pancreatic ductal adenocarcinoma to the liver: A single-center experience. Journal of Gastrointestinal Oncology. 2019; 10(4): 688-94. http://dx.doi.org/10.21037/jgo.2019.03.13.

49. Bakker A, van der Zee J, van Tienhoven G, Kok HP, Rasch CRN, Crezee H. Temperature and thermal dose during radiotherapy and hyperthermia for recurrent breast cancer are related to clinical outcome and thermal toxicity: a systematic review. International journal of hyperthermia : the official journal of European Society for Hyperthermic Oncology, North American Hyperthermia Group. 2019; 36(1): 1024-39. https://dx.doi.org/10.1080/02656736.2019.1665718.

50. Beck M, Ghadjar P, Mehrhof F, et al. Salvage-Radiation Therapy and Regional Hyperthermia for Biochemically Recurrent Prostate Cancer after Radical Prostatectomy (Results of the Planned Interim Analysis). Cancers. 2021; https://dx.doi.org/10.3390/cancers13051133.

51. Bucklein V, Limmroth C, Kampmann E, et al. Ifosfamide, Carboplatin, and Etoposide (ICE) in Combination with Regional Hyperthermia as Salvage Therapy in Patients with Locally Advanced Nonmetastatic and Metastatic Soft-Tissue Sarcoma. Sarcoma. 2020; https://dx.doi.org/10.1155/2020/6901678.

52. Byun YH, Gwak HS, Kwon J-W, et al. Local recurrence of brain metastasis reduced by intra-operative hyperthermia treatment. International journal of hyperthermia : the official journal of European Society for Hyperthermic Oncology, North American Hyperthermia Group. 2019; 35(1): 168-75. https://dx.doi.org/10.1080/02656736.2018.1488004.

53. Carina V, Costa V, Sartori M, et al. Adjuvant Biophysical Therapies in Osteosarcoma. Cancers. 2019; https://dx.doi.org/10.3390/cancers11030348.

54. Chen C, Wang J, Zhao Y, et al. Factors Prognostic for Peritoneal Metastases from Colorectal Cancer Treated with Surgery. Cancer management and research. 2020; 12: 10587-602. <https://dx.doi.org/10.2147/CMAR.S270830>.

55. Chi MS, Ko HL, Huang YY, et al. Adding Hyperthermia To Salvage Concurrent Chemoradiotherapy For Previously Irradiated Unresectable Recurrent Head And Neck Cancer: A Phase II Clinical Trial. International Journal of Radiation Oncology Biology Physics. 2020; 108(3 Supplement): 790. http://dx.doi.org/10.1016/j.ijrobp.2020.07.262.

56. Ciampa ML, Chohonis JP, Otto RS, Franklin BT. Invasive Mucinous Neoplasm of the Appendix Masquerading as Recurrent Urinary Tract Infections: a Case Report. Military medicine. 2020; 185(11-12): 2166-70. https://dx.doi.org/10.1093/milmed/usaa179.

57. Crezee H, Kok HP, Oei AL, Franken NAP, Stalpers LJA. The Impact of the Time Interval Between Radiation and Hyperthermia on Clinical Outcome in Patients With Locally Advanced Cervical Cancer. Frontiers in oncology. 2019; 9: 412. https://dx.doi.org/10.3389/fonc.2019.00412.

58. Datta NR, Rogers S, Ordonez SG, Puric E, Bodis S. Hyperthermia and radiotherapy in the management of head and neck cancers: A systematic review and meta-analysis. International journal of hyperthermia : the official journal of European Society for Hyperthermic Oncology, North American Hyperthermia Group. 2016; 32(1): 31-40. https://dx.doi.org/10.3109/02656736.2015.1099746.

59. Datta NR, Rogers S, Klingbiel D, Gomez S, Puric E, Bodis S. Hyperthermia and radiotherapy with or without chemotherapy in locally advanced cervical cancer: a systematic review with conventional and network meta-analyses. International journal of hyperthermia : the official journal of European Society for Hyperthermic Oncology, North American Hyperthermia Group. 2016; 32(7): 809-21. https://dx.doi.org/10.1080/02656736.2016.1195924.

60. Datta NR, Stutz E, Gomez S, Bodis S. Efficacy and Safety Evaluation of the Various Therapeutic Options in Locally Advanced Cervix Cancer: A Systematic Review and Network Meta-Analysis of Randomized Clinical Trials. International Journal of Radiation Oncology, Biology, Physics. 2018; https://dx.doi.org/10.1016/j.ijrobp.2018.09.037.

61. Datta NR, Stutz E, Gomez S, Bodis S. Efficacy and Safety Evaluation of the Various Therapeutic Options in Locally Advanced Cervix Cancer: A Systematic Review and Network Meta-Analysis of Randomized Clinical Trials. International journal of radiation oncology, biology, physics. 2019; 103(2): 411-37. https://dx.doi.org/10.1016/j.ijrobp.2018.09.037.

62. Datta NR, Stutz E, Puric E, et al. A Pilot Study of Radiotherapy and Local Hyperthermia in Elderly Patients With Muscle-Invasive Bladder Cancers Unfit for Definitive Surgery or Chemoradiotherapy. Frontiers in oncology. 2019; 9: 889. https://dx.doi.org/10.3389/fonc.2019.00889.

63. de Boer NL, Rovers K, Burger JWA, et al. A population-based study on the prognostic impact of primary tumor sidedness in patients with peritoneal metastases from colon cancer. Cancer medicine. 2020; 9(16): 5851-9. https://dx.doi.org/10.1002/cam4.3243.

64. De Haas DDF, De Ruysscher DKM, Lambin P, et al. Concomitant hyperthermia and radiation therapy for treating locally advanced rectal cancer. Cochrane Database of Systematic Reviews. 2006; http://dx.doi.org/10.1002/14651858.CD006269.

65. De-Colle C, Weidner N, Heinrich V, et al. Hyperthermic chest wall re-irradiation in recurrent breast cancer: a prospective observational study. Hyperthermie und Rebestrahlung der Brustwand bei rezidivierendem Brustkrebs: eine prospektive Beobachtungsstudie. 2019; 195(4): 318-26. https://dx.doi.org/10.1007/s00066-018-1414-z.

66. de Graaf-Strukowska L, van der Zee J, van Putten W, Senan S. Factors influencing the outcome of radiotherapy in malignant mesothelioma of the pleura--a single-institution experience with 189 patients. International journal of radiation oncology, biology, physics. 1999; 43(3): 511-6.

67. Delhorme J-B, Ohayon J, Gouy S, et al. Ovarian and peritoneal psammocarcinoma: Results of a multicenter study on 25 patients. European journal of surgical oncology : the journal of the European Society of Surgical Oncology and the British Association of Surgical Oncology. 2020; 46(5): 862-7. https://dx.doi.org/10.1016/j.ejso.2019.12.005.

68. Dharmaiah S, Zeng J, Rao VS, et al. Clinical and dosimetric evaluation of recurrent breast cancer patients treated with hyperthermia and radiation. International journal of hyperthermia : the official journal of European Society for Hyperthermic Oncology, North American Hyperthermia Group. 2019; 36(1): 986-92. https://dx.doi.org/10.1080/02656736.2019.1660810.

69. Djokic M, Badovinac D, Trotovsek B, et al. A prospective phase ii study evaluating intraoperative electrochemotherapy of hepatocellular carcinoma. Cancers. 2020; 12(12): 1-14. http://dx.doi.org/10.3390/cancers12123778.

70. Eberle B, Puric E, Meister A, et al. Hyperthermia-radiotherapy in frail bladder cancer patients unfit for cystectomy or chemoradiotherapy. Radiotherapy and Oncology. 2019; 133(Supplement 1): 858. http://dx.doi.org/10.1016/S0167-8140%2819%2932010-9.

71. El Sharouni SY, Warlam-Rodenhuis CC, De Leeuw AAC, Bouma P, Heintz APM. Radiotherapy in combination with regional hyperthermia in treating cervical cancer patients. International journal of gynecological cancer. 1997; 7(Suppl 2): 38.

72. Evrard S, Desolneux G, Bellera C, et al. Systemic chemotherapy plus cetuximab after complete surgery in the treatment of isolated colorectal peritoneal carcinoma: COCHISE phase II clinical trial. BMC research notes. 2019; 12(1): 450. <https://dx.doi.org/10.1186/s13104-019-4476-9>.

73. Fang H, Zhang Y, Wu Z, et al. Regional Hyperthermia Combined with Chemotherapy in Advanced Gastric Cancer. Open medicine (Warsaw, Poland). 2019; 14: 85-90. https://dx.doi.org/10.1515/med-2019-0012.

74. Franchi F, Grassi P, Ferro D, et al. Antiangiogenic metronomic chemotherapy and hyperthermia in the palliation of advanced cancer. European journal of cancer care. 2007; 16(3): 258-62.

75. Franckena M, Stalpers LJ, Koper PC, et al. Long-term improvement in treatment outcome after radiotherapy and hyperthermia in locoregionally advanced cervix cancer: an update of the dutch deep hyperthermia trial. International Journal of Radiation Oncology, Biology, Physics. 2008; 70(4): 1176-82. 10.1016/j.ijrobp.2007.07.2348.

76. Franckena M, Lutgens LC, Koper PC, et al. Radiotherapy and hyperthermia for treatment of primary locally advanced cervix cancer: results in 378 patients. International Journal of Radiation Oncology, Biology, Physics. 2009; 73(1): 242-50. https://dx.doi.org/10.1016/j.ijrobp.2008.03.072.

77. Gani C, Schroeder C, Heinrich V, et al. Long-term local control and survival after preoperative radiochemotherapy in combination with deep regional hyperthermia in locally advanced rectal cancer. International journal of hyperthermia : the official journal of European Society for Hyperthermic Oncology, North American Hyperthermia Group. 2016; 32(2): 187-92. https://dx.doi.org/10.3109/02656736.2015.1117661.

78. Gani C, Lamprecht U, Voigt O, et al. Radiochemotherapy and hyperthermia in locally advanced rectal cancer - A prospective phase II trial. Radiotherapy and Oncology. 2019; 133(Supplement 1): 266. http://dx.doi.org/10.1016/S0167-8140%2819%2930932-6.

79. Ganul V. The role of different preoperative treatment modality in esophageal carcinoma management. European journal of cancer. 1999; 35(Suppl 4): 145.

80. Garcia KM, Flores KM, Ruiz A, Gonzalez FL, Rodriguez AM. Pseudomyxoma Peritonei: Case Report and Literature Review. Journal of gastrointestinal cancer. 2019; 50(4): 1037-42. https://dx.doi.org/10.1007/s12029-018-00192-8.

81. Guo J, Zhu J, Sheng X, et al. Intratumoral injection of dendritic cells in combination with local hyperthermia induces systemic antitumor effect in patients with advanced melanoma. International Journal of Cancer. 2007; 120(11): 2418-25. http://dx.doi.org/10.1002/ijc.22551.

82. Hamza A, Zazo A, Alrifai MK, et al. Benign multicystic peritoneal mesothelioma (BMPM) presenting with ambiguous symptoms: A rare case report. Annals of medicine and surgery 2020; 61: 85-7. https://dx.doi.org/10.1016/j.amsu.2020.12.022.

83. Harima Y, Nagata K, Harima K, et al. Bax and Bcl-2 protein expression following radiation therapy versus radiation plus thermoradiotherapy in stage IIIB cervical carcinoma. Cancer. 2000; 88(1): 132‐8.

84. Harima Y, Nagata K, Harima K, Ostapenko VV, Tanaka Y, Sawada S. A randomized clinical trial of radiation therapy versus thermoradiotherapy in stage IIIB cervical carcinoma. International journal of hyperthermia. 2001; 17(2): 97‐105. https://dx.doi.org/10.1080/02656730010001333.

85. Harima Y, Nagata K, Harima K, Ostapenko VV, Tanaka Y, Sawada S. A randomized clinical trial of radiation therapy versus thermoradiotherapy in stage IIIB cervical carcinoma. International journal of hyperthermia. 2009; 25(5): 338‐43. https://dx.doi.org/10.1080/02656730903092018.

86. Harima Y, Ohguri T, Imada H, et al. A multicentre randomised clinical trial of chemoradiotherapy plus hyperthermia versus chemoradiotherapy alone in patients with locally advanced cervical cancer. International journal of hyperthermia. 2016; 32(7): 801‐8. https://dx.doi.org/10.1080/02656736.2016.1213430.

87. He M, Sun J, Zhao D, et al. Modified-FOLFIRINOX combined with deep regional hyperthermia in pancreatic cancer: a retrospective study in Chinese patients. International journal of hyperthermia : the official journal of European Society for Hyperthermic Oncology, North American Hyperthermia Group. 2019; 36(1): 394-402. https://dx.doi.org/10.1080/02656736.2019.1579371.

88. Hendricks A, Boerner K, Germer C-T, Wiegering A. Desmoplastic Small Round Cell Tumors: A review with focus on clinical management and therapeutic options. Cancer treatment reviews. 2021; https://dx.doi.org/10.1016/j.ctrv.2020.102140.

89. Hendricksen K. Device-assisted intravesical therapy for non-muscle invasive bladder cancer. Translational andrology and urology. 2019; 8(1): 94-100. https://dx.doi.org/10.21037/tau.2018.09.09.

90. Hu Y, Li Z, Mi DH, et al. Chemoradiation combined with regional hyperthermia for advanced oesophageal cancer: a systematic review and meta-analysis. Journal of clinical pharmacy and therapeutics. 2017; 42(2): 155-64. https://dx.doi.org/10.1111/jcpt.12498.

91. Huilgol NG. A phase I study to study arsenic trioxide with radiation and hyperthermia in advanced head and neck cancer. International journal of hyperthermia : the official journal of European Society for Hyperthermic Oncology, North American Hyperthermia Group. 2006; 22(5): 391-7.

92. Huilgol NG, Gupta S, Sridhar CR. Hyperthermia with radiation in the treatment of locally advanced head and neck cancer: a report of randomized trial. Journal of cancer research and therapeutics. 2010; 6(4): 492‐6. https://dx.doi.org/10.4103/0973-1482.77101.

93. Hurwitz MD, Kaplan ID, Hansen JL, et al. Association of rectal toxicity with thermal dose parameters in treatment of locally advanced prostate cancer with radiation and hyperthermia. International journal of radiation oncology, biology, physics. 2002; 53(4): 913-8.

94. Ichikawa D, Yamaguchi T, Yoshioka Y, Sawai K, Takahashi T. Prognostic evaluation of preoperative combined treatment for advanced cancer in the lower rectum with radiation, intraluminal hyperthermia, and 5-fluorouracil suppository. American journal of surgery. 1996; 171(3): 346‐50. https://dx.doi.org/10.1016/S0002-9610(97)89639-0.

95. Iizumi T, Shimizu S, Numajiri H, et al. Large Malignant Fibrous Histiocytoma Treated with Hypofractionated Proton Beam Therapy and Local Hyperthermia. International journal of particle therapy. 2019; 6(1): 35-41. https://dx.doi.org/10.14338/IJPT-18-00046.1.

96. Ishikawa T, Kokura S, Sakamoto N, et al. Phase II trial of combined regional hyperthermia and gemcitabine for locally advanced or metastatic pancreatic cancer. International journal of hyperthermia : the official journal of European Society for Hyperthermic Oncology, North American Hyperthermia Group. 2012; 28(7): 597-604. https://dx.doi.org/10.3109/02656736.2012.695428.

97. Issels RD, Lindner LH, von Bergwelt-Baildon M, et al. Systemic antitumor effect by regional hyperthermia combined with low-dose chemotherapy and immunologic correlates in an adolescent patient with rhabdomyosarcoma - a case report. International journal of hyperthermia : the official journal of European Society for Hyperthermic Oncology, North American Hyperthermia Group. 2020; 37(1): 55-65. https://dx.doi.org/10.1080/02656736.2019.1709666.

98. Iwahashi M, Tanimura H, Nakamori M, et al. Clinical evaluation of hepatic arterial infusion of low dose-CDDP and 5-FU with hyperthermotherapy: a preliminary study for liver metastases from esophageal and gastric cancer. Hepato-gastroenterology. 1999; 46(28): 2504-10.

99. Jones EL, Samulski TV, Dewhirst MW, et al. A pilot Phase II trial of concurrent radiotherapy, chemotherapy, and hyperthermia for locally advanced cervical carcinoma. Cancer. 2003; 98(2): 277-82.

100. Jow W. A novel targeted cluster tissue ablation combined with thermodilatation therapy lowers PSA in 18 men with BPH & prostate cancer and emerges as an innovative focal therapy for prostate cancer. International Journal of Urology. 2019; 26(Supplement 2): 213. http://dx.doi.org/10.1111/iju.14063.

101. Kamisawa T, Tu Y, Egawa N, et al. Thermo-chemo-radiotherapy for advanced bile duct carcinoma. World journal of gastroenterology. 2005; 11(27): 4206-9.

102. Kang MK, Kim MS, Kim JH. Clinical outcomes of mild hyperthermia for locally advanced rectal cancer treated with preoperative radiochemotherapy. International journal of hyperthermia : the official journal of European Society for Hyperthermic Oncology, North American Hyperthermia Group. 2011; 27(5): 482-90. https://dx.doi.org/10.3109/02656736.2011.563769.

103. Kato T, Fujii T, Ide M, et al. Effect of long interval between hyperthermochemoradiation therapy and surgery for rectal cancer on apoptosis, proliferation and tumor response. Anticancer research. 2014; 34(6): 3141-6.

104. Kim SW, Yea JW, Kim JH, Gu MJ, Kang MK. Selecting patients for hyperthermia combined with preoperative chemoradiotherapy for locally advanced rectal cancer. International Journal of Clinical Oncology. 2018; 23(2): 287-97. http://dx.doi.org/10.1007/s10147-017-1213-z.

105. Kitamura K, Kuwano H, Araki K, et al. Clinicopathologic features of patients with oesophageal cancer obtaining a histological complete response for preoperative hyperthermo-chemo-radiotherapy. International journal of hyperthermia : the official journal of European Society for Hyperthermic Oncology, North American Hyperthermia Group. 1998; 14(3): 233-43.

106. Kok HP, Cressman ENK, Ceelen W, et al. Heating technology for malignant tumors: a review. International journal of hyperthermia : the official journal of European Society for Hyperthermic Oncology, North American Hyperthermia Group. 2020; 37(1): 711-41. https://dx.doi.org/10.1080/02656736.2020.1779357.

107. Kok HP, Beck M, Loke DR, et al. Locoregional peritoneal hyperthermia to enhance the effectiveness of chemotherapy in patients with peritoneal carcinomatosis: a simulation study comparing different locoregional heating systems. International journal of hyperthermia : the official journal of European Society for Hyperthermic Oncology, North American Hyperthermia Group. 2020; 37(1): 76-88. https://dx.doi.org/10.1080/02656736.2019.1710270.

108. Kong F, Nie Z, Liu Z, Hou S, Ji J. Effects of thermal therapy combined with pamidronate disodium on pain associated with bone metastases: a randomized control trial (RCT) study. Biomedical research (india). 2017; 28(21): 9286‐90.

109. Kouloulias V, Triantopoulou S, Uzunoglou N, et al. Hyperthermia Is Now Included in the NCCN Clinical Practice Guidelines for Breast Cancer Recurrences: An Analysis of Existing Data. Breast care (Basel, Switzerland). 2015; 10(2): 109-16. https://dx.doi.org/10.1159/000376594.

110. Kroesen M, Mulder HT, van Holthe JML, et al. Confirmation of thermal dose as a predictor of local control in cervical carcinoma patients treated with state-of-the-art radiation therapy and hyperthermia. Radiotherapy and oncology : journal of the European Society for Therapeutic Radiology and Oncology. 2019; 140: 150-8. https://dx.doi.org/10.1016/j.radonc.2019.06.021.

111. Kroesen M, Mulder HT, van Holthe JML, et al. The Effect of the Time Interval Between Radiation and Hyperthermia on Clinical Outcome in 400 Locally Advanced Cervical Carcinoma Patients. Frontiers in oncology. 2019; 9: 134. https://dx.doi.org/10.3389/fonc.2019.00134.

112. Kuwano H, Sumiyoshi K, Watanabe M, et al. Preoperative hyperthermia combined with chemotherapy and irradiation for the treatment of patients with esophageal carcinoma. Tumori. 1995; 81(1): 18-22. <https://doi.org/10.1177/030089169508100105>

113. Lamprecht U, Heinrich V, Gani C, et al. Deep regional hyperthermia with preoperative radiochemotherapy in locally advanced rectal cancer, a prospective phase II trial. Radiotherapy and Oncology. 2021; 159: 155-60. http://dx.doi.org/10.1016/j.radonc.2021.03.011.

114. Letica-Kriegel AS, Leinwand JC, Sonett JR, et al. 50 Patients with Malignant Mesothelioma of Both the Pleura and Peritoneum: A Single-Institution Experience. Annals of surgical oncology. 2020; 27(1): 205-13. https://dx.doi.org/10.1245/s10434-019-07409-5.

115. Lindner LH. [Multimodal treatment of sarcomas: standards and new aspects in pharmacological and radio-oncological treatment]. Multimodale Therapien bei Sarkomen: Standards und Neues in der medikamentosen und radioonkologischen Behandlung. 2019; 90(6): 457-61. https://dx.doi.org/10.1007/s00104-019-0958-5.

116. Lindner LH, Blay J-Y, Eggermont AMM, Issels RD. Perioperative chemotherapy and regional hyperthermia for high-risk adult-type soft tissue sarcomas. European journal of cancer. 2021; 147: 164-9. https://dx.doi.org/10.1016/j.ejca.2021.02.002.

117. Linthorst M, Baaijens M, Wiggenraad R, et al. Local control rate after the combination of re-irradiation and hyperthermia for irresectable recurrent breast cancer: Results in 248 patients. Radiotherapy and oncology : journal of the European Society for Therapeutic Radiology and Oncology. 2015; 117(2): 217-22. https://dx.doi.org/10.1016/j.radonc.2015.04.019.

118. Liu Z. Clinical effects of high frequency hyperthermia-assisted irinotecan chemotherapy on patients with middle and advanced colorectal cancer and its safety assessment. Oncology letters. 2019; 17(1): 215-20. https://dx.doi.org/10.3892/ol.2018.9574.

119. Liu X, Tang M. Effect of early body cavity continuous circulation hyperthermia perfusion chemotherapy combined with systemic chemotherapy (and nursing) on survival rate and serum tumor markers in patients with advanced gastric cancer. European journal of inflammation. 2020; https://dx.doi.org/10.1177/2058739220942339.

120. Lloret M, Garcia-Cabrera L, Hernandez A, Santana N, Lopez-Molina L, Lara PC. Feasibility of a deep hyperthermia and radiotherapy programme for advanced tumors: first Spanish experience. Clinical & translational oncology : official publication of the Federation of Spanish Oncology Societies and of the National Cancer Institute of Mexico. 2019; 21(12): 1771-75. https://dx.doi.org/10.1007/s12094-019-02097-9.

121. Lutgens L, Lammering GL, Pijls-Johannesma M, et al. Combined use of hyperthermia and radiation therapy for treating locally advanced cervical cancer. Cochrane Database of Systematic Reviews. 2007; http://dx.doi.org/10.1002/14651858.CD006377.

122. Lyu X, Zheng D, Zhang H, et al. Hyperthermia improves immune function and radiotherapy efficacy in patients with post-operative recurrent gastric cancer. Hepato-gastroenterology. 2014; 61(136): 2428‐33.

123. Ma Q, Jiang L, Bonda S, Luo D, Zhang W. A rare case of hepatic sarcomatoid carcinoma: exceeding expectations in a stage IV primary hepatic sarcomatoid carcinoma patient. International journal of clinical and experimental pathology. 2019; 12(1): 378-83.

124. Maluta S, Dall'Oglio S, Romano M, et al. Conformal radiotherapy plus local hyperthermia in patients affected by locally advanced high risk prostate cancer: preliminary results of a prospective phase II study. International journal of hyperthermia : the official journal of European Society for Hyperthermic Oncology, North American Hyperthermia Group. 2007; 23(5): 451-6.

125. Maluta S, Schaffer M, Pioli F, et al. Regional hyperthermia combined with chemoradiotherapy in primary or recurrent locally advanced pancreatic cancer : an open-label comparative cohort trial. Strahlentherapie und Onkologie. 2011; 187(10): 619‐25. https://dx.doi.org/10.1007/s00066-011-2226-6.

126. Merten R, Ott O, Haderlein M, et al. Long-Term Experience of Chemoradiotherapy Combined with Deep Regional Hyperthermia for Organ Preservation in High-Risk Bladder Cancer (Ta, Tis, T1, T2). The oncologist. 2019; 24(12): 1341-50. https://dx.doi.org/10.1634/theoncologist.2018-0280.

127. Mitsumori M, Zeng ZF, Oliynychenko P, et al. Regional hyperthermia combined with radiotherapy for locally advanced non-small cell lung cancers: a multi-institutional prospective randomized trial of the International Atomic Energy Agency. International journal of clinical oncology. 2007; 12(3): 192‐8. https://dx.doi.org/10.1007/s10147-006-0647-5.

128. Mittal BB, Zimmer MA, Sathiaseelan V, et al. Phase I/II trial of combined 131I anti-CEA monoclonal antibody and hyperthermia in patients with advanced colorectal adenocarcinoma. Cancer. 1996; 78(9): 1861-70.

129. Nakajima M, Kato H, Sakai M, et al. Planned Esophagectomy after Neoadjuvant Hyperthermo-Chemoradiotherapy using Weekly Low-Dose Docetaxel and Hyperthermia for Advanced Esophageal Carcinomas. Hepato-gastroenterology. 2015; 62(140): 887-91.

130. Nozato T, Koizumi T, Hayashi Y, et al. Thermochemoradiotherapy Using Superselective Intra-arterial Infusion for Patients With Oral Cancer With Cervical Lymph Node Metastases. Anticancer research. 2019; 39(3): 1365-73. https://dx.doi.org/10.21873/anticanres.13250.

131. Nozoe T, Kuwano H, Sadanaga N, Watanabe M, Yasuda M, Sugimachi K. Hyperthermia combined with chemotherapy and irradiation for the prolongation of the postoperative prognosis in the patients with esophageal carcinoma invading neighbouring structures. International surgery. 1996; 81(1): 21-6.

132. Oberacker E, Kuehne A, Oezerdem C, et al. Radiofrequency applicator concepts for thermal magnetic resonance of brain tumors at 297 MHz (7.0 Tesla). International journal of hyperthermia : the official journal of European Society for Hyperthermic Oncology, North American Hyperthermia Group. 2020; 37(1): 549-63. https://dx.doi.org/10.1080/02656736.2020.1761462.

133. Ohguri T, Imada H, Yahara K, et al. Concurrent chemoradiotherapy with gemcitabine plus regional hyperthermia for locally advanced pancreatic carcinoma: initial experience. Radiation medicine. 2008; 26(10): 587-96. https://dx.doi.org/10.1007/s11604-008-0279-y.

134. Okamoto A, Tsuruta K, Ishiwata J, Isawa T, Kamisawa T, Tanaka Y. Treatment of T3 and T4 carcinomas of the gallbladder. International surgery. 1996; 81(2): 130-5.

135. Oldenborg S, van Os R, Oei B, Poortmans P. Impact of Technique and Schedule of Reirradiation Plus Hyperthermia on Outcome after Surgery for Patients with Recurrent Breast Cancer. Cancers. 2019; https://dx.doi.org/10.3390/cancers11060782.

136. Osone K, Ogawa H, Katayama C, et al. Outcomes of surgical treatment in patients with anorectal fistula cancer. Surgical case reports. 2021; 7(1): 32. https://dx.doi.org/10.1186/s40792-021-01118-6.

137. Ostapenko VV, Tanaka H, Miyano M, et al. Immune-related effects of local hyperthermia in patients with primary liver cancer. Hepato-gastroenterology. 2005; 52(65): 1502-6.

138. Ott OJ, Schmidt M, Semrau S, et al. Chemoradiotherapy with and without deep regional hyperthermia for squamous cell carcinoma of the anus. Radiochemotherapie mit und ohne regionale Tiefenhyperthermie bei Plattenepithelkarzinomen des Anus. 2019; 195(7): 607-14. https://dx.doi.org/10.1007/s00066-018-1396-x.

139. Ott OJ, Gani C, Lindner LH, et al. Neoadjuvant Chemoradiation Combined with Regional Hyperthermia in Locally Advanced or Recurrent Rectal Cancer. Cancers. 2021; https://dx.doi.org/10.3390/cancers13061279.

140. Reijtenbagh DMW, Godart J, Mens J-WM, Heijkoop ST, Heemsbergen WD, Hoogeman MS. Patient-reported acute GI symptoms in locally advanced cervical cancer patients correlate with rectal dose. Radiotherapy and oncology : journal of the European Society for Therapeutic Radiology and Oncology. 2020; 148: 38-43. https://dx.doi.org/10.1016/j.radonc.2020.03.035.

141. Rizzo S. Survival of chemo-radiotherapy-treated and thermotherapy-treated patients with unresectable lung cancer. Oncology reports. 1998; 5(3): 667-71. https://doi.org/10.3892/or.5.3.667

142. Rogers SJ, Datta NR, Puric E, et al. The addition of deep hyperthermia to gemcitabine-based chemoradiation may achieve enhanced survival in unresectable locally advanced adenocarcinoma of the pancreas. Clinical and translational radiation oncology. 2021; 27: 109-13. https://dx.doi.org/10.1016/j.ctro.2021.01.008.

143. Saeki H, Kawaguchi H, Kitamura K, Ohno S, Sugimachi K. Recent advances in preoperative hyperthermochemoradiotherapy for patients with esophageal cancer. Journal of surgical oncology. 1998; 69(4): 224-9.

144. Sakurai H, Hayakawa K, Mitsuhashi N, et al. Effect of hyperthermia combined with external radiation therapy in primary non-small cell lung cancer with direct bony invasion. International journal of hyperthermia : the official journal of European Society for Hyperthermic Oncology, North American Hyperthermia Group. 2002; 18(5): 472-83.

145. Sano F, Washio T, Matsumae M. Measurements of Specific Heat Capacities Required to Build Computer Simulation Models for Laser Thermotherapy of Brain Lesions. The Tokai journal of experimental and clinical medicine. 2019; 44(4): 80-4.

146. Schaffer M, Krych M, Pachmann S, et al. Feasibility and morbidity of combined hyperthermia and radiochemotherapy in recurrent rectal cancer--preliminary results. Onkologie. 2003; 26(2): 120-4.

147. Schullian P, Johnston EW, Putzer D, Eberle G, Laimer G, Bale R. Stereotactic radiofrequency ablation of subcardiac hepatocellular carcinoma: a case-control study. International journal of hyperthermia. 2019; 36(1): 876‐85. https://dx.doi.org/10.1080/02656736.2019.1648886.

148. Shimomura O, Oda T, Hashimoto S, et al. Survival impact on triple-modal strategy comprising hyperthermia, external radiation, and chemotherapy for unresectable locally advanced (UR-LA) pancreatic ductal adenocarcinoma. Surgical Oncology. 2021; <http://dx.doi.org/10.1016/j.suronc.2021.101542>.

149. Silva ACd, Oliveira TR, Mamani JB, et al. Magnetohyperthermia for treatment of gliomas: experimental and clinical studies. Einstein (Sao Paulo, Brazil). 2010; 8(3): 361-7. <https://dx.doi.org/10.1590/S1679-45082010RW1757>.

150. Sneed PK. Survival benefit of hyperthermia in a prospective randomized trial of brachyotherapy boost +/- hyperthermia for glyoblastoma multiforme. International journal of radiation oncology, biology, physics. 1996; 36(1): 159.

151. Spalek MJ, Borkowska AM, Telejko M, et al. The Feasibility Study of Hypofractionated Radiotherapy with Regional Hyperthermia in Soft Tissue Sarcomas. Cancers. 2021; https://dx.doi.org/10.3390/cancers13061332.

152. Sridhar PS, Abilash GH, Senapati MR, et al. SBRT and Hyperthermia in Hepatocellular Carcinoma - Tertiary Cancer Centre Experience. International Journal of Radiation Oncology Biology Physics. 2019; 105(1 Supplement): 228-9. http://dx.doi.org/10.1016/j.ijrobp.2019.06.1991.

153. Stoetzer O, Salat C, Di Gioia D, et al. Post-Neoadjuvant Gemcitabine and Cisplatin with Regional Hyperthermia for Patients with Triple-Negative Breast Cancer and Non-pCR after Neoadjuvant Chemotherapy: A Single-Institute Experience. Breast Care. 2020. http://dx.doi.org/10.1159/000507473.

154. Stoetzer O, Di Gioia D, Issels Rolf D, et al. Post-Neoadjuvant Gemcitabine and Cisplatin with Regional Hyperthermia for Patients with Triple-Negative Breast Cancer and Non-pCR after Neoadjuvant Chemotherapy: A Single-Institute Experience. Breast Care. 2021; 16(2): 173-80. https://dx.doi.org/10.1159/000507473.

155. Striefler JK, Brandes F, Baur A, et al. Combination therapy with Olaratumab/doxorubicin in advanced or metastatic soft tissue sarcoma -a single-Centre experience. BMC cancer. 2020; 20(1): 68. https://dx.doi.org/10.1186/s12885-020-6551-y.

156. Sugarbaker PH, Hassanein MT. Revised management of advanced primary colon cancer: Case series of 2 patients. International Journal of Surgery Case Reports. 2019; 55: 233-8. http://dx.doi.org/10.1016/j.ijscr.2019.01.046.

157. Sugarbaker PH. Total pelvic exenterative surgery in patients with peritoneal metastases from appendiceal neoplasms. A case series of 2 patients. International journal of surgery case reports. 2019; 65: 279-83. https://dx.doi.org/10.1016/j.ijscr.2019.10.064.

158. Tran S, Puric E, Walser M, et al. Early results and volumetric analysis after spot-scanning proton therapy with concomitant hyperthermia in large inoperable sacral chordomas. The British journal of radiology. 2020; https://dx.doi.org/10.1259/bjr.20180883.

159. Tsutsumi S, Tabe Y, Fujii T, et al. Tumor response and negative distal resection margins of rectal cancer after hyperthermochemoradiation therapy. Anticancer research. 2011; 31(11): 3963-7.

160. Unsoeld M, Lamprecht U, Traub F, et al. MR Thermometry Data Correlate with Pathological Response for Soft Tissue Sarcoma of the Lower Extremity in a Single Center Analysis of Prospectively Registered Patients. Cancers. 2020; https://dx.doi.org/10.3390/cancers12040959.

161. Van Vulpen M, De Leeuw JRJ, Van Gellekom MPR, et al. A prospective quality of life study in patients with locally advanced prostate cancer, treated with radiotherapy with or without regional or interstitial hyperthermia. International journal of hyperthermia : the official journal of European Society for Hyperthermic Oncology, North American Hyperthermia Group. 2003; 19(4): 402-13.

162. Wang YY, Lin SX, Yang GQ, Liu HC, Sun DN, Wang YS. Clinical efficacy of CyberKnife combined with chemotherapy and hyperthermia for advanced non-small cell lung cancer. Molecular and Clinical Oncology. 2013; 1(3): 527-30. http://dx.doi.org/10.3892/mco.2013.95.

163. Yang W-H, Xie J, Lai Z-Y, et al. Radiofrequency deep hyperthermia combined with chemotherapy in the treatment of advanced non-small cell lung cancer. Chinese medical journal. 2019; 132(8): 922-7. https://dx.doi.org/10.1097/CM9.0000000000000156.

164. Yang Y, Zhang L, Qi R, et al. Treatment of high risk human papillomavirus infection in low grade cervical squamous intraepithelial lesion with mild local thermotherapy: Three case reports. Medicine. 2020; https://dx.doi.org/10.1097/MD.0000000000021005.

165. Zagar TM, Vujaskovic Z, Formenti S, et al. Two phase I dose-escalation/pharmacokinetics studies of low temperature liposomal doxorubicin (LTLD) and mild local hyperthermia in heavily pretreated patients with local regionally recurrent breast cancer. International journal of hyperthermia : the official journal of European Society for Hyperthermic Oncology, North American Hyperthermia Group. 2014; 30(5): 285-94. https://dx.doi.org/10.3109/02656736.2014.936049.

166. Zhao L, Li J, Bai C, Nie Y, Lin G. Multi-Modality Treatment for Patients With Metastatic Gastric Cancer: A Real-World Study in China. Frontiers in Oncology. 2019; 9: 1155. http://dx.doi.org/10.3389/fonc.2019.01155.

167. Zhou L, Zhang T, Sun Y, et al. Effect of preoperative infusion chemotherapy combined with hyperthermia on sPD-L1 and CEA levels and overall survival of elderly patients undergoing radical resection of lung cancer. Journal of B.U.ON. : official journal of the Balkan Union of Oncology. 2019; 24(2): 572-7.

168. Zwirner K, Bonomo P, Lamprecht U, Zips D, Gani C. External validation of a rectal cancer outcome prediction model with a cohort of patients treated with preoperative radiochemotherapy and deep regional hyperthermia. International journal of hyperthermia : the official journal of European Society for Hyperthermic Oncology, North American Hyperthermia Group. 2018; 34(4): 455-60. https://dx.doi.org/10.1080/02656736.2017.1338364.

169. Datta NR, Bodis S. Does hyperthermia clinically alter the Î±/Î²? Insights from thermoradiotherapy vs. radiotherapy trials. Radiotherapy and oncology. 2019; https://dx.doi.org/10.1016/S0167-8140(19)31500-2.

170. Joiner JB, Pylayeva-Gupta Y, Dayton PA. Focused Ultrasound for Immunomodulation of the Tumor Microenvironment. Journal of immunology. 2020; 205(9): 2327-41. https://dx.doi.org/10.4049/jimmunol.1901430.

171. Thomsen A, Voglstatter M, Bieber B, et al. Multipotent mesenchymal stromal cells are sensitive to thermic stress-potential implications for therapeutic hyperthermia. International Journal of Hyperthermia. 2020; 37(1): 430-41. http://dx.doi.org/10.1080/02656736.2020.1758350.

172. Miao C-L, Chen X-B, Huang M, et al. Effects of cisplatin in combination with hyperthermia on biological characteristics of retroperitoneal liposarcoma. Chinese medical journal. 2020. http://dx.doi.org/10.1097/CM9.0000000000001326.

173. Feyerabend T, Steeves R, Jager B, et al. Local hyperthermia, hyperfractionated radiation, and cisplatin in preirradiated recurrent lymph node metastases of recurrent head and neck cancer. International journal of oncology. 1997; 10(3): 591-5. https://doi.org/10.3892/ijo.10.3.591

174. Abdel‐Rahman O, Elsayed Z, Mohamed H, Eltobgy M. Radical multimodality therapy for malignant pleural mesothelioma. Cochrane Database of Systematic Reviews. 2018; https://dx.doi.org/10.1002/14651858.CD012605.pub2.

175. Aquilina K, Hill CS, Devesa SC, Borg A, Ince W. A systematic review of ongoing clinical trials in optic pathway gliomas. Child's Nervous System. 2020; 36(9): 1869-86. http://dx.doi.org/10.1007/s00381-020-04724-1.

176. Bogovic J, Douwes F, Muravjov G, Istomin J. Posttreatment histology and microcirculation status of osteogenic sarcoma after a neoadjuvant chemo- and radiotherapy in combination with local electromagnetic hyperthermia. Onkologie. 2001; 24(1): 55-8.

177. Depla AL, Scharloo-Karels CH, de Jong MAA, et al. Treatment and prognostic factors of radiation-associated angiosarcoma (RAAS) after primary breast cancer: a systematic review. European journal of cancer (Oxford, England : 1990). 2014; 50(10): 1779-88. https://dx.doi.org/10.1016/j.ejca.2014.03.002.

178. Furness S, Glenny AM, Worthington HV, et al. Interventions for the treatment of oral cavity and oropharyngeal cancer: chemotherapy. Cochrane Database of Systematic Reviews. 2011; https://dx.doi.org/10.1002/14651858.CD006386.pub3.

179. Goyal L, Chen CT, Pierce TT, Deshpande V. Case 8-2021: A 34-year-old woman with cholangiocarcinoma. New England Journal of Medicine. 2021; 384(11): 1054-64. http://dx.doi.org/10.1056/NEJMcpc2027092.

180. Kim BH, Kim S, Shin KH, Chie EK, Kim JH, Kim K. Locoregionally recurrent breast cancer treated with postoperative or salvage radiotherapy. Radiotherapy and Oncology. 2019; 133(Supplement 1): 701. http://dx.doi.org/10.1016/S0167-8140%2819%2931697-4.

181. Klos D, Risko J, Lovecek M, et al. Trends in peritoneal surface malignancies: evidence from a Czech nationwide population-based study. World journal of surgical oncology. 2019; 17(1): 182. https://dx.doi.org/10.1186/s12957-019-1731-4.

182. Lin L, Tang C, Liang J, Wang X. Clinical Characteristics and Prognosis of Gastrointestinal Metastases in Solid Tumor Patients: A Retrospective Study and Review of Literatures. Analytical Cellular Pathology. 2019; http://dx.doi.org/10.1155/2019/4508756.

183. Rim CH, Shin IS, Park S, Lee HY. Benefits of local consolidative treatment in oligometastases of solid cancers: a stepwise-hierarchical pooled analysis and systematic review. Npj precision oncology. 2021; https://dx.doi.org/10.1038/s41698-020-00141-4.

184. Schaper T, Gross B, Franzmann L, Darsow M. Using a new controlled thermotherapy (Hilotherapy) during chemotherapy prevents chemotherapy induced polyneuropathy (CIPN). Annals of Oncology. 2020; 31(Supplement 2): 83-4. http://dx.doi.org/10.1016/j.annonc.2020.03.125.

185. Schreckenbach T, Hubert H, Schnitzbauer AA, et al. Surgical resection of neuroendocrine tumor liver metastases as part of multimodal treatment strategies: A propensity score matching analysis. European Journal of Surgical Oncology. 2019; 45(5): 808-15. http://dx.doi.org/10.1016/j.ejso.2018.12.022.

186. Villanueva A. Hepatocellular carcinoma. New England Journal of Medicine. 2019; 380(15): 1450-62. http://dx.doi.org/10.1056/NEJMra1713263.

187. Xu J, Zhang ZZ, Lin TL, Cao H, Yang HK. Meta-analysis of irinotecan monotherapy versus irinotecan-based combined second-line therapy for the treatment of advanced gastric cancer. International Journal of Clinical and Experimental Medicine. 2016; 9(7): 13712-22.

188. Zhang Z, Mei Y, Cheng H, Wu X. Portal vein thrombosis secondary to postoperative gastric cancer: Report of two cases. The Journal of international medical research. 2020; https://dx.doi.org/10.1177/0300060519892417.
